# Deep learning interpretability in neuroimaging: A comprehensive survey and methodological recommendations

**DOI:** 10.1162/IMAG.a.1129

**Published:** 2026-03-10

**Authors:** Md Mahfuzur Rahman, Vince Calhoun, Sergey Plis

**Affiliations:** Department of Computer Science, Georgia State University, Atlanta, GA, United States; Tri-Institutional Center for Translational Research in Neuroimaging and Data Science (TReNDS) Georgia State University, Georgia Institute of Technology, Emory University Atlanta, Atlanta, GA, United States

**Keywords:** deep learning, interpretability, neuroimaging, brain dynamics, psychiatric disorders

## Abstract

Deep learning (DL) models have experienced a surge in popularity due to their capacity to directly learn from raw data in an end-to-end paradigm without relying on a separate feature extraction process that may be based on restrictive assumptions. The neuroimaging community has enthusiastically embraced DL as it strives to learn biomarkers from complex, multivariate, multimodal datasets. However, a broad replacement of human intelligence with DL in clinical environments is yet far from realization. One of the major obstacles to this transition is the opacity of DL models. A deep understanding of models is essential for their effective deployment in safety-critical domains such as healthcare, where transparency and trust hold substantial significance. We provide a comprehensive review of the interpretability literature, specifically focusing on the current status of DL interpretability in neuroimaging studies. Ultimately, we highlight strategies and insights necessary for successfully integrating DL technology in characterizing and addressing mental disorders.

## 1 Introduction

Neuroimaging techniques play a critical role in understanding the human brain and have the potential to provide valuable insights into brain function, structure, and connectivity (Linden, 2012). These techniques also hold promise for predicting behavior and identifying biomarkers of brain disorders, which are often associated with disrupted connectivity (Poldrack & Farah, 2015; Van Den Heuvel et al., 2013). However, analyzing high-dimensional neuroimaging data, identifying meaningful biomarkers, and interpreting these findings in a comprehensible and clinically translatable manner are challenging tasks.

In the mid-90s, statistical parametric mapping and voxel-based morphometry (VBM) analysis methods (Friston et al., 1994) gained popularity for their ability to generate detailed spatial maps of brain structure and function. However, these methods, also known as mass-univariate methods (Davatzikos, 2019), were primarily focused on group-level analysis (Ashburner & Friston, 2000) and were unable to capture individual-level disease effects, brain activations, and other discriminative imaging patterns (Davatzikos, 2004). As a result, these voxel-based methods were not suitable for individual-level biomarker discovery or diagnostic and prognostic decision making. Given these limitations, the field of neuroimaging witnessed a significant shift in the early 2000s with the adoption of machine learning (ML) methods (Davatzikos, 2019), which signified a transition from mass univariate to multi-variate approaches. ML models hold significant promise in enabling machines to understand disorder-specific functional dynamics or anatomical alterations based on holistic patterns within the data on an individual level (Hastie et al., 2009).

While standard machine learning (SML) models exhibit reasonably good discriminative performance, these models heavily rely on a pre-requisite feature extraction step (Khazaee et al., 2016). Numerous studies have used a variety of features such as the volume of gray matter, white matter, cerebrospinal fluid, the area of the left hippocampus, and the area of the right hippocampus (Farhan et al., 2014), gray matter densities (Schnack et al., 2014), the cortical thickness, and surface area (Xiao et al., 2019) for structural magentic resonance imaging (sMRI) images. Functional network connectivity, measured through Pearson’s correlation coefficients, was used as features for a range of classifiers in functional magentic resonance imaging (fMRI) studies (Rashid et al., 2016; Shen et al., 2010; Venkataraman et al., 2012; Zeng et al., 2018). Even though feature engineering can result in class-discriminative features, it also has its limitations. It requires expert knowledge and depends heavily on certain assumptions, rather than allowing for the data-driven discovery of representative features. Moreover, when trained on raw data, the performance of SML models tends to decline significantly due to their inability to adaptively learn features directly from the data (Oh et al., 2019; Plis et al., 2014; Rahman et al., 2022).

Learning directly from data may uncover valuable patterns and expedite diagnostic and prognostic decision making in clinical practices. Deep learning (DL) has gained popularity due to its ability to learn from data without prior feature selection or intermediate intervention (Islam & Zhang, 2018; LeCun et al., 2015; Patel et al., 2016; Payan & Montana, 2015; Zhang et al., 2021b). Instead, DL models learn through varying levels of data abstraction using a series of nonlinear function applications. Following DL model’s striking performance in computer vision (Russakovsky et al., 2015) and language understanding (Hirschberg & Manning, 2015), the neuroimaging domain has also witnessed a surge of DL applications in recent years (Abrol et al., 2021; Mahmood et al., 2020; Mensch et al., 2017; Nie et al., 2016; Plis et al., 2014; Sarraf & Tofighi, 2016; Thomas et al., 2019; Yan et al., 2022).

There are diverse desiderata to consider when interpreting DL models (Lipton, 2018). For example, users may need to build trust, transfer learned skills to new situations, identify causal structures in the data, gain intuitive insights from models, and ensure fairness in the decisions these models make. Among these desiderata, a particularly crucial need in the healthcare field is to uncover scientifically or clinically valuable insights that models may have acquired during training (Ghorbani et al., 2020; Hicks et al., 2021). However, DL models learn functions that map an input to an output without explicitly explaining how these mappings are achieved. As a result, these models are often considered black boxes (Hart & Wyatt, 1990; McCoy et al., 2022). While DL models learn features and their relationships automatically, the connectivity of the underlying architecture, such as fully connected or convolutional layers, depends on a set of assumptions, also referred to as “inductive biases” (Goyal & Bengio, 2022), about the model and the nature of the input data. For convolutional layers, for example, these biases include assumptions such as hierarchical relationships between features, strong relationships among locally adjacent features, and the ability to detect specific patterns through weight sharing. Despite understanding the general learning mechanism of DL models, the specific associations they ultimately learn remain inexplicable. This inexplicability necessitates the urge for explanations in safety-critical domains such as healthcare and medicine. Indeed, the need for explanations arises from inadequate knowledge of the data, the distributional behavior of the associated generation mechanism, and a poor understanding of the model’s behavior during training. Also, the model may overfit and not generalize well to unseen subjects because it might learn artifactual associations for its predictions (Geirhos et al., 2020; Lapuschkin et al., 2019). Additionally, many challenges—such as overfitting, distributional instability, missing or incomplete data, and data imbalance—can limit the generalizability of both DL and traditional ML models (Martí-Juan et al., 2020). While building robust DL models may offer strong defenses against these issues, model interpretability methods that remain informative despite such issues can further increase trust and intelligibility. Thus, robust explainable models have the potential to satisfy additional interpretability desiderata—such as causality, safety, nondiscrimination, and justice—which are essential for the responsible and widespread deployment of DL models (Doshi-Velez & Kim, 2017; Lipton, 2018).

The field of *eXplainable Artificial Intelligence* (XAI) has made significant advancements over the past decade, resulting in the development of various methods, metrics, and theoretical insights. In this line, many earlier studies (Baehrens et al., 2010; Morch et al., 1995; Simonyan et al., 2013; Zeiler & Fergus, 2014) strived to explain these black boxes. To gain a comprehensive understanding of interpretability in AI, we recommend referring to the complementary literature reviews (Adadi & Berrada, 2018; Arrieta et al., 2020; Carvalho et al., 2019; Dwivedi et al., 2023; Gilpin et al., 2018; Guidotti et al., 2018; Linardatos et al., 2020; Lipton, 2018; Murdoch et al., 2019; Ras et al., 2022; Samek et al., 2021).

There are specialized literature reviews focusing on interpretable models in neuroimaging (Farahani et al., 2022; Huff et al., 2021; Taiyeb Khosroshahi et al., 2025; Thibeau-Sutre et al., 2023; Vieira et al., 2017; Viswan et al., 2024; Zhang et al., 2020b) and medicine (Nazir et al., 2023; Rasheed et al., 2022; Salahuddin et al., 2022; Van der Velden et al., 2022). These reviews provide invaluable insights, yet have certain limitations. For instance, very few delved into neuroimaging studies using DL technology exclusively, and many focused mainly on post hoc methods (Farahani et al., 2022; Taiyeb Khosroshahi et al., 2025), one task (e.g., Alzheimer’s disease classification) with an emphasis on limited XAI approaches (mainly post hoc) (Taiyeb Khosroshahi et al., 2025; Viswan et al., 2024). While a recent review on the applications of DL in neuroimaging (Munroe et al., 2024) shared valuable insights from a large number of studies, the reviewed post hoc and intrinsic methods are still limited. For example, papers using promising methods such as integrated gradients and SHAP were not covered. Other possibilities of DL explainability, such as “concept-based” and “example-based” explanations, were not discussed. Overall, the scope of earlier reviews is often restricted, leaving space for a broader spectrum of exploration.

Therefore, there is a need for a comprehensive guideline that aligns with existing work, such as the one proposed by Kohoutová et al. (2020). Such guideline could serve as a useful resource for the community and should encompass a broader understanding of interpretability, an introduction to commonly used methods, evaluation metrics, available software toolkits, and illustrative examples of their application in neuroimaging. Additionally, neuroimaging studies often select interpretability methods without considering underlying caveats. As new approaches emerge, many older approaches soon become inappropriate and lose their timeliness. Therefore, a comparative analysis of the relative merits and limitations of post hoc XAI methods in neuroimaging studies is timely and can offer valuable guidance for future directions in this field. The primary aim of this review is to provide a field guide for interpretable DL in neuroimaging, particularly for researchers new to this area of study.

We adopted the following search strategy: we searched Google Scholar and PubMed for relevant publications. Our search terms focused mainly on two areas: (1) the introduction to XAI methods, evaluation metrics, and available toolkits, and (2) deep learning interpretability in neuroimaging. For Explainable AI, we used the keywords “interpretable AI,” “Explainable AI,” and “interpretability in deep learning” to cover the relevant literature from the past decade, and we included approximately 122 papers from general XAI techniques, tools, and open resources, critical commentaries, design guidelines, and philosophies in the medical domain. For the deep learning interpretability review in neuroimaging, we used keywords such as “deep learning in neuroimaging,” “interpretability in neuroimaging,” “intrinsic interpretability in neuroimaging,” “explainability evaluation framework in neuroimaging,” “explainable AI brain MRI segmentation,” “explainable AI brain MRI reconstruction,” “explainable AI image-to-image translation in neuroimaging,” “explainable AI graph neural networks in (brain OR neuro) imaging,” and “attention brain explainable.” We also manually reviewed relevant articles and review papers to identify additional sources. We refined our focus to studies that used DL and explainability approaches. To accomplish this, we reviewed titles, abstracts, and full texts and selected approximately 409 neuroimaging studies published between 2017 and 2025 that applied XAI approaches. We also included insights from some review papers (Amorim et al., 2021; Champendal et al., 2023; Górriz et al., 2023; Graziani et al., 2023; Reyes et al., 2020; Viswan et al., 2024). To sort out the papers, we grouped them according to the XAI method and conducted further review and analysis.

We have organized this paper as follows: In Section 2.1, we introduce the XAI problem from a broad perspective and include a helpful categorization of interpretability methods. We compile several notable neuroimaging studies for each XAI approach. We also provide a brief overview of the main branches of interpretability approaches developed over the past decade and the underlying principles of each method. Additionally, as these approaches were mostly adapted from the computer vision domain, we provide some examples of visual explanations for natural images. In Section 3, we discuss the commonly used evaluation metrics and available software toolkits to help practitioners with appropriate XAI resources. In Section 4, we review the neuroimaging studies that utilize DL technology based on the categories of XAI methods.

We selected representative papers, as listed in Table 3, which we discuss in detail and provide some encouraging illustrations of how XAI concepts have been applied for novel insights. Tables 4, 5, 6, 7, and 8 summarize the other reviewed papers. In Section 5, we provide an overview of the review and conduct a meta-analysis to investigate the usage of the most widely adopted post hoc approaches, as well as the most frequently studied neuroimaging disorders and modalities (see Fig. 8) in the reviewed literature. In Section 5.1, we present a comparative discussion of relative advantages and disadvantages of feature extraction-based and DL-based approaches for neuroimaging data analysis. In Section 5.2, we provide a critical analysis of the reviewed papers and identify research gaps for future directions in light of the observations, concerns, and opinions often discussed in the XAI literature. To ensure clarity for the readers, we also catalog commentaries and takeaways from seminal XAI papers in Table 9. Section 5.3 discusses the relative advantages and disadvantages of post hoc XAI methods based on the reviewed XAI and neuroimaging literature to help practitioners make informed choices for their studies. Finally, in Section 5.4, we provide additional high-level recommendations for other avenues of future XAI practices in neuroimaging. We conclude with our final remarks in Section 6.

## 2 Explainable AI

To reliably leverage the full potential of AI systems, the field of XAI has received widespread attention over the past decade and has developed a plethora of methods and metrics (Adadi & Berrada, 2018; Arrieta et al., 2020; Gilpin et al., 2018; Guidotti et al., 2018; Lipton, 2018; Murdoch et al., 2019; Ras et al., 2022; Samek et al., 2021). Still, there is no formal definition of “explainability” or “interpretability” in the field of AI (Doshi-Velez & Kim, 2017; Lipton, 2018; Miller, 2019).

The terms “interpretability” and “explainability” have been used interchangeably by many researchers (Miller, 2019; Murdoch et al., 2019) and these terms convey different meanings in different domains (Graziani et al., 2023). However, many researchers (Lipton, 2018; Rudin, 2019) made a clear distinction of the terms as these terms address different aspects of understanding AI systems.

While the meaning of “interpretability” often relies on the application domain (Rudin, 2019) and the target audience (Carvalho et al., 2019), Arrieta et al. (2020) defined *interpretability* as “the ability to explain or to provide the meaning in understandable terms to a human.” The term *interpretability* is a passive characteristic of a model that indicates the extent to which the model makes sense to humans (Arrieta et al., 2020). For example, models such as sparse linear models and shallow decision trees can offer greater transparency, as their decision logic or feature importance can often be directly examined and understood by humans without the need for separate explanation tools. These models are often referred to as “interpretable models.”

However, *explainability* refers to the idea of using explanations as an interface between humans and the model, usually in a post hoc manner. These explanations serve as a proxy for the model’s decision-making process and are comprehensible to humans (Guidotti et al., 2018).

While interpretable models, such as decision trees or sparse linear models, offer inherent transparency by clearly showing how inputs influence predictions, “post hoc” explainability methods aim to extract insights from complex, black-box models whose internal decision processes are not readily understandable. Such insights may include feature sensitivity values, feature attributions, attention scores, or counterfactual explanations that help users understand, trust, or audit the model’s behavior. Interpretable models are associated with the idea of transparency and Lipton (2018) defines *transparency* from the following perspectives:

- *Simulatability:* A model is simulatable if a person can simulate the entire computation as done by the model within a reasonable amount of time. Given the subjective notion of time constraint and the limited human cognition, even dense linear models, deep decision trees might not be considered as “intrinsically interpretable.”
- *Decomposability:* A model is decomposable if its inputs, parameters, and calculations all are intuitively explainable. For example, the weights of a linear model may refer to the strengths of features associated with a target label. When a model relies on highly engineered uninterpretable or anonymous features, the features may no longer be meaningful. Again, from the model’s perspective, one caveat is that the weights in a linear model, though explainable, are highly subject to the pre-processing and the overall feature selection procedure.
- *Algorithmic transparency:* A model is algorithmically transparent if, for a given dataset, its training algorithm always converges to a unique global optimum solution. For example, linear models (e.g., linear regression, logistic regression) are algorithmically transparent because their training objectives are convex, which guarantees a deterministic solution for the same data. In contrast, DL models usually solve non-convex optimization problems, which can lead to different solutions even for the same data.

### 2.1 Taxonomy of interpretability methods

The concept of “interpretability” can be classified broadly into two categories based on its application scope: *global interpretability* and *local interpretability*. By analyzing a model’s behavior over the entire dataset, *global interpretability* methods look for general patterns that the model uses for predictions (Reyes et al., 2020). It has obvious advantage during model development to verify if the learned patterns are aligned with domain knowledge or if the model has acquired any biases from the training data (Reyes et al., 2020). As *global interpretability* is very hard to obtain because it requires building a relationship among all predictions made by the model, people often use local interpretability that deals with explaining model behavior for a single prediction. For example, *local interpretability* may try to explain why a particular subject has been diagnosed with Alzheimer’s disease (AD), by finding affected brain regions responsible for the decision.

Different approaches can be used to achieve model interpretability. There are many surveys on explainable AI (Arrieta et al., 2020; Carvalho et al., 2019; Dwivedi et al., 2023; Gilpin et al., 2018; Guidotti et al., 2018; Linardatos et al., 2020; Lipton, 2018; Murdoch et al., 2019) and explainable DL (Ras et al., 2022; Samek et al., 2021) that cover a large body of existing methods, metrics, and associated challenges. One approach to achieving interpretability is to construct an inherently interpretable model by using interpretable models such as sparse linear models or decision trees. However, these models may sacrifice their predictive ability to provide transparency and often may not satisfy a particular notion of transparency as discussed earlier (Lipton, 2018).

The second approach involves building a model that can generate explanations while making predictions. These explanations reveal patterns and relationships used for the predictions. This is a challenging task because it may require both true explanations and labeled samples to train the model simultaneously for prediction and explanation generation. We note that intrinsic methods to explain decisions of DL models do not make the models “inherently interpretable” (white boxes) (Graziani et al., 2023) in the same direct manner as sparse linear models or decision trees. Rather, these models (black boxes) jointly optimize for model performance and explanations (Ras et al., 2022) and offer additional layer of explanation fidelity. The built-in explainability of these “inherently explainable” models is an added feature of the model rather than a fundamental characteristic and the explanations still need to be validated in a post hoc manner to draw meaningful conclusions from them.

**Fig. 1.**
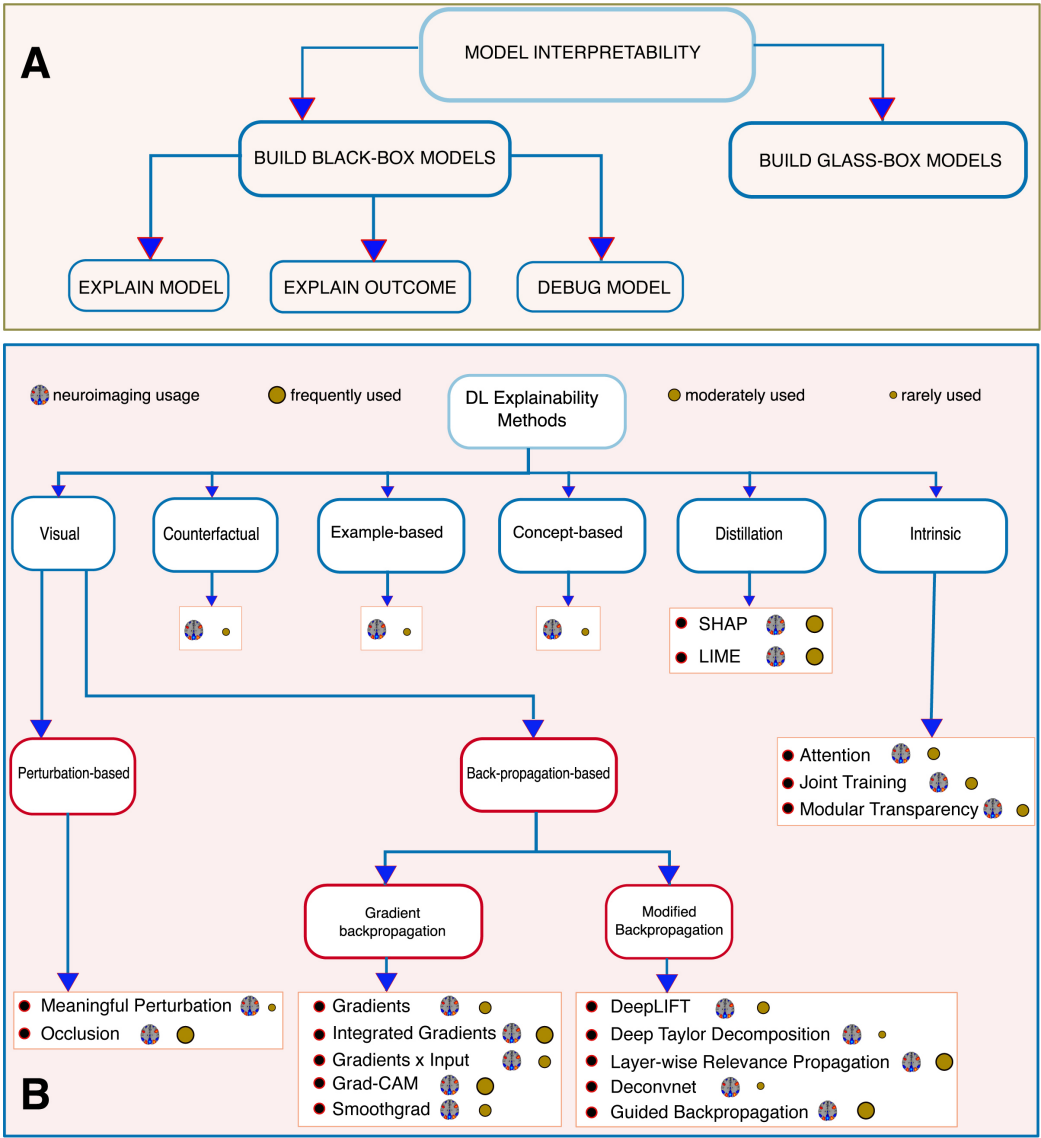
(A) Model transparency and different facets of XAI. (B) Taxonomy of Explainable AI and the level of usage in neuroimaging applications. We note that the categorization of XAI approaches based on their usage frequency is relative and depends solely on the papers reviewed in this manuscript.

The third approach, referred to as “post hoc” methods, utilizes separate explanation methods to generate explanations for individual model predictions. In medical imaging, local interpretability is more common, and post hoc XAI approaches have been used predominantly (Champendal et al., 2023). For a comprehensive understanding of the various aspects of the interpretability problem, we refer to *Panel A* of Figure 1. As we can see, the goal of interpretability from a holistic standpoint is to debug and validate models and to extract new domain-specific insights (Amorim et al., 2021) by probing different sub-categories of explanations: *model explanation* means explaining the overall logic of the model; *outcome explanation* means finding the correlation between individual inputs and corresponding decisions; *model debugging* or *model inspection* means explaining the behavioral change with changes in input and other parameters or explaining which parts of the model make specific micro-decisions (Guidotti et al., 2018).

In *Panel B* of the same figure, we present a taxonomy of interpretability methods similar to the one proposed by Ras et al. (2022). However, we further divided the two variants of backpropagation approaches: standard backpropagation and relevance backpropagation. While the taxonomy for XAI is still not conclusive and remains under refinement, we carefully curated approaches that fit well in the neuroimaging domain. The proposed taxonomy includes “counterfactual,” “example-based” (e.g., influence functions), and “concept-based” explanations as separate categories because they have their own space in neuroimaging. We note that not all XAI methods are equally applicable to all DL models or prediction tasks. Each method has its own goals, underlying assumptions, and implementation procedures for generating explanations. Some XAI methods are model-agnostic, whereas others are model-specific. Certain approaches—particularly intrinsic methods—are closely tied to a model’s architecture and training mechanism. Because of these differences in design and purpose, one method is rarely a direct substitute for another. The choice of method, therefore, depends on factors such as the model type, the nature of the task, computational constraints, and the desired form of explanation. Refer to Table 1, which compiles representative neuroimaging studies for each XAI method and serves as a quick reference for their applications in neuroimaging. We categorize and describe the main interpretability approaches as follows:

1. Visual Explanation Methods: Visual explanation methods refer to explanation approaches that produce direct visual outputs based on the original model’s internal representations or gradients, and whose primary mechanism for generating explanations is inherently visual, such as saliency maps and feature attribution maps. These methods highlight the discriminative regions of the input that influenced the model’s decision.
2. Distillation Methods: Distillation methods are model-agnostic and focus on building a separate, directly interpretable “transparent” model, which allows direct identification of the salient regions or decision rules that the original model used. The generated explanation is often presented as a visual summary of the distilled model’s learned features or decision rules, often highlighting important regions such as superpixels in the input space.
3. Intrinsic Methods: Intrinsic methods consider model interpretability during model design or training. This approach usually leads toward joint training for predictions and explanations or provides increased transparency.
4. Counterfactual Explanations: Counterfactual explanations (Mothilal et al., 2020; Wachter et al., 2017) do not explain the specific output. Instead, it explains in the form of hypothetical scenarios to provide a better understanding of how the decisions change over the input space (Dandl et al., 2020).
5. Example-based Explanations: This class of explanation strategies (Ilyas et al., 2022; Koh & Liang, 2017) attempts to find representative examples from the training set to explain a model outcome. It implies that the model has gathered relevant information from these representative samples to make the current decision.
6. Concept-based Explanations: This class of explanation (Kim et al., 2018) tests if the trained model learned some user-provided concepts and thus tries to explain the model behavior based on human-understandable concepts.

**Table 1.** Representative neuroimaging studies using popular XAI methods.

	Category						
	Visualization						
Interpretability method	Perturbation	Standard Backprop	Modified Backprop	Distillation	Intrinsic	Counterfactual Wachter et al. (2017) Dandl et al. (2020)	Representative studies in neuroimage
Occlusion Sensitivity (Zeiler & Fergus, 2014)	✓	—	—	—	—	—	Abrol et al. (2020); Bass et al. (2020, 2022); Turkan & Tek (2021); Eitel et al. (2019a)
Meaningful Perturbation (Fong & Vedaldi, 2017)	✓	—	—	—	—	—	Kan et al. (2021); Uzunova et al. (2019)
Gradients (Simonyan et al., 2013)	—	✓	—	—	—	—	Lin et al. (2022a); Zeineldin et al. (2022); Thomas et al. (2022); Cui and Weng (2022); Oh et al. (2019)
Integrated Gradients (Sundararajan et al., 2017)	—	✓	—	—	—	—	Wang et al. (2023a); Manjunatha & Esfahani (2021); Lin et al. (2021a); Choi et al. (2022); Supekar et al. (2022a)
Gradient ⊙ Input (Shrikumar et al., 2016)	—	✓	—	—	—	—	Thomas et al. (2022); Eitel et al. (2019a); Dyrba et al. (2020a); Cui & Weng (2022); McClure et al. (2020)
Grad—CAM (Selvaraju et al., 2017)	—	✓	—	—	—	—	Chen et al. (2022d); Lin et al. (2022a); Zhang et al. (2021b); Yang et al. (2018); Bass et al. (2020)
SmoothGrad (Smilkov et al., 2017)	—	✓	—	—	—	—	Zeineldin et al. (2022); Thomas et al. (2022); Rahman et al. (2022); McClure et al. (2020); Wang et al. (2022)
DeepLIFT (Shrikumar et al., 2017)	—	—	✓	—	—	—	Prasad et al. (2022); Gupta et al. (2019, 2021a); Pianpanit et al. (2021); Bennett et al. (2021)
DeConvNet (Zeiler & Fergus, 2014)	—	—	✓	—	—	—	Dyrba et al. (2020a); Cui & Weng (2022); Zhao et al. (2017)
GBP (Springenberg et al., 2014)	—	—	✓	—	—	—	Bass et al. (2020); Zeineldin et al. (2022); Baumgartner et al. (2018); Eitel et al. (2019a); Dyrba et al. (2020a)
DTD (Montavon et al., 2017)	—	—	✓	—	—	—	Dyrba et al. (2020a); Nakagawa et al. (2020); Tinauer (2020)
LRP (Bach et al., 2015)	—	—	✓	—	—	—	Thomas et al. (2022); Korda et al. (2021); Hofmann et al. (2022); Eitel et al. (2019a); Thomas et al. (2019)
LIME (Ribeiro et al., 2016)	—	—	—	✓	—	—	Magesh et al. (2020); Lombardi et al. (2021); Saboo et al. (2022); Magboo et al. (2022a); Gaur et al. (2022)
SHAP (Lundberg & Lee, 2017)	—	—	—	✓	—	—	Lombardi et al. (2021); El-Sappagh et al. (2021); Turkan & Tek (2021); Chun et al. (2020); Ghosal et al. (2022)
Attention (Vaswani et al., 2017)	—	—	—	—	✓	—	Zhao et al. (2022); Jin et al. (2019); Fu et al. (2021); Jin et al. (2020); Yang et al. (2019)
Joint Training (Hendricks et al., 2016)	—	—	—	—	✓	—	Wen et al. (2022); Zhu et al. (2022); Bass et al. (2020, 2022); Wilms et al. (2021)
Model Transparency (Ba et al., 2014)	—	—	—	—	✓	—	Wood et al. (2019); Ravi et al. (2022); Qiu et al. (2020); Lee et al. (2019a); Mahmood et al. (2022)
—	—	—	—	—	—	✓	Oh et al. (2022b); Abrate & Bonchi (2021); Wilms et al. (2021, 2022); Oh et al. (2022a)

We note that the outputs of explanation approaches other than visual methods are often presented using suitable visualizations (distillation methods, attention score visualizations, explanatory example visualizations, etc.), but their explanation mechanisms are not inherently visual.

### 2.2 Visual explanation methods

Visual explanation methods generate explanation maps, also known as saliency maps or heatmaps. These methods directly highlight the influence or contribution of each feature to the model’s decisions. These attribution values indicate the magnitude and direction (if signed) of the sensitivity or importance values of the features. Generally, visual explanation methods for model interpretability fall into two main categories. The first category is *Backpropagation Methods*. *Backpropagation methods* are further classified into gradient backpropagation and modified backpropagation methods based on how backpropagation is performed during explanation computation. The second category of visual explanation methods is called *Perturbation-Based Methods*. Both backpropagation-based and perturbation-based methods are generally applied post hoc, meaning they are used after the model has been trained. To define the post hoc visual explanation methods, let *input* be a vector $\text{x}\in {\mathbb{R}}^{d}$, where d is the dimension of the input; let $F:{\mathbb{R}}^{d}\to {\mathbb{R}}^{C}$ denote the model function, where C is the number of classes; and let $F_{c}\left(\text{x}\right):{\mathbb{R}}^{d}\to \mathbb{R}$ denote the mapping that defines the class-specific logit, where c is the predicted class. An explanation method generates an *explanation map* $E:{\mathbb{R}}^{d}\to {\mathbb{R}}^{d}$ that maps x to an output of the same shape, highlighting the crucial decision regions. We note that heatmaps can be generated for non-classification model as well (e.g., regression), where we directly use the model function $F:{\mathbb{R}}^{d}\to \mathbb{R}$ to generate explanations instead of using the mapping $F_{c}$.

#### 2.2.1 Gradient backpropagation

In gradient backpropagation, the model output is backpropagated to the input to compute how the output changes with a small change in each feature dimension. The key assumption of gradient-based approaches is that the underlying model is differentiable. Different gradient-based XAI methods formulate explanations differently. We describe the major gradient-based methods as follows:

- **Gradients (GRAD)** (Baehrens et al., 2010; Simonyan et al., 2013) is the gradient of the class-specific logit with respect to input features x. Intuitively, it identifies the features where the least perturbations in the input will change the target output the most. This method identifies local sensitivities associated with the target category but does not necessarily explain a decision (Samek et al., 2016). While this method is computationally efficient, it suffers severely from the saturation effect, meaning that when the output of an activation is saturated, the gradient becomes zero and the corresponding features receive zero attributions, even though those features may have contributed meaningfully to the prediction.
- **Gradient ⊙ Input** (Shrikumar et al., 2016) is the element-wise multiplication, denoted by ⊙, of the gradients and the input. This method is often preferred over vanilla gradients because it reveals the direction and strength of the input features associated with the model decision. However, this approach may result in undesirable side effects. For example, final maps can be heavily dominated by the input regardless of its actual importance. Moreover, this method does not address the saturation problem, which can lead to incorrect attributions, or the thresholding artifact of raw gradients, which can cause sudden jumps in importance scores.
- **Integrated Gradients (IG)** (Sundararajan et al., 2017) satisfies several desirable properties that every attribution method should satisfy. Refer to Section 5.3 for the details of these properties and the post hoc methods that satisfy them. IG also addresses the saturation problem caused by computing gradients only at the current input value and not considering how the output changes over a range of inputs. Unlike raw gradients, IG uses interpolation technique to integrate importance at k different discrete intervals between uninformative baseline $\bar{\text{x}}$ and the input x, to give an integrated estimate of feature importance. While the choice of baselines is up to the users, Sundararajan et al. (2017) suggest a black image as a natural baseline choice for image recognition tasks, and a zero vector is a reasonable choice for text-based networks. However, the choice of baseline can lead to different interpretations of the results (Kindermans et al., 2019), and finding a suitable baseline across different domains is not easy. Moreover, this method incurs computational overhead because it requires computing gradients at multiple points between the current input and the baseline. Additionally, since IG is still based on gradients and employs a model-agnostic straight-line path, it inherits many of the issues associated with raw gradients, often leading to noisy attributions. An adaptive path-based method, *Guided Integrated Gradients* (Kapishnikov et al., 2021), has been shown to reduce noise in attributions for image models.
- **Smooth-Grad (SG)** (Smilkov et al., 2017) expresses a feature as an averaging of N noisy estimates obtained when input is perturbed with some Gaussian noise %. The main motivation behind this method is to reduce noise in gradient explanations by averaging gradients over noisy input samples. It can easily be combined with other gradient-based approaches. While this method tends to reduce visual noise, it does not provide any theoretical guarantees, may obscure sharp local features through averaging, incurs increased computational overhead, inherits the issues of the underlying method on which it is built, and is sensitive to both the number of noisy samples and the choice of noise parameters.
- **CAM and GRAD-CAM:** *Class Activation Map (CAM)* (Zhou et al., 2016) was proposed to visualize the focal regions using global average pooling on the last layer activations in convolutional neural networks (CNNs). Subsequently, Selvaraju et al. (2017) proposed a gradient-weighted CAM, called Grad-CAM, and generalized the CAM computation to a broader set of networks by leveraging the gradients of the last layer activation maps. Grad-CAM explanation is first upsampled to the input resolution using bilinear interpolation and then overlaid on the input image. Grad-CAM, which generates coarse-grained saliency maps, is sometimes combined with Guided backpropagation (GBP) for pixel-space visualization through an element-wise product called Guided Grad-CAM. However, Guided Grad-CAM inherits the limitations of GBP, which might generate misleading saliency maps due to the saturation effect as discussed earlier. Moreover, Grad-CAM and its variants are specific to Convolutional Neural Networks (CNNs).

#### 2.2.2 Modified backpropagation

The modified backpropagation category refers to the methods that use different forms of backpropagation other than standard backpropagation. All the methods in this category use some modification to the standard gradient backpropagation. For example, DeConvNet (Zeiler & Fergus, 2014) and Guided Backpropagation (GBP) (Springenberg et al., 2014) modify the standard gradient backpropagation for ReLU nonlinearity, whereas Layer-wise Relevance Propagation (LRP) (Bach et al., 2015) uses relevance conservation principle when redistributing the output score backward to the input features.

DeConvNet (Zeiler & Fergus, 2014) and Guided Backpropagation (GBP) (Springenberg et al., 2014) were proposed to address the ReLU nonlinearity issue in CNNs, which are particularly useful for spatial data (e.g., images). ReLU non-linearity is often preferred in CNNs because it does not exhibit a saturation effect as observed in sigmoid and tanh activation functions. However, unlike sigmoid or tanh functions, which are smooth and do not have a hard zero cut-off during backpropagation, ReLU introduces a gating effect during backpropagation. Specifically, in vanilla backpropagation, all gradients for positive activations at the ReLU non-linearity are allowed to pass through, even if they are noisy or uninformative, while all gradients for negative activations are suppressed, even if they might be informative. This behavior can result in noisy saliency maps. The key motivation behind DeConvNet and Guided Backpropagation was to suppress noise and produce sharper, more interpretable saliency maps (i.e., features that trigger activations of higher-layer neurons) by modifying the gradient flow through ReLU units.

- **DeConvNet** (Zeiler & Fergus, 2014) allows only positive gradients to pass through the ReLU non-linearity, thereby preserving gradient flow that contributes positively to the output activation and maintaining symmetry with the forward pass. Negative gradients are ignored, as they may represent noise or negative evidence for the prediction. Unlike standard gradients (Baehrens et al., 2010; Simonyan et al., 2013), which are often noisy and hard to interpret, DeConvNet attempts to invert the convolution operation during backward pass and thus produces sharper and interpretable visualization than raw gradients. DeConvNet relies on deconvolution and unpooling, which are specific to CNN architectures. So, DeConvNet is not suitable for other DL architectures, such as recurrent neural networks (RNNs) and transformers.
- **Guided Backpropagation (GBP)** (Springenberg et al., 2014) combines the ideas of both vanilla gradients and DeConvNet. The key motivation is that DeConvNet cannot identify sharp and recognizable image structures because it does not check which input patterns were responsible for positive activations at the ReLU non-linearity and considers only the upstream gradients during backpropagation. This can be misleading if a max-pooling layer—which ensures gradient flow through positions of maximum activations—is absent in a CNN model. Therefore, GBP imposes a rigid constraint on the ReLU non-linearity during backpropagation, ensuring that positive gradients are backpropagated only when the original ReLU outputs are positive. As a result, GBP can produce sharper and more interpretable saliency maps even in the absence of max-pooling layers. GBP is best suited for CNNs and spatial data.
- **Layer-wise Relevance Propagation (LRP)**: The key motivation of LRP (Bach et al., 2015) is to faithfully generate an explanation rather than producing sharper heatmaps as in DeConvNet and GBP. While gradient-based approaches measure the sensitivity of the output with respect to input features, LRP decomposes the output into feature relevance scores and conserves evidence for, and against, a network decision. The conservation principle requires that the sum of relevance scores of neurons in each layer equals the sum of relevance scores of the next layer. LRP is fast and the computational complexity is similar to a single gradient backpropagation. LRP uses the term “relevance” to refer to the relevance of a unit i in layer l. It starts at target neuron c in the last layer L and treats the target neuron’s activation as its relevance. The relevance of all other neurons in layer L is set to 0. During backward propagation, it computes attributions for neurons at other layers using one or more recursive rules (Montavon et al., 2019), such as LRP-0/%/γ/αβ , appropriate to the characteristics of the layers in a network. %, γ, αβ are enhancement terms, respectively, to improve stability, prioritize positive contributions, and separate positive and negative contributions over the basic rule LRP-0. The final attribution is the collection of all the relevances in the input layer.
- **DeepLIFT:** DeepLIFT (*Deep Learning Important FeaTures*) (Shrikumar et al., 2017) was proposed to address the limitations of gradient-based methods when dealing with activation functions such as ReLU, sigmoid, or tanh. For example, gradients of ReLU units are zero when they do not produce positive forward input but may still carry information toward the output (Shrikumar et al., 2016). Also, GBP and DeConvNet both discard negative gradients and can fail to highlight input areas that contribute negatively to the output. As discussed earlier, the gradient-based approaches fail to assign reliable importance due to the saturation effect. To resolve the issue, DeepLIFT assigns attributions by comparing a neuron’s activations using the original input x and baseline input $\bar{\text{x}}$. As DeepLIFT uses difference-from-reference, which is continuous, it avoids sudden increase in the importance score caused by discontinuous gradients. Different versions of DeepLIFT, such as Linear, Rescale, and RevealCancel, have their own propagation rules and ways of treating positive and negative attributions. DeepLIFT is computationally fast and an explanation can be generated in a single backward pass after a prediction.
- **Deep Taylor Decomposition:** In Deep Taylor Decomposition (DTD) (Montavon et al., 2017), the relevance of each neuron in the network can be decomposed through a Taylor expansion of its inputs based on a root (reference) point. These decompositions are then aggregated backward to form an explanation in the input space. The main difference between DTD and LRP is that DTD employs Taylor decomposition, whereas LRP propagates relevance based on heuristic rules while conserving relevance layer by layer. While DTD provides theoretical foundations for LRP, which does not require a root point, the main limitation of DTD is that it requires a reference point. In some cases, finding a suitable reference point can be expensive or ambiguous, and different choices of root may produce different heatmaps.

#### 2.2.3 Perturbation-based methods

In perturbation-based approaches, the attribution of features is computed by removing, masking, or modifying the features and evaluating the model output on the new input. The effect of this perturbation on the model output is considered as the importance or attribution value of a feature (or a set of features) (Ancona et al., 2017).

- **Occlusion Sensitivity** (Zeiler & Fergus, 2014) occludes different portions of the input with a gray square and expects a significant drop in classification score if the portion is strongly discriminative for the prediction. We note that there is a distinction between “gradient-based” sensitivity and “perturbation-based” sensitivity, even though the term “sensitivity” is sometimes used in both cases. In “gradient-based approaches,” the sensitivity of a feature is calculated based on the gradient of the model’s outcome (score) with respect to that feature. Conversely, in “perturbation-based approaches,” the sensitivity of a feature is measured by comparing the model’s outputs for the original input and a modified version of the input, usually through different forms of perturbations. The sensitivity in gradient-based approaches is often referred to as “gradients” or “gradient sensitivity.” *Occlusion Sensitivity* is computationally expensive because each perturbation requires a separate forward pass. Moreover, this method does not account for the co-occurrence of features, as it occludes only one region at a time. It is also susceptible to the saturation effect and may underestimate feature importance (Shrikumar et al., 2017). While this approach can be conceptually applied to any ML model, it has been used primarily in DL models for computer vision tasks.
- **Meaningful Perturbation** (Fong & Vedaldi, 2017) finds the smallest input region whose deletion impacts the output most. Although the method is conceptually model-agnostic, it requires access to gradient information. This method also enables the joint inclusion or exclusion of different image regions for accurate measurement of the perturbation effect on the model’s output. This method learns an interpretable minimal perturbation mask that significantly reduces the classification score. To compute the mask, this method uses the gradient descent method and progressively accumulates information through backpropagation. This method is computationally expensive, and the generated explanations are highly sensitive to hyperparameter choices; poor choices can result in meaningless explanations.

**Fig. 2.**
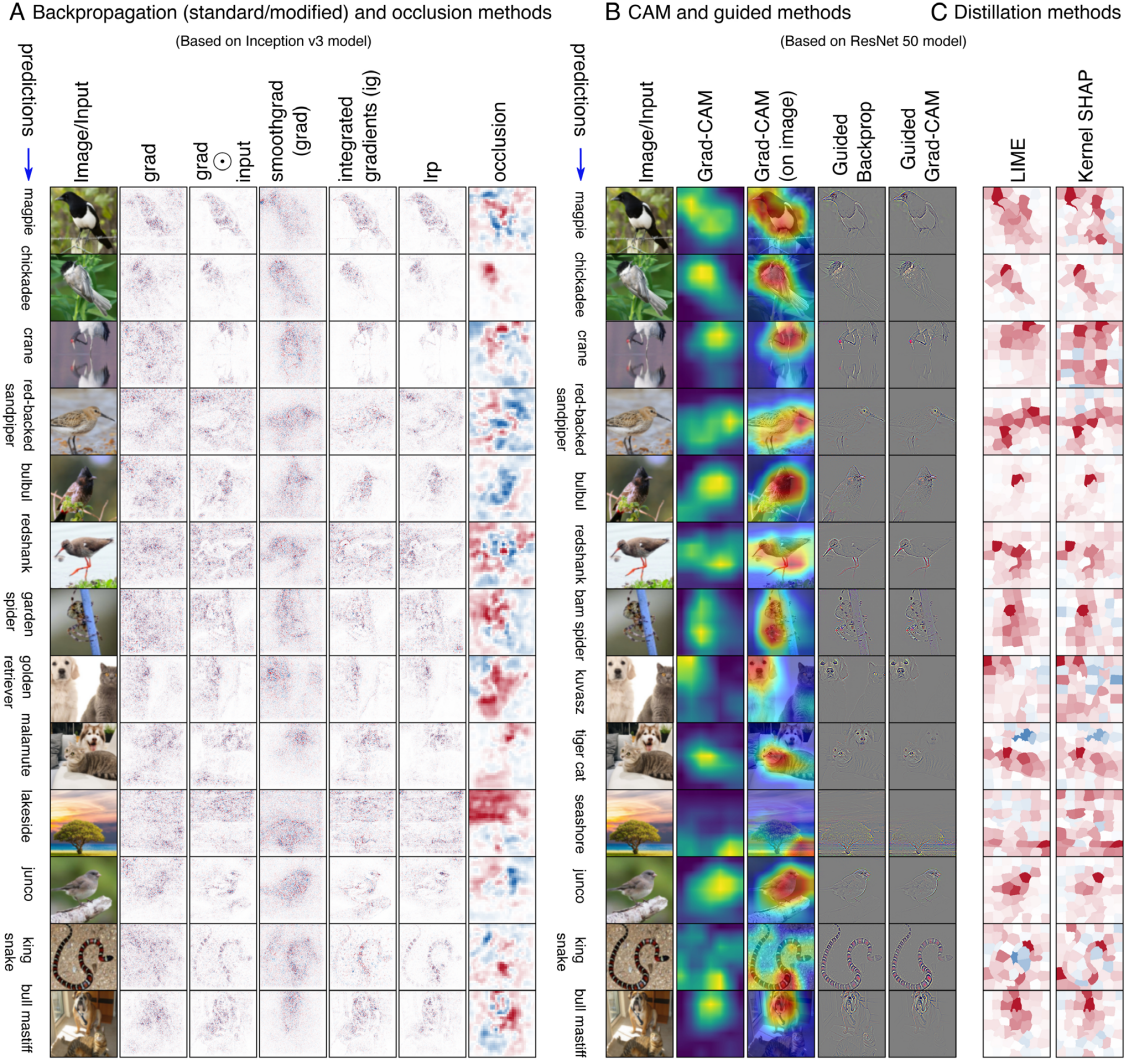
Explanations generated using all major post hoc interpretability methods. (A) *Backpropagation* and *Occlusion Methods*. (B) *CAM* and *Guided Methods*. (C) *Distillation Methods*. Feature mask was generated using simple linear iterative clustering (SLIC) of scikit-image. We used 1000 iterations to build the interpretable model.

Refer to *Panel A* and *Panel B* of Figure 2 to see a few explanation samples generated using *visualization* methods.

In addition to the above methods, to understand how convolutional networks form concepts from the training data, researchers have also attempted other feature visualization approaches, such as visualizing feature maps directly in the convolutional layers or visualizing features in the input that activate certain neurons or layers in the DL model (Erhan et al., 2009; Olah et al., 2017).

### 2.3 Distillation methods

In distillation methods, the goal is to extract the important aspects of the model to be explained by mimicking the behavior of the underlying model. The model to be explained is considered a “black box.” To explain the black box, a separate, directly interpretable (linear model, decision trees, etc.) surrogate model is created to approximate the input–output behavior of the original model. This surrogate model is model-agnostic and requires no internal information from the original model. This class of surrogate models can be used to generate both local and global explanations.

- **LIME** (Ribeiro et al., 2016), also called *Local Interpretable Model-agnostic Explanations*, is based on a surrogate model, typically linear. The key motivation of LIME is to explain any black-box model using an interpretable model. LIME takes a sample, divides the input into multiple simplified regions, perturbs it multiple times based on random binary vectors representing the presence and absence of those regions, and computes output scores in the original model. These binary vectors are subsequently employed to train an interpretable surrogate model that generates identical outputs. The coefficients within the trained surrogate model correspond to the attribution of each input region in the input space. LIME is computationally expensive because it perturbs the input sample multiple times. Moreover, because LIME fits an interpretable model locally around a sample, it fails to provide a global understanding of the model. Also, the assumptions that the interpretable model is linear and that features are perturbed independently may potentially produce erroneous explanations.
- **SHAP:** Shapley values, initially introduced in cooperative game theory by Shapley (1953), are used to determine the marginal contributions of individual players. It involves considering the outcomes of all possible coalitions in the game to calculate the marginal contribution of each player i in a scenario where all players participate. Lundberg and Lee (2017) developed SHapley Additive exPlanations (SHAP), which measure Shapley values of a conditional expectation function of the main model. SHAP also satisfies several desirable theoretical properties—such as local accuracy, missingness, and consistency—all of which guarantee the faithfulness of an explanation to some degree, whereas LIME lacks these formal guarantees. As SHAP is computationally expensive, an approximation method, known as Kernel SHAP, was proposed. Kernel SHAP is a regression-based, model-agnostic, and computationally efficient estimation of Shapley values. They demonstrated that Shapley values are the unique solution for additive feature attribution methods with distinct properties. Since linear LIME is an additive feature attribution method, the authors proposed a suitable loss function, weighting kernel, and regularization term to recover Shapley values using the LIME formulation. The same study also proposed Deep SHAP for DL models, which offers a computational advantage over Kernel SHAP by leveraging the compositional structure of DL models. See Section 5.3 for a discussion of other issues of LIME and SHAP.

Refer to *Panel C* of Figure 2 to see a few examples of explanations generated by distillation methods.

XAI methods that produce saliency maps generally fall under three categories (Kindermans et al., 2017, 2019). Gradients capture the sensitivity of the model output to small perturbations in the input, hence referred to as a *Sensitivity Method*. The sensitivity of each feature is considered as their importance for the model’s decision. Some gradient-based methods, such as DeConvNet and Guided Backpropagation (GBP), are called *Signal Methods* because these methods isolate input patterns that activate neuron activations in higher layers. Methods such as IG, LRP, DeepLIFT, and DTD are called *Attribution Methods*, which decompose the model output into individual feature contributions. The sum of these contributions should equal the target output score. LIME and SHAP can also be treated as additive feature attribution methods (Lundberg & Lee, 2017).

### 2.4 Intrinsic methods

Intrinsic methods focus on incorporating interpretation as part of the model design or training rather than doing a separate post hoc analysis. These methods are model specific and are usually implemented based on different design or training perspectives. Compared with post hoc methods, intrinsic approaches are generally more desirable because they are tightly integrated with the model’s training and outputs, and are, therefore, expected to provide more faithful explanations. In contrast, post hoc methods rely on separate strategies that may not be directly aligned with the model’s inner workings. While some shallow models, such as linear models and decision trees, are inherently interpretable, developing intrinsic interpretability methods for DL models requires specialized knowledge of both the application domain and DL itself, as it addresses a more complex problem. There are many ways of obtaining intrinsic interpretability in DL, such as *attention mechanism*, *joint training*, and *modular transparency*. In this section, we briefly discuss some of the common practices used to obtain intrinsic interpretability in DL.

#### 2.4.1 Attention mechanism

An *attention mechanism* is a technique in DL models that computes the conditional distribution over inputs leading to a vector of weights that specify the importance of different regions in the input for the given context. Several approaches exist for computing attention weights, such as using additional fully connected layers (Bahdanau et al., 2014) or employing scaled dot-product attention (Vaswani et al., 2017). For detailed explanations, see the respective studies. Attention mechanisms have been shown to improve DL model performance (Vaswani et al., 2017), and, if carefully designed, attention weights can be visualized as heatmaps to provide easy-to-understand explanations. While several attention mechanisms have been proposed in the literature, they can be further tailored to the underlying hypothesis and specific task. For example, Jetley et al. (2018) used a trainable attention module to highlight relevant areas of an input image while suppressing other irrelevant regions, all integrated into an end-to-end model training process.

#### 2.4.2 Joint training

*Joint training* is the concept of training a model simultaneously for performance and explanations (Hendricks et al., 2016; Liu et al., 2019b; Zellers et al., 2019). Joint training requires a complex objective function that optimizes both the original task and an additional explanation task. The additional task may provide a direct textual explanation, generate an explanation association between inputs or latent features with human-understandable concepts, or learn semantically meaningful model prototypes (Ras et al., 2022). In its simplest form, the objective function consists of two loss components: one for the prediction task and another for the explanation task. For example, Hendricks et al. (2016) proposed a visual explanation model that generates textual explanations describing image content relevant to a particular class, using *discriminative* and *relevance* loss functions. While such explanations are highly desirable, joint training introduces additional design challenges, and appropriate labeled datasets for explanation generation may not always be available.

#### 2.4.3 Modular transparency

*Modular transparency* (Thibeau-Sutre et al., 2023) refers to a network consisting of multiple modules. The modules have pre-specified design goals and are usually black boxes. However, the interactions among the modules are transparent, allowing explanations to be derived based on a global understanding of how the model functions (Ba et al., 2014). There are no fixed design rules for modular transparency, that is, its implementation is largely intuitive. For example, Ba et al. (2014) proposed a modular deep learning model combining attention mechanisms and reinforcement learning for multiple object recognition tasks. The model was inspired by human visual sequence recognition: it moves sequentially to relevant locations, recognizes individual objects, and updates its internal representation. Explanations are obtained by analyzing both individual modules and their interactions. As the form of explanations is not fixed for modular transparency, generating meaningful explanations may require significant effort, and it may also require support from other post hoc XAI methods.

### 2.5 Counterfactual explanations

Counterfactual explanations (Dandl et al., 2020; Karimi et al., 2020; Mothilal et al., 2020; Wachter et al., 2017), by definition, describe hypothetical scenarios. Instead of directly explaining the current prediction, they identify the minimal change needed to alter the model’s outcome. This type of explanation is considered human friendly (Molnar, 2020) because it provides actionable insights that help users better understand the current outcome and potentially influence future outcomes. Counterfactual explanations are typically generated by solving either a single-objective (Wachter et al., 2017) or a multi-objective (Dandl et al., 2020) optimization problem. For example, Wachter et al. (2017) proposed a single-objective formulation that minimizes the distance between the actual sample and its modified counterpart (i.e., ensuring they remain similar), while keeping the model’s prediction for the modified sample as close as possible to a predefined (desired) outcome. These explanations can be generated in both model-specific and model-agnostic settings. The latter just requires access to the prediction function (Molnar, 2020). In neuroimaging, counterfactual explanations can highlight critical biomarkers, helping researchers generate new hypotheses about disease progression mechanisms and potentially contributing to drug discovery. When biologically meaningful, counterfactuals may also assist clinicians in prioritizing personalized treatments. However, the main limitation of this approach is the challenge of generating realistic counterfactuals. It can be computationally expensive for high-dimensional data and may produce implausible recommendations. Furthermore, multiple counterfactuals may coexist, which may further complicate interpretation. This method is also less effective for providing insights into the overall function of the model, as it focuses on individual data points. Finally, counterfactual-based insights require extensive clinical validation before drawing any meaningful conclusions.

### 2.6 Example-based explanations

Example-based approaches aim to identify the representative training samples that played a role in the prediction of a test sample. Although this class of explanations is still evolving, several studies (Basu et al., 2020; Feldman & Zhang, 2020; Ghorbani & Zou, 2019; Hampel et al., 1986; Han et al., 2020; Ilyas et al., 2022; Jia et al., 2019; Koh & Liang, 2017; Zha & Shen, 2020) have proposed methods to determine the training points that influenced a specific test case. For instance, Koh and Liang (2017) used gradients and Hessian-vector to compute first-order “influence function” approximation to identify the training points responsible for a given prediction. Ilyas et al. (2022) used a parametric linear function to predict the outcome of a neural network when trained on a subset of the given training set and evaluated on a specific but arbitrary target example. This parametric characterization of models gives more insights into the model behavior. Chen et al. (2019a) proposed prototype-based explanations of model decisions. The model dissects a test image to find some prototypical parts that correspond to the prototypes the model learned and finally combines evidence based on similarity scores between parts and the prototypes to reach the final decision. Each prototype represents a learnable prototypical activation pattern of the convolution output and corresponds to a prototypical training image patch. Each prototypical part in the test image is matched with only one training patch example, and thus this approach is referred to as “this looks like that.” Matching with only one prototypical training image patch may limit the notion of the underlying concept the model learned. Recently, Ma et al. (2024) modified the architecture of the previous approach and proposed a new approach called “this looks like those,” requiring that the model learns prototypical concepts from multiple training image patches increasing the reliability and explainability of the learned concepts.

### 2.7 Concept-based explanations

Kim et al. (2018) proposed a human-friendly interpretability approach called *Testing with Concept Activation Vectors (TCAV)*. This approach utilizes directional derivatives to provide a linear interpretation of a model’s prediction in relation to user-provided concepts. Precisely, TCAV checks if the internal representations of the model are distinguishable when such concepts are present or absent. A more generalized version of concept-based explanations, called concept activation regions (Crabbé & van der Schaar, 2022a), is proposed later to overcome the linear separability condition for concepts. TCAV was used in a diabetic retinopathy (DR) prediction problem. Specifically, TCAV was used to test the importance of different diagnostic concepts, such as the presence of microaneurysms and pan-retinal laser scars in the retina, which physicians generally use in their diagnoses. For some DR levels, TCAV was able to identify the correct diagnostic concepts as being important to the model. However, incorrectly identified concepts provide a means to fix model errors. Concept-based explanations are beneficial because, in many domains, individual feature importance (e.g., importance of pixels) alone may not carry significant insights (Imrie et al., 2023). However, this idea of concept-based explanations has not been extensively explored in the field of medical imaging (Graziani et al., 2020), potentially due to the need for users to provide sets of examples as concepts. We note that this review did not find any neuroimaging applications employing “example-based” or “concept-based” explanations.

We note that the XAI methods that do not need separate post hoc explainability stage are called “model-based” methods (Murdoch et al., 2019; Van der Velden et al., 2022) as in the case of “inherently interpretable” and “inherently explainable” models (achieved through intrinsic methods). While all “model-based” methods are, by nature, “model-specific,” post hoc explanation methods can be grouped into “model-specific” and “model-agnostic” categories. Model-specific XAI approaches are only applicable to certain class of models, whereas model-agnostic approaches are independent of the models to be explained. For example, backpropagation-based methods are only applicable to the class of models where the output can be backpropagated either by using gradients or by using relevance propagation rules and hence are “model-specific.” On the contrary, XAI methods leveraging interpretable surrogate models (e.g., LIME (Ribeiro et al., 2016) and Kernel SHAP (Lundberg & Lee, 2017)) or methods that do not rely on the internals of the model except outputs (e.g., occlusion sensitivity (Zeiler & Fergus, 2014) and meaningful perturbations (Fong & Vedaldi, 2017)) to explain the original black boxes are model-agnostic. For a detailed analysis of the terminologies, recently used XAI methods, data modalities, and interpretability scopes in medical imaging tasks, we refer to the scoping review by Champendal et al. (2023).

## 3 Evaluation Approaches

To evaluate XAI methods quantitatively, numerous tests and evaluation metrics have been proposed in the literature (Alangari et al., 2023). However, these techniques often fail to verify whether the ML model under test is operating in shortcut mode—that is, relying on spurious associations between inputs and outcomes. Detecting and addressing such issues are typically the responsibility of the model developers. If they are not resolved before evaluation, the results produced by these metrics may be misleading. In the following, we discuss some commonly used sanity checks and evaluation metrics.

### 3.1 Sanity checks

Model explanation methods should be sensitive to model parameters and accurately reflect the relationship between data and labels. To ensure the rationale behavior of these methods, Adebayo et al. (2018) proposed the following sanity checks:

**Model Parameter Randomization Test (MPRT):** Explanations should be influenced by the parameters of the model. By randomizing the model parameters, either fully or in a cascading manner, the generated explanations should differ from those of the originally trained model. There are two variants of MPRT, called smooth MPRT (sMPRT) and efficient MPRT (eMPRT), proposed by Hedström et al. (2024). sMPRT intends to denoise the explanations before computing the similarity measure by averaging over N noisy input samples, similar to SmoothGrad (Smilkov et al., 2017), at each randomization step. eMPRT removes the need for layer-by-layer randomization and estimates the faithfulness of the explanation method by evaluating using histogram entropy if the explanation complexity rises after randomization.

**Data Randomization Test:** To test the effectiveness of an explanation method, the training labels are permuted, disrupting the relationship between the data and labels. This test assumes that a model trained on this shuffled data, which essentially memorizes the labels, should generate explanations that differ significantly from those of the model trained on the original data. However, this test is time consuming as training a model on randomized data requires a longer time and customized hyperparameters to achieve convergence.

**Random Logit Test:** This metric evaluates the difference in explanations between the ground truth and a random logit, expecting a change in explanations (Sixt et al., 2020). It is inherently designed for classification models but could be adapted, with modifications, for multi-output regression or other vector-valued prediction tasks.

### 3.2 Evaluation metrics

Human evaluation of explanation methods can be inaccurate because adversarial samples can be created to deceive humans while changing model predictions (Goodfellow et al., 2014; Szegedy et al., 2013). To assess interpretation methods quantitatively, specific properties need to be formally defined for the domain. Additionally, appropriate metrics are required to evaluate the behavior of an interpretation method. Generally, when the generated attributions are not convincingly meaningful, it is difficult to determine whether the issue lies with the model or the interpretation method. Well-designed evaluation metrics help disentangle these issues and provide a more objective basis for comparing explanation methods. This section introduces evaluation metrics suggested in the interpretability literature.

#### 3.2.1 Metrics for ground-truth datasets

Arras et al. (2022) proposed two evaluation metrics that can reliably quantify explanation methods using a synthetic dataset with pixel-level ground-truth masks for the Visual Question Answering (VQA) task. This dataset contains 3D objects with various illumination, color, material, size, and shape, along with associated questions suitable for the VQA task.

**Relevance Mass Accuracy:** This metric calculates the proportion of total attributions that reside within the ground-truth mask.

$$\text{Relevance Mass Accuracy}=\frac{{\text{R}}_{\text{within}}}{{\text{R}}_{\text{total}}}\;\;\text{with }{\text{R}}_{\text{within}}=\sum_{\begin{array}{c} k=1\\ s.t.p_{k}\in GT \end{array}}^{\left|GT\right|}r_{p_{k}}\text{and}\;{\text{R}}_{\text{total}}=\sum_{k=1}^{N}r_{p_{k}},$$ (1)

where $r_{p_{k}}$ is the relevance score for the pixel $p_{k}$. The *relevance* of a pixel refers to the attribution or importance value assigned to that pixel by an explanation method. N is the total number of pixels. GT is the set of all pixels within the ground-truth area.

Relevance Rank Accuracy: Let K be the number of pixels within the ground-truth masks. This metric measures how many high-ranked K pixels are within the relevance area. Let ${\text{P}}_{\text{top K}}=\{p_{1},p_{2},\ldots ,p_{K}|r_{p_{1}}\text{​}>r_{p_{2}}\text{​}>r_{p_{3}}\cdots >r_{p_{K}}\}$ be the top K pixels sorted in descending order of their attribution values. *Rank Accuracy* is defined as follows:

$$\text{Relevance Rank Accuracy}=\frac{\left|{\text{P}}_{\text{top K}}\cap GT\right|}{\left|GT\right|}.$$ (2)

The argument GT refers to the set of pixels within the ground-truth region. These metrics can be adapted to the neuroimaging domain, but this would require a synthetic, task-specific dataset with ground-truth informative regions. However, a key caveat is that the original metrics were applied to a synthetic dataset, with associated questions and ground-truth masks that are oversimplified, which may not align with the complexity and variability of real-world neuroimaging data.

#### 3.2.2 Metrics for real-world datasets

Several measures have been proposed to assess the quality of explanations in real-world data experiments, including Infidelity (INF) (Yeh et al., 2019), Faithfulness Estimate (FE) (Alvarez Melis & Jaakkola, 2018), Monotonicity (M) (Arya et al., 2019), Sensitivity (SEN) (Yeh et al., 2019), Remove And Retrain (ROAR) (Hooker et al., 2019), Retain and Retrain (RAR) (Rahman et al., 2022), log-odds score (Shrikumar et al., 2017), Area Over MoRF Perturbation Curve (Samek et al., 2016), Smallest Sufficient Regions (SSR) (Dabkowski & Gal, 2017), RemOve And Debias (ROAD) (Rong et al., 2022), Accuracy Information Curves, and Softmax Information Curves (Kapishnikov et al., 2019).

**Infidelity (INF):** Infidelity (Yeh et al., 2019) quantifies the extent to which the model’s output changes in response to significant perturbations of the input. This metric calculates the expected mean square error between (1) the explanation multiplied by input perturbation and (2) changes in model outputs after perturbation. The explanation that has the least fidelity possible is optimal.

**Faithfulness Estimate (FE):** This faithfulness metric (Alvarez Melis & Jaakkola, 2018) obscures features, measures the probability drops, and computes the correlation between probability drops and attribution scores at various points. This metric indeed shows whether the estimated importance scores are empirically “true” importance or not. The higher the correlation, the better the explanation’s faithfulness. Unlike occlusion sensitivity, which generates explanations using perturbations, FE evaluates explanations using perturbations. Specifically, FE tests the alignment between perturbation effects and the estimated importance scores of the inputs.

**Monotonicity (M):** The monotonicity metric (Arya et al., 2019) tests whether the increase in class probability is monotonic when we incrementally add features in order of importance.

**Sensitivity (SEN):** Sensitivity (Yeh et al., 2019) quantifies the extent an explanation changes if the input is varied with small perturbations. It is generally implemented using Monte Carlo sampling-based approximation. Lower sensitivity implies that the explanations are less affected by insignificant perturbations of the test point. While lowering sensitivity is desirable, it is also important to note which parts of the input are perturbed.

**Remove and Retrain (ROAR):** ROAR metric (Hooker et al., 2019) modifies samples based on post hoc explanations by removing features that receive significant attributions. The model is then trained on the modified data, and a sharp drop in performance is expected due to the absence of important discriminative features.

**Retain and Retrain (RAR):** The ROAR approach modifies the dataset by removing the features that received the highest attribution values from each sample. In practice, if the training data have sufficient redundant discriminative features (Sturmfels et al., 2020), even after removing a significant number of features, the performance of the retrained model does not noticeably drop. In that scenario, ROAR fails to assess feature relevance accurately. That is, ROAR may produce erroneous evaluations when correlated features exist since capturing only a subset of correlated features is enough for correct prediction (Sturmfels et al., 2020). The Retain and Retrain (RAR) method, proposed by Rahman et al. (2022), resolves this issue by retaining only the critical features instead of removing them. However, both the ROAR and RAR methods are time consuming as they require full model retraining.

**Log-odds score:** The log-odds score metric (Shrikumar et al., 2017) identifies the main contributing pixels to convert an original prediction $c_{0}$ to a target prediction $c_{t}$ by removing 20% of the image based on descending ranking of $S_{c_{0}}\text{​}-S_{c_{t}}$. The change in log-odds score between $c_{0}$ and $c_{t}$ is then measured for the original image and the image with pixels removed to obtain the prediction $c_{t}$. A greater change in log-odds score indicates the greater significance of the removed pixels for the original class, making it useful for images with a strong structural association like MNIST, but not for natural images.

**Area Over MoRF/LeRF Perturbation Curve:** This metric, proposed by Samek et al. (2016), evaluates heatmaps based on how quickly the probability score drops when perturbing the most relevant regions. They create an ordered set $O=\left({\text{r}}_{1},\;{\text{r}}_{2},\ldots ,{\text{r}}_{L}\right)$ of pixel importance scores assigned by an interpretability method. This metric follows a process called MoRF (most relevant first), where a small rectangular region m×m surrounding each important pixel location ${\text{r}}_{p}$ is gradually removed. The quantity of interest is abbreviated as AOPC.

$$\text{AOPC}=\frac{1}{L+1}{\langle \sum_{k=0}^{L}f\left({\text{x}}_{\text{MoRF}}^{\left(0\right)}\right)-f\left({\text{x}}_{\text{MoRF}}^{\left(k\right)}\right)\rangle }_{p\left(x\right)}$$ (3)

${\text{x}}_{\text{MoRF}}^{\left(0\right)}=\text{x}$ and ${\text{x}}_{\text{MoRF}}^{\left(k\right)}$ is the perturbed image obtained when pixels at locations ${\text{r}}_{1},{\text{r}}_{2},\ldots ,{\text{r}}_{k}$ are gradually removed. ${\langle .\rangle }_{p\left(\text{x}\right)}$ represents averaging over all dataset samples. If the ranking strongly relates to the class label, removing it will lead to a more significant decrease in the functional value, resulting in a larger AOPC. An alternative to the MoRF metric is called least relevant first (LeRF). LeRF computes a similar score, but the removal of pixels (perturbation) starts with the least important pixels in the input and then measures how the trained model responds to calculate AOPC. Another variant of these metrics is called “area between the perturbation curves,” abbreviated as ABPC (Li et al., 2023a), which measures the difference between LeRF and MoRF (a higher score implies better explanations).

**Deletion (DEL) and Insertion (INS):** The *Deletion* metric (Petsiuk et al., 2018) gradually removes the important pixels and tests how quickly the model’s confidence drops for the predicted class. The *Insertion* metric (Petsiuk et al., 2018), however, incrementally adds important pixels according to the explanations and measures the rise of the probability scores for the class of interest. Both metrics measure the Area Under Curve (AUC), where lower and higher AUC are indicators of good explanations, respectively, for the *Deletion* and *Insertion* metrics.

**Smallest Sufficient Regions (SSR):** The SSR metric (Dabkowski & Gal, 2017) aims to determine the smallest region within an image that is sufficient for accurate prediction. This metric ensures that the classification remains the same while identifying the smallest possible area. The formal definition of this metric is as follows:

s(a,p)=log(ã)−log(p), (4)

ã=max(a,0.05), where a is the proportion of the cropped image to the original image, p is the probability of the object class when the classifier uses the cropped and resized image. A lower s(a,p) indicates a better saliency detector as it aligns with the idea of SSR: smaller area, higher probability score. However, this metric may not be suitable if the model is affected by the object’s scale and aspect ratio. Additionally, it penalizes saliency maps that are sparsely distributed, even with the same number of pixels, which is contrary to human perception. Moreover, masking creates a distinct boundary between the masked and salient region and may cause an out-of-distribution problem.

**RemOve And Debias (ROAD):** ROAD evaluation metric (Rong et al., 2022) addresses the high computational cost of retraining for evaluating attribution methods. The authors found that existing evaluations using MoRF or LeRF removal strategies are inconsistent in ranking the methods due to class information leakage through the shape of removed pixels. To overcome this inconsistency, the authors proposed a Noisy Linear Imputation operator that debiases the masking effect without requiring retraining.

**Performance Information Curve (PIC):** PIC (Kapishnikov et al., 2019) is a perturbation-based evaluation metric used to assess the appropriateness of an attribution method. The PIC evaluation involves creating a saliency-focused image by starting with a blurred image and combining it with a thresholded saliency mask. The resulting saliency-focused image is then fed into the model to evaluate the attribution performance. The accuracy/softmax score of the model is mapped as a function of the calculated entropy, which serves as a proxy measure of the re-introduced information content. The size of the compressed image is used to normalize the entropy, taking into account the proportion of entropy from the original image. The PIC is generated by aggregating the performance measurements over all information levels and samples in the dataset. There are two variants of the PIC: Accuracy Information Curve (AIC) and Softmax Information Curve (SIC).

**Pointing Game (PG):** *Pointing Game* (Zhang et al., 2018) is a localization metric that evaluates the localization ability of an explanation. This evaluation requires a bounding box and checks the extent to which the bounding box is aligned with the explanation.

**Consensus Score (CS):** The *Consensus Score* (Li et al., 2023b), also called *Pseudo Score* (Li et al., 2023a), first creates a pseudo ground truth, called consensus, by aggregating explanations from multiple trained models. Then, an explanation is evaluated based on its similarity score to the consensus.

**Synthetic Score (SynScore):** This metric (Li et al., 2023a) creates a synthetic ground-truth explanation by adding a trigger pattern to the input that causes model misclassification (Lin et al., 2021b). The idea is that a good XAI method should better match the trigger pattern for the misclassification.

**Modality Specific Feature Importance (MSFI)** (Jin et al., 2022) addresses two clinical requirements for evaluating explanations: the fidelity of the explanation to the model’s design process and the plausibility of human evaluation in relation to the model’s decision quality. This metric focuses on the modality importance (MI) and modality-dependent features in multi-modal medical imaging tasks. Traditional heatmap algorithms, typically used to depict the decision-making process of AI models, were found to be unable to meet the clinically relevant requirements when evaluated using the MSFI metric in a brain tumor classification task based on multimodal MRI.

### 3.3 Software toolkits for XAI

To find available XAI toolkits relevant to any domain, including some of those developed specifically for neuroimaging studies, we list some helpful resources in Table 2. The toolkits listed provide demonstration of the use of different modalities, including Graph (G), Image (I), Tabular or Structured (S), Text (X), and Time-series (T). To clarify, a “graph” in this context refers to a data structure used to represent neuroimaging data in graph-based models. Precisely, a graph consists of vertices representing brain regions and edges representing the connections between these regions. These connections can be based on anatomical measurements or functional correlations, and generally reflect the closeness or strength of the relationships. Vertices and edges are typically determined using structural and functional connectivity matrices. These resources assist researchers in generating explanations using various methods. While some tools (Agarwal et al., 2022; Hedström et al., 2023b) facilitate detailed quantitative assessment of explanation quality, addressing aspects such as faithfulness, robustness, randomization, complexity, localization, and fairness, most toolkits focus solely on explanation generation, allowing for only qualitative evaluation. We note that this list of toolkits is not exhaustive. We only selected some promising tools that have gained recognition based on GitHub repository activity, such as regular maintenance, the attention received by other researchers, the addition of new modalities, and evaluation metrics. For more information on the currently available XAI resources and evaluation toolkits, we suggest referring to the comprehensive survey by Le et al. (2023).

**Table 2.** Summary of the major resources that are available for reliably leveraging XAI in research.

		Demonstrated modalities							
Toolkit	Services	I	S	X	T	G	Code & instructions	Objective evaluation support?	DL/ML framework support
iNNvestigate (Alber et al., 2019)	A common interface to a variety of explanation methods	✓	✗	✓	✗	✗	v2.1.2	Not available	TF
InterpretML (Nori et al., 2019)	Interpretable glassbox and explainable black-box systems	✗	✓	✗	✗	✗	v0.7.2	Not available	SKL
AIX360 (Arya et al., 2019)	8 diverse explainability methods	✓	✓	✓	✓	✗	v0.3.0	FE, M	TF, PT, SKL
Captum (Kokhlikyan et al., 2020)	Gradient and perturbation methods with tutorials	✓	✓	✓	✗	✗	v0.8.0	INF, SEN	PT
Zennit (Anders et al., 2021)	LRP and rule-based approaches	✓	✗	✗	✗	✗	v1.0.0	Not available	PT
tf-explain (Meudec, 2021)	Gradient and occlusion-based approaches	✓	✗	✗	✗	✗	v0.3.1	Not available	TF
Interpretability Suite (Imrie et al., 2023)	Many supervised & unsupervised approaches, and selection criteria	✓	✗	✗	✓	✗	Github URL	Not available	PT, SKL
Quantus (Hedström et al., 2023b)	Gradient and perturbation methods with tutorials	✓	✓	✗	✓	✗	v0.6.0	30+ metrics but no fairness metric	PT, TF ^*^
MetaQuantus (Hedström et al., 2023a)	Evaluate evaluation metrics tutorials available	✓	✓	✗	✓	✗	v0.0.5	Finds reliable metric	PT
OpenXAI (Agarwal et al., 2022)	Datasets, pre-trained models, 7 feature attributions	✗	✓	✗	✗	✗	Github URL	22 metrics	PT
InterpretDL (Li et al., 2022c)	16 explanation algorithms tutorials, and showcases	✓	✗	✓	✗	✗	v0.8.0	MoRF/LeRF, INF, DEL/INS, PG	PD
M^4^ (Li et al., 2023a)	Supports 6 methods 5 metrics, 9 models	✓	✗	✓	✗	✗	Github URL	MoRF/ABPC, INF, CS, SynScore	PD +
GraphXAI Agarwal et al. (2023)	Graph data generation, 8 GNN explainability methods	✗	✗	✗	✗	✓	Github URL	Accuracy, Faithfulness Stability, Fairness	PT
NeuroXAI Zeineldin et al. (2022)	7 explanation algorithms, MRI classification, and segmentation	✓	✗	✗	✗	✗	Github URL	Not available	TF
Multimodal Explanation Jin et al. (2023)	Clinical guidelines and evaluation of 16 algorithms	✓	✗	✗	✗	✗	Github URL	MSFI, MI correlation ∆AUPC, etc. for MRI	PT

We provide a list of libraries, tools, frameworks, and code repositories for the implementation of post hoc explanation generation and evaluation. We report the modalities for which usage has been demonstrated by the toolkit authors: Graph (G), Image (I), Tabular or Structured (S), Text (X), Time-series (T). INF = Infidelity, SEN = Sensitivity, FE = Faithfulness Estimate, M = Monotonicity, CS = Consensus Score, SynScore = Synthetic Score, DEL = Deletion, INS = Insertion, PG = Pointing Game, LeRF = Least Relevant First, MoRF = Most Relevant First. Repositories last visited: August 18, 2025.

TF: TensorFlow

SKL: scikit-learn

PT: PyTorch

PD: PaddlePaddle Ma et al. (2019)

* : supports for captum, tf-explain, zennit

+ : based on InterpretDL, HuggingFace/PyTorch models

## 4 Deep Learning Interpretability in Neuroimaging

In this section, we provide a detailed discussion of various neuroimaging studies that have effectively used interpretable DL techniques. We categorize the papers based on the XAI methods employed. We have selected representative studies from different method categories for thorough discussion and illustration. Table 3 summarizes the selected papers for in-depth discussion for all method categories. While almost all studies refer to earlier studies for establishing consistency of their own findings, studies relying only on the literature for validation are described as “previous reports only” in the last column of Table 3. During our detailed discussion, we explore the various contexts in which interpretability has been applied and present the resulting findings. We provide summaries of other reviewed papers in each category in tables. Firstly, we present our reviews on post hoc methods.

**Table 3.** Summary of a detailed review of representative neuroimaging studies that use various XAI methods.

Authors, year	Study objective	Dataset	Modality	Interpretability	Explanation validation
**Miscellaneous Classification/Prediction Tasks**					
Yan et al. (2017)	SZ Classification	Chinese	rsFMRI	LRP	Previous reports only
Yang et al. (2018)	AD Classification	ADNI	sMRI	Occlusion, SA-3DUCM 3D-CAM, 3D-GRAD-CAM	Previous reports only
Rieke et al. (2018)	AD Classification	ADNI	sMRI	Gradients, Guided Backprop Occlusion, Brain Area Occlusion	AAL atlas Euclidean distance
Thomas et al. (2019)	Cognitive State Prediction	HCP	rsfMRI	ϵ-LRP	$\text{Meta}-{\text{analysis}}^{\upsilon^{0}}$
Eitel et al. (2019b)	${\text{MS Classification}}^{s^{2}}$	${\text{ADNI and VIMS}}^{d^{2}}$	sMRI	ϵ-LRP	Previous reports only
Böhle et al. (2019)	AD Classification	ADNI	sMRI	LRP-β	Scalable atlas Bakker et al. (2015)
Oh et al. (2019)	${\text{AD Classification}}^{s^{\text{9 }}}$	ADNI	sMRI	Gradients, Occlusion	Previous reports only
Martinez-Murcia et al. (2019)	${\text{AD Classification}}^{s^{\text{8 }}}$	ADNI	sMRI	FM visualization	Previous reports only
Uzunova et al. (2019)	${\text{Pathology Images, Types,}}^{s^{10}}$ and Lesion Classification	$d^{23}\;d^{24}\;d^{25}$	retinal OCT Brain MRI	VAE-perturbation Grad-CAM, Guided Backprop	Visual inspection quantitative validation
Gupta et al. (2019)	${\text{AD Classification}}^{s^{9}}$	ADNI	rs-fMRI	DeepLIFT	Quantitative evaluation, previous reports
Tang et al. (2019)	A*β* Pathologies Classification	UCD-ADC	${\text{WSI}}^{m^{3}}$	Guided Grad-CAM Occlusion Sensitivity	Previous reports only
Jin et al. (2020)	${\text{AD Classification}}^{s^{9}}$	In-house, ADNI	sMRI	Attention	Correlation Analysis
Essemlali et al. (2020)	${\text{AD Classification}}^{s^{8}}$	ADNI 2, ADNI-Go	DW-MRI	Gradients	Previous reports only
Qiu et al. (2020)	AD Classification	$d^{0}\;d^{20}\;d^{21}\;d^{22}$	sMRI	Model Transparency	Neuropathological and neurologist-level
Dyrba et al. (2020a)	${\text{AD Classification}}^{s^{9}}$	ADNI	T1-w	Deconvnet, Gradient ⊙ Input, DTD, LRP (*Z*/*ϵ*/*α* = 1, *β* = 0) Grad-CAM, Guided BP	Previous reports only
Leming et al. (2020)	ASD, Gender, Task v. Rest Classification	*d*^0^ *d*^5^ *d*^6^ *d*^12^ *d*^13^ *d*^14^ *d*^15^ *d*^16^ *d*^17^	fMRI	AM, CAM	Previous reports only
Magesh et al. (2020)	PD Classification	PPMI	${\text{SPECT}}^{m^{2}}$	LIME	Previous reports only
Abrol et al. (2020)	${\text{AD Classification}}^{s^{9}}$	ADNI	sMRI	Occlusion	Previous reports only
Biffi et al. (2020)	HCM, AD Classification	multi-site, ADNI	sMRI	FM visualization	Previous reports only
Lian et al. (2022)	${\text{AD Classification}}^{s^{9}}$	ADNI-1, 2/AIBL	T1w sMRI	Attention via CAM	Previous reports only
Bass et al. (2020)	Lesion Detection AD, Age Classification	*d*^0^ *d*^1^ *d*^14^	T1, T2 sMRI	Joint training, Occlusion, Grad-CAM, IG, GBP	Norm. Cross-correlation previous reports
Parmar et al. (2020)	${\text{AD Classification}}^{s^{9}}$	ADNI	rs-fMRI	FM visualization	Visual inspection
Hu et al. (2021d)	${\text{WRAT Classification}}^{s^{5}}$	PNC and $d^{8}$	nback-fMRI genomic data	Grad-CAM Guided Backprop	${\text{Previous reports, CPDB database}}^{\upsilon^{1}}$
Zhang et al. (2021b)	${\text{AD Classification}}^{s^{9}}$	ADNI-1,2,3	sMRI	Grad-CAM	Previous reports only
Kan et al. (2021)	Gender Classification	HCP	sMRI	Meaningful Perturbation Grad-CAM, GBP	Previous reports only
Rahman et al. (2022)	SZ, AD, and ASD Classification	*d*^3^ *d*^4^ *d*^5^	rsfMRI	Integrated Gradients (IG) Smoothgrad on IG	RAR framework, previous reports
Zhao et al. (2022)	SZ, ASD Classification	In-house, ABIDE	rsfMRI	LRP-*β*, Attention	Previous reports only
Chen et al. (2022d)	ASD Classification	ABIDE	sMRI	Grad-CAM	Previous reports only
Lin et al. (2022a)	SZ Classification	UNM IRB	${\text{cv rsfMRI}}^{m^{1}}$	Gradients, Grad-CAM	Previous reports only
Zhu et al. (2022)	${\text{AD Classification}}^{s^{9}}$	ADNI	sMRI	Joint Training	Predictive performance, previous reports
Oh et al. (2022b)	${\text{AD Classification}}^{s^{9}}$	ADNI	sMRI	(counterfactual) conditional GAN	Qualitative and quantitative analysis
Saboo et al. (2022)	Cognition Prediction	MCSA	sMRI, Diffusion MRI	LIME	Prior reports, exploratory analysis
Mahmood et al. (2022)	SZ, ASP, Dementia, Age, Gender prediction	FBIRN OASIS ABIDE HCP	rs-fMRI	Modular transparency attention mechanism	Compare with FNC, predictive performance
Wang et al. (2023a)	AD Classification	ADNI 1, 2, 3	T1 MRI	IG, LRP-*β*, Guided Grad-CAM	Meta-analysis on 77 VBM studies
Oliveira et al. (2024)	Lesion Classification	HCP	T1-w MRI	IG, Gradient SHAP, LRP DeepLIFT, Gradient, Deconvolution, GBP	Ground-truth matching of explanations
Bhattarai et al. (2024)	Cognition (MMSE) Prediction in AD/MCI	OASIS	${\text{MRI, Amyloid PiB-PET}}^{m^{5}}$	Deep SHAP	Ground-truth matching prior reports
Adey et al. (2024)	Sleep Stage Classification	Sleep European Data Format	Single-channel EEG	Multihead Attention	${\text{AOPC}}^{\upsilon^{8}}$
Leonardsen et al. (2024)	Dementia Classification, MCI to AD progression	8 Different datasets	T1-w sMRI	LRP	Visualization, meta-analysis, prior reports, quantitative validation, cognitive correlation
Wang et al. (2025)	AD, VD, and LBD Classification	NACC, ADNI	T1-w MRI	IG, SVM coefficient maps	${\text{Association between}}^{\upsilon^{7}}$ indices and cognitive scores, pathological, and imaging measures, prior reports
Brain Age Prediction					
Levakov et al. (2020)	Brain Age Prediction	15 Open databases	T1-w sMRI	Smoothgrad	Replicability/Similarity /Specificity tests
Lombardi et al. (2021)	Brain Age Prediction	ABIDE I	T1-w sMRI	SHAP, LIME	Intra-consistency, inter-similarity, correlation analysis
Ball et al. (2021)	Brain Age Prediction	PING	T1-w sMRI	SHAP	Previous reports only
Hofmann et al. (2022)	Brain Age Estimation	LIFE Adult Study	${\text{sMRI}}^{m^{0}}$	LRP (ϵ and *α* = 1, *β* = 0)	Via simulation/atlases /significance tests
Sihag et al. (2023)	Brain Age Prediction	OASIS-3 ADNI-1	sMRI	Joint Training	Robustness analyses of regional residuals
De Bonis et al. (2024)	Brain Age Prediction: Comparative Study	OpenBHB multisite dataset	T1-w MRI	SHAP, Deep SHAP Grad-CAM	Correlation analysis, XAI scores agreement analysis, prior reports
Hofmann et al. (2025)	Brain Age Estimation	LIFE Adult Study	${\text{sMRI}}^{m^{0}}$	LRP	Correlation with known biomarkers
Image Reconsturction/Synthesis/Image-to-Image Translation					
Bowles et al. (2018)	AD progression	ADNI	sMRI	Modular transparency, GAN	Longitudinal study
Liu et al. (2021)	AD and Alchoholic Dependence Classification	Synthetic dataset ADNI	T1, T2 sMRI	Counterfactual	Normalized cross-correlation
Ravi et al. (2022)	CN/MCI/AD 4D MRI Reconstruction	ADNI	T1 sMRI	Modular transparency	Qualitative and quantitative assessment
Jung et al. (2023)	Modeling AD progression	ADNI	T1-w sMRI	Generative, Counterfactual, Modular Transparency	Image quality scores, GAN metrics
Pombo et al. (2023)	Age Synthesis Sex Synthesis	UK Biobank OASIS-3	T1-w sMRI	GAN, Counterfactual, modular transparency	VBM, predictability, similarity metrics, ${\text{FID}}^{\upsilon^{2}}$
Liu et al. (2024)	AD vs. CN Classification Image Generation	ADNI-1, 2	MRI	Counterfactual images, and difference maps	SSIM, MSE, FID
Yeganeh et al. (2025)	Synthetic Image Generation AD vs. CN Classification	ADNI-1 OASIS-3	MRI, PET, Clinical Data	Counterfactual	${\text{FID}}^{\upsilon^{2}},$ ${\text{KID}}^{\upsilon^{3}}$, AUC
Brain Tumor Prediction					
Gaur et al. (2022)	Brain Tumor Classification	${\text{MRI}}^{d^{27}}$	MRI	SHAP, LIME	Not provided
Hossain et al. (2023)	Brain Tumor Detection and Classification	${\text{Kaggle}}^{d^{28}}$	sMRI	LIME	Visual inspection
Chel & Poh (2025)	Brain Tumor Classification	${\text{Kaggle Data}}^{d^{29}}$	MRI	LIME, Grad-CAM	Visual inspection
Brain Segmentation					
Zhang et al. (2020a)	Brain Tumor Segmentation	BraTS 2017, 2018 2019	Flair, T1 T1ce, and T2.	Attention (gate)	Hausdorff, Dice score
Natekar et al. (2020)	Brain Tumor Segmentation	BraTS 2018	Multiple MRI modalities	Grad-CAM, AM	Dice score
Esmaeili et al. (2021)	Brain Tumor Localization	${\text{TCGA}}^{d^{30}}$	T2-weighted, FLAIR MRI	Grad-CAM	Localization metrics correlation analysis
Ranjbarzadeh et al. (2021)	Brain Tumor Segmentation	BraTS 2018	T1, FLAIR T1C, and T2	Distance-wise attention	Hausdorff, Dice score, Sensitivity
Zeineldin et al. (2022)	Brain Glioma Grading Brain Tumor Segmentation	BraTS 2019 BraTS 2021	T1W, T1Gd, T2W, FLAIR	${\text{Seven SOTA methods}}^{i^{2}}$	Layer visualization previous reports
Graph-based Models					
Li et al. (2021)	ASD Classification Cognitive State Decoding	${\text{Biopoint}}^{d^{31}}$ HCP	Task fMRI	Joint training	Previous reports only
Abrate & Bonchi (2021)	ASD, ADHD Classification	${\text{ABIDE,}}^{d^{32}}$	fMRI	Counterfactual	Prior reports and GT
Wang et al. (2021a)	${\text{Individual Identification,}}^{s^{4}}$	HCP, ABIDE	rs-fMRI	Occlusion	Previous reports only
Hu et al. (2021b)	ASD Classification	ABIDE I	rs-fMRI	GNNExplainer, GRAD, and DeepLIFT	Predictability metrics and statistical tests
Azevedo et al. (2022)	Sex Prediction	UK Biobank HCP	T1, T2-FLAIR rs-fMRI, DWI	Joint training (association matrix)	A light comparison with literature
Zhang et al. (2023b)	Brain State Classification	HCP Q1, S1200	Task fMRI	Joint training, attention	No separate validation
Xu et al. (2024)	PD, AD, ASD Classification	Taowu, PPMI and Neurocon for PD, ADNI for AD, ABIDE for ASD	rsfMRI	Attention mechanism, Contrastive Graph Pooling	Visualization; prior reports
Han et al. (2025)	Motor Learning State Prediction	Collected from 20 healthy subjects	EEG	Node attention, node and edge masking, spectral and temporal ablation	Visual inspection prior literature

The papers are categorized by study task types, and key information is provided.

**I: Interpretability Methods.**

*i*^0^ SA-3DUCM: sensitivity analysis by 3D ultrametric contour map.

*i*^1^ 3D-ResNet-GAP: 3D-ResNet with global average pooling layer.

*i*^2^ Simonyan et al. (2013); Springenberg et al. (2014); Selvaraju et al. (2017); Sundararajan et al. (2017); Smilkov et al. (2017); Kapishnikov et al. (2021).

**V: Validation Methods.**

*υ*^0^ meta-analysis with NeuroSynth (Yarkoni et al., 2011).

*υ*^1^ CPDB: ConsensusPathDB-human.

*υ*^2^ FID: Fréchet Inception Distance (Heusel et al., 2017).

*υ*^3^ KID: Kernel Inception Distance.

*υ*^4^ SSIM: Structural Similarity.

*υ*^5^ MSE: Mean Square Error.

*υ*^6^ AUC: Area Under Curve.

*υ*^7^ DeepSPARE: Deep Signature of Pathology Atrophy Recognition.

*υ*^8^ AOPC: Area over the perturbation curve.

**S: Studies.**

*s*^0^ AD: Alzheimer’s Disease versus NC: Normal Controls.

*s*^1^ seeing images of body parts, faces, places, or tools.

*s*^2^ Multiple Sclerosis versus NC.

*s*^3^ SZ: Schizophrenia versus NC.

*s*^4^ ASD: Autism Spectrum Disorder versus NC/TD (typically developing).

*s*^5^ WRAT: wide range achievement test (LOW/HIGH).

*s*^6^ PD: Parkinson’s Disease versus NC.

*s*^7^ HCM: Hypertrophic cardiomyopathy versus NC.

*s*^8^ AD and other (clinical) variables.

*s*^9^ AD Variants (early/late/stable/progressive/amnestic) versus NC.

*s*^10^ IRF: intraretinal fluid/SRF: subretinal fluid/PED: pigment epithelium detachments.

*s*^11^ BT: Brain Tumor.

*s*^12^ VD: Vascular Dementia.

*s*^13^ LBD: Lewy body dementia.

**DM: Data Modality.**

*m*^0^ T1-w/FLAIR/SWI/DWI sMRI.

*m*^1^ complex-valued resting-state fMRI.

*m*^2^ SPECT DaTSCAN.

*m*^3^ WSI: whole slide imaging.

*m*^4^ Genomic Data.

*m*^5^ PiB PET: Pittsburgh Compound B Positron Emission Tomography.

**D: Datasets.**

*d*^0^ ADNI: Alzheimer’s Disease Neuroimaging Initiative.

*d*^1^ HCP: Human Connectome Project (HCP S1200 release).

*d*^2^ VIMS study.

*d*^3^ FBIRN: Function Biomedical Informatics Research Network.

*d*^4^ Open Access Series of Imaging Studies.

*d*^5^ ABIDE: Autism Brain Imaging Data Exchange.

*d*^6^ ABIDE: Autism Brain Imaging Data Exchange II.

*d*^7^ PNC: Philadelphia Neurodevelopmental Cohort.

*d*^8^ Illumina HumanHap 610 array, 500 array, Illumina Human Omni Express array.

*d^9^* LIFE Adult Study (Loeffler et al., 2015).

*d*^10^ UNM IRB: University of New Mexico Institutional Review Board.

*d*^11^ PPMI: Parkinson’s Progression Markers Initiative Database.

*d^12^* 1000 Functional Connectomes Project.

*d*^13^ Adolescent Brain Cognitive Development (ABCD) Study.

*d*^14^ UKB: UK Biobank.

*d*^15^ International Consortium for Brain Mapping database (ICBM).

*d*^16^ National Database for Autism Research.

*d*^17^ OpenfMRI.

*d*^18^ UCD-ADC: University of California, Davis Alzheimer’s Disease Center Brain Bank.

*d*^19^ PING: Pediatric Imaging, Neurocognition and Genetics.

*d*^20^ AIBL: Australian Imaging, Biomarker and Lifestyle Flagship Study of Ageing.

*d*^21^ FHS: Framingham Heart Study.

*d*^22^ NACC: National Alzheimer’s Coordinating Center.

*d*^23^ DUKE dataset (Farsiu et al., 2014).

*d*^24^ RETOUCH dataset https://retouch.grand-challenge.org/.

*d*^25^ LPBA40 dataset (Maier et al., 2017; Shattuck et al., 2008).

*d*^26^ MCSA: Mayo Clinic Study of Aging participants.

*d*^27^ Bhuvaji et al. (2020).

*d*^28^ Bhuvaji et al. (2020).

*d*^29^ Nickparvar (2021).

*d*^30^ TCGA: The Cancer Genome Atlas.

*d*^31^ Venkataraman et al. (2016).

*d*^32^ USCD: USC Multimodal Connectivity Database (Brown et al., 2012).

### 4.1 Backpropagation methods

#### 4.1.1 Gradient backpropagation

##### 4.1.1.1 CAM/Grad-CAM/Guided Grad-CAM

Yang et al. (2018) proposed three approaches to generate explanations. One of them, SA-3DUCM (sensitivity analysis by 3D ultrametric contour map), deals with sensitivity analysis of 3D-CNN by utilizing a hierarchical image segmentation approach, and the other two methods (3D-CAM (Zhou et al., 2016), 3D-GRAD-CAM (Selvaraju et al., 2017)) generate explanations by visually depicting activations on a spatial map. Occlusion sensitivity (OS) (Zeiler & Fergus, 2014) was used as a baseline based on the semantically meaningful brain segments obtained through 3DUCM. However, since OS does not consider correlations among segments, 3D Class Activation Mapping (3D-CAM) and 3D-Grad-CAM were used to address this limitation. Additional analysis revealed that *occlusion* methods fail to identify discriminative regions, whereas SA-3DUCM and 3D-CAM successfully identify some regions that align with human expert evaluation.

Hu et al. (2021d) proposed an interpretable DL framework to classify the cognitive ability of subjects from n-back fMRI data. This study combined brain functional connectivity (FC) data and single nucleotide polymorphism (SNP) data through multimodal fusion. They also leveraged Grad-CAM-guided (Selvaraju et al., 2017) convolutional collaborative learning. The goal was to extract useful brain mechanisms within and between brain FC and genetics. The proposed classifier outperformed the traditional ML classifiers. While all classifiers used some hand-engineered features, the low performance of traditional ML classifiers could be attributed to the further dimensionality reduction of hand-engineered features. Notably, the model identified numerous significant FCs for the low WRAT (Wide Range Assessment Test) group, while a smaller number of significant FCs were identified for the high WRAT group. To validate the identified SNPs, the authors used the ConsensusPathDB-human (CPDB) database, which provided intuitive explanations for the discriminative behavior of the model.

**Fig. 3.**
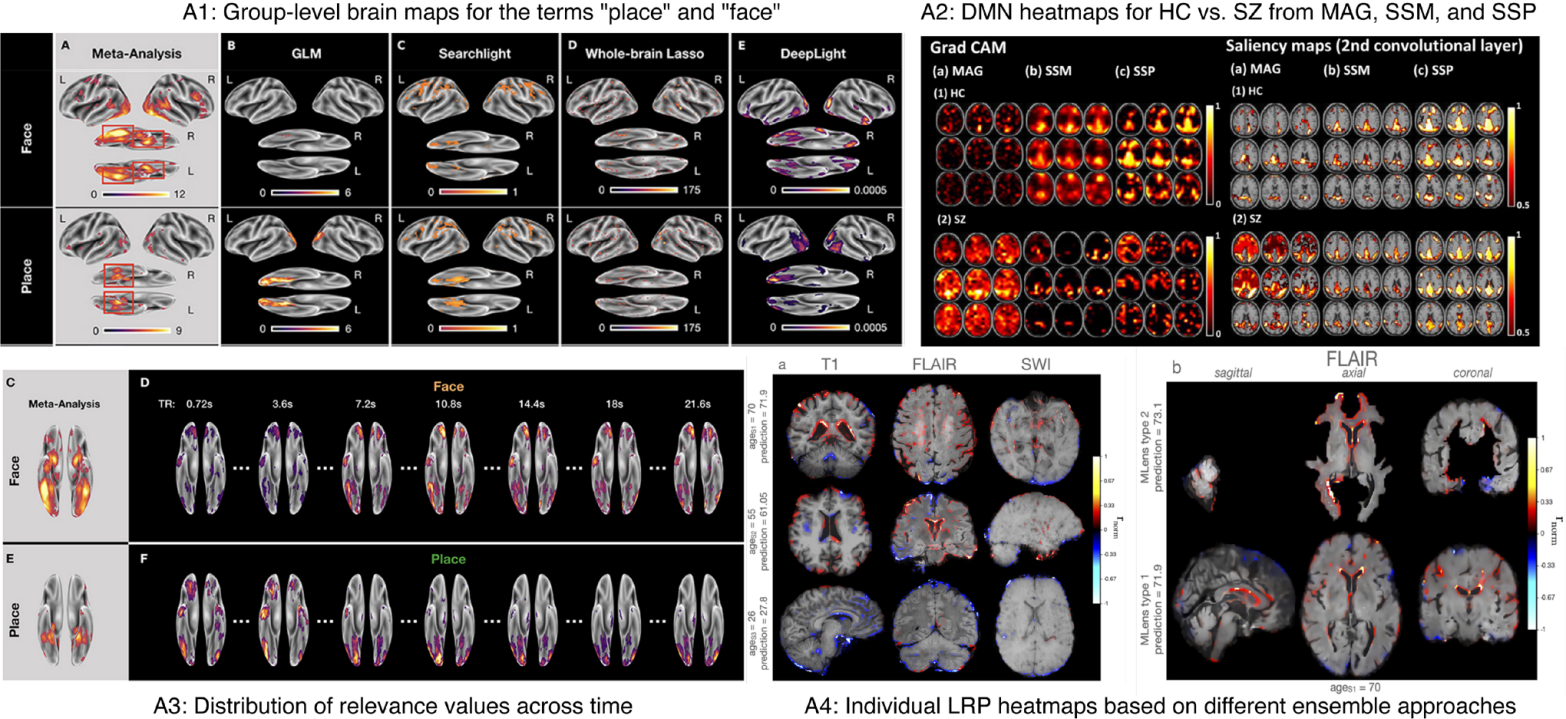
(A1) Group-level brain maps for DeepLight and other baselines. Column (A) ROIs obtained from a meta-analysis. (B–D) Group-level brain maps using other baselines. (E) Group-level brain maps from DeepLight. (A2) DeepLight generated distribution of group-level relevance values for the two stimulus classes—“face” and “place.” (C and E) Results of the meta-analysis to establish the target maps. (D and F) Group-level relevance distribution across time. The similarity of the brain maps with the meta-analysis maps was found to be very high (Image is adapted from Thomas et al. (2019)). (A3) Default Mode Network (DMN) saliency maps at the two convolutional layers and Grad-CAM heatmaps for (a) magnitude (MAG) maps, (b) spatial source magnitude (SSM) maps, and (c) spatial source phase (SSP) maps extracted from (1) a healthy control (HC) and (2) a (Schizophrenia) SZ individual. The activations inside the DMN were more pronounced within the second layer. SSP localized DMN regions with opposite strengths for HC and SZ. In contrast, SSM and MAG provided maps were inconsistent and had undesirable effects (Image is adapted from Lin et al. (2022a)). (A4) Individual LRP heatmaps trained on (a) multi-level ensemble of whole brain T1, FLAIR, and SWI data. The maps highlight important brain regions contributing to the subject’s age. (b) LRP heatmaps produced using region-based ensembles (top row) and whole-brain FLAIR data (bottom row). The identified areas were around the ventricles and subject-specific sulci (Image is adapted from Hofmann et al. (2022)).

Lin et al. (2022a) proposed a 3D-CNN model that utilizes spatial source phase (SSP) maps derived from complex-valued fMRI data to differentiate between schizophrenia patients and normal controls (NC). Their study found that SSP maps were more effective in prediction than magnitude maps (MAG) extracted from magnitude-only fMRI data, as well as spatial source magnitude maps (SSM) derived from complex-valued fMRI data. The authors generated saliency maps and applied Grad-CAM (Selvaraju et al., 2017) to identify and understand the regions of interest that contributed to the model’s predictions. A snapshot of the generated explanations at the subject level is shown in Figure 3 *Panel A1*.

Zhang et al. (2021b) utilized a combination of residual network and self-attention techniques to carry out two classification tasks involving sMR images: distinguishing AD from NC and pMCI from sMCI. The study demonstrated that the utilization of residual networks was more effective in learning from sMR images compared with alternative convolutional network variants, such as 3D-VGGNet. Furthermore, the incorporation of self-attention contributed to an enhancement in classification performance. To further explain individual predictions, the authors employed 3D Grad-CAM (Selvaraju et al., 2017).

Leming et al. (2020) employed a diverse collection of fMRI datasets and applied a deep CNN for three classification tasks: ASD, gender, and resting/tasks using FC. The study found that the DL model was effective in achieving accurate classification results, especially when the datasets consisted of a mixture of multi-site collections. To compute FC, the researchers utilized the 116-area automated anatomical labeling (AAL) parcellation template (Tzourio-Mazoyer et al., 2002) and operated at four different wavelet frequency scales. They utilized the CAM technique (Zhou et al., 2016) to identify the brain’s prominent spatial elements (connectome) associated with the predictions. Additionally, activation maximization provided insights into the critical predictive features suitable for classification. However, due to the considerable variation in the ensemble’s accuracies, it should be noted that the identified areas may not fully characterize ASD.

Esmaeili et al. (2021) employed three popular DL models to distinguish healthy and tumor MR images. The authors utilized Grad-CAM (Selvaraju et al., 2017) to localize tumor lesions. The localization ability was measured using multiple metrics, revealing the DL models’ effectiveness in tumor localization.

##### 4.1.1.2 Gradients and GBP

Rieke et al. (2018) introduced a 3D-CNN to classify AD patients from NC. To provide explanations for the classification decisions, the authors employed several visualization techniques, including GRAD (Simonyan et al., 2013), GBP (Springenberg et al., 2014), occlusion (Zeiler & Fergus, 2014), and brain area occlusion. They observed that gradient-based visualization methods exhibited a more distributed pattern across the brain, unlike occlusion and brain area occlusion techniques, which yielded relevance scores concentrated in specific regions. In fact, distributed relevance is not feasible for occlusion-based methods because of the limited size of the patch. While all four methods highlighted certain regions relevant to AD, such as the inferior and middle temporal gyrus, the distribution of relevance scores varied significantly among patients. Notably, the relevance maps of certain patients predominantly focused on the temporal lobe, while for others, larger cortical areas were the center of attention.

Essemlali et al. (2020) trained a BrainNet CNN on diffusion-weighted MRI connectivity patterns to distinguish between AD, MCI, and NC. Using gradient heatmaps (Simonyan et al., 2013), the study generated post hoc explanations and identified group-level structural differences in connected regions that were consistent with the literature reports.

Oh et al. (2019) proposed a learning model based on CNN for performing four classification tasks that involve classifying various stages of AD from NC and distinguishing pMCI from sMCI. The model was pretrained in an unsupervised manner using a convolutional autoencoder. The study also utilized a gradient-based method (Simonyan et al., 2013) to visualize features that are sensitive to predictions. Through post hoc interpretability, it was revealed that the temporal and parietal lobes play a crucial role in discriminating between AD patients and controls.

##### 4.1.1.3 IG and smoothgrad

Rahman et al. (2022) introduced an interpretable DL framework that uses a self-supervised pretraining technique to learn from healthy subjects. They then transferred this knowledge to improve predictive performance and interpretability in multiple downstream studies with limited sample sizes. The framework utilized IG (Sundararajan et al., 2017) and its smoothgrad (Smilkov et al., 2017) variant to gain insights into the predictions. In addition to qualitative assessment, the framework proposed a quantitative evaluation method to demonstrate the strong predictability of the identified important regions. Table 4 summarizes our review of selected studies that applied transfer learning to various neuroimaging modalities.

**Table 4.** Summary of selective papers using transfer learning in neuroimaging.

Authors & year	Task	Modality	Transferred from	XAI method
He et al. (2018)	Cognitive Deficit Prediction	rs-fMRI	SSAE^b^ on autism	Gradients
Vakli et al. (2018)	Age Classification/Regression	rs-fMRI	Public and large dataset	Occlusion
Demyanchuk et al. (2019)	Hydrocephalus Classification	sMRI	ImageNet (m3)	Grad-CAM
Oh et al. (2019)	AD Variants Classification	sMRI	CAE^a^ to AD v NC to pMCI v sMCI	Grad, Occlusion
Chen et al. (2020)	Cognitive Deficit Prediction	DTI^c^	ImageNet dataset	Grad-CAM
Nguyen et al. (2020)	7/Motor/WM TS^f^ decoding	task fMRI	Intra-task, inter-task	Grad-CAM
Etminani et al. (2021)	AD Variants Classification	18F-FDG-PET	ImageNet (m1 m2)	Occlusion, G-CAM
Bae et al. (2021)	MCI to DAT^d^ Progression	sMRI	Pretrain on DAT vs. NC	Occlusion
Ni et al. (2021)	AD Detection	ECD SPECT FDG - PET	ImageNet (m1) to FDG-PET to ECD SPECT	Grad-CAM
Zhang et al. (2021a)	TSS^e^ Classification	MRI Diffusion	Stroke detection	Grad-CAM
Danso et al. (2021)	Dementia Risk Prediction	Survey data	Older to younger cohort	SHAP
Ban et al. (2022)	AD Diagnosis	sMRI	ImageNet (m4)	Grad-CAM
Oktavian et al. (2022)	AD Classification	sMRI	ImageNet (m4)	Grad-CAM
Lu (2022)	ASD Classification	rs-fMRI	Controls to target task domain adaptation	LRP
Gao et al. (2022)	Sex, ASD Classification	sMRI	ImageNet (m2 m5) to Sex to ASD	Occlusion
Rahman et al. (2022)	SZ, AD, ASD Classification	rs-fMRI	Healthy controls^g^	IG, Smoothgrad
Kim et al. (2023)	Sex, Age, and Cognitive Intelligence Prediction	rs-fMRI	Two constrastive losses^g^	IG-Smoothgrad sQuare
Oliveira et al. (2024)	Lesion Classification	T1-w MRI	Natural images and MRI slices	IG, GradSHAP, LRP DeepLIFT, Gradient, Deconvolution, GBP

a CAE: convolutional autoencoder.

b SSAE: stacked sparse autoencoder.

c DTI: diffusion tensor imaging.

d DAT: dementia of Alzheimer’s type.

e time since stroke onset.

f task state.

g self-supervised contrastive pretraining.

m1 trained on Inception V3.

m2 trained on ResNet – 50.

m3 trained on ResNet – 34.

m4 trained on ResNet – 18.

m5 trained on DenseNet-121.

Levakov et al. (2020) proposed an ensemble of CNNs to study brain age. The idea of ensemble was used to analyze the model uncertainty behavior and *smoothgrad* (Smilkov et al., 2017) was used to generate population-wise explanations of anatomical brain regions. Population-based map for each ensemble was produced by averaging all the volumes in the test set. To generate the global population-based map, they aggregate population-based maps from each CNN by taking the median value for each voxel across the ensembles. While this approach highlights important areas in the brain, this approach was not able to determine the direction of the contributions of the regions toward brain age.

Wang et al. (2023a) utilized IG (Sundararajan et al., 2017), LRP (Bach et al., 2015), and Guided Grad-CAM (Selvaraju et al., 2017) techniques to visualize CNN models used in AD classification. All the three approaches were more aligned with the meta-analysis map than SVM activation patterns. However, the meta-analysis demonstrated that IG heatmaps exhibited a stronger emphasis on the hippocampus in comparison with Guided Grad-CAM and LRP heatmaps, which is consistent with established biomarkers for AD. In a later investigation, Wang et al. (2025) developed a novel multi-label 3D DL framework (ResNet) trained on neuropathology-confirmed cases to distinguish AD, vascular dementia (VD), and Lewy body dementia (LBD) from T1 MRI. The authors also introduced DeepSPARE single-value indices derived from model probabilities. The produced IG heatmaps reveal pathology-specific spatial signatures (e.g., bilateral hippocampal regions for AD, white-matter patterns for VD, occipital alterations for LBD) and show that the DeepSPARE indices correlate strongly with cognitive scores, neuropathology measures, and neuroimaging measures. A separate validation on an independent ADNI cohort was also conducted to replicate findings.

**Fig. 4.**
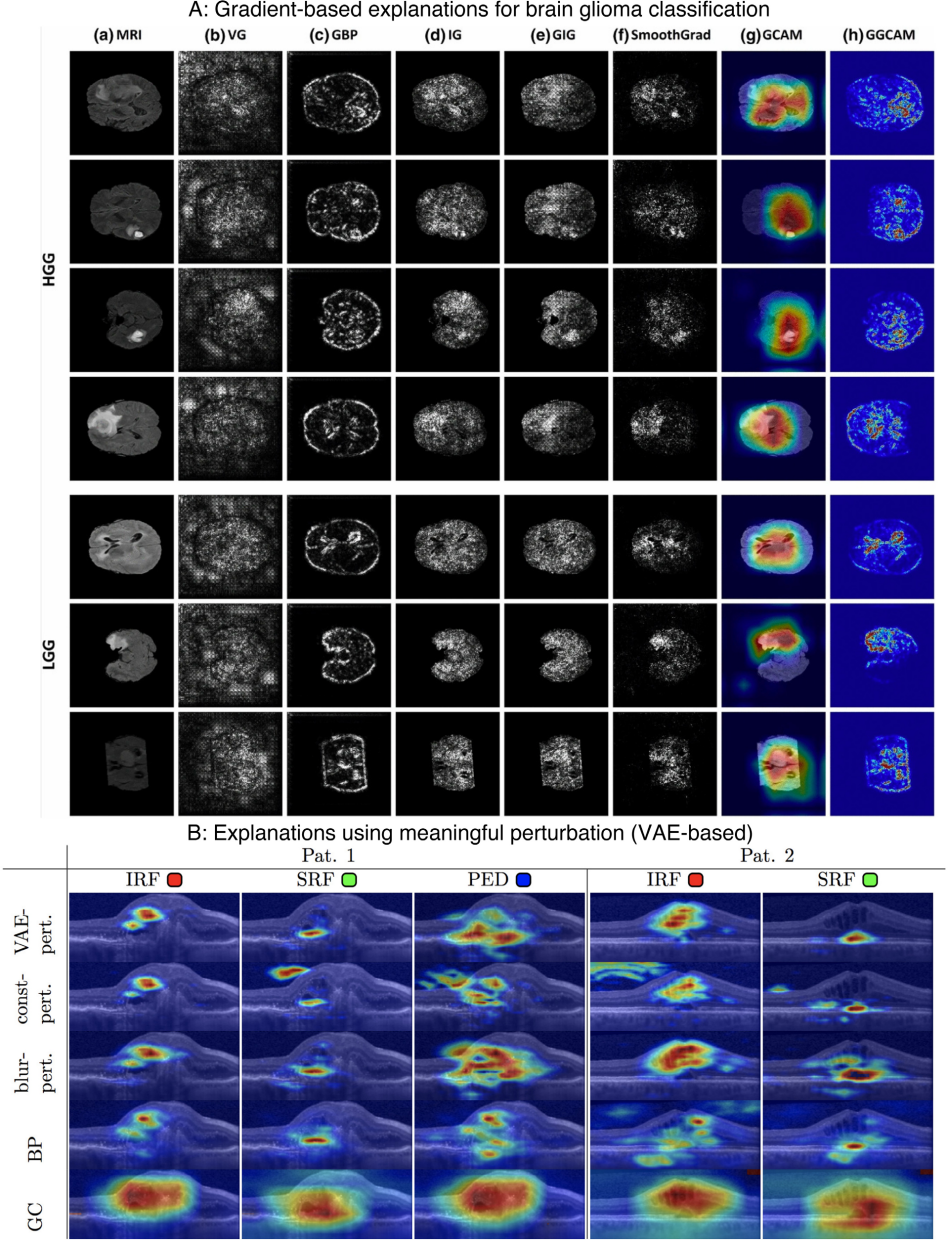
(A) Explanations generated using gradient-based methods for high-grade gliomas (HGG) and low-grade gliomas (LGG) cases. Maps (b–f) highlight salient features, while maps in (g, h) highlight salient regions in the input space that drove the predictions (Image is adapted from Zeineldin et al. (2022)). (B) Explanations for two patients by different approaches for a classifier that distinguishes disease pathologies (intraretinal fluid (IRF), subretinal fluid (SRF), and pigment epithelium detachments (PED)). VAE-based perturbation closely aligns with the disease pathologies. Blur perturbation performs well but is only suitable for small structures. Constant perturbation does the worst for explanations. Grad-CAM identifies the infected regions but suffers from poor resolution, whereas GBP is noisy and not class discriminative (Image is adapted from Uzunova et al. (2019)).

Zeineldin et al. (2022) compared seven gradient-based explanation methods for MR image classification and segmentation tasks: GRAD (Simonyan et al., 2013), *smoothgrad* (Smilkov et al., 2017), IG (Sundararajan et al., 2017), GBP (Springenberg et al., 2014), Grad-CAM, Guided Grad-CAM (Selvaraju et al., 2017), and Guided Integrated Gradients (Kapishnikov et al., 2021). Guided Grad-CAM showed better localization for brain glioma classification, while *smoothgrad* identified the best discriminative regions. For segmentation, *smoothgrad* was the most robust to noise, while Grad-CAM visualized the most discriminative regions. Refer to Figure 4 *Panel A* for sample heatmaps.

#### 4.1.2 Modified/relevance backpropagation

##### 4.1.2.1 LRP

DeepLight (Thomas et al., 2019) utilizes recurrent and convolutional elements to analyze whole-brain activity linked to four cognitive states. It takes brain volumes as input and predicts corresponding cognitive states, generating a sequence of predictions for each time point. The LRP (Bach et al., 2015) method generates explanations for each volume prediction, attributing relevance to voxel levels. Figure 3 *Panel A3* compares group-level maps generated by DeepLight and other baselines. A meta-analysis of the four cognitive states was conducted using NeuroSynth, a cognitive state–brain association database, identifying several ROIs associated with each state. The *body* state is linked to the upper parts of the middle and inferior temporal gyrus, postcentral gyrus, and right fusiform gyrus. The *face* state is associated with the fusiform gyrus and amygdala. The *place* state is connected to the parahippocampal gyrus. The *tool* state is associated with the upper left middle and inferior temporal gyrus, and left postcentral gyrus. While all baselines can identify brain activity associated with the stimulus classes, DeepLight, combined with %-LRP, demonstrates higher accuracy and consistency in predicting cognitive states. Figure 3 *Panel A4* shows the spatio-temporal distribution of brain activity during the first experiment block for the *place* and *tool* states. Initially, DeepLight exhibits uncertainty about the cognitive state but quickly improves its confidence, gradually aligning with the target maps from the NeuroSynth meta-analysis. As brain encoding models can reveal functional descriptions of the brain and decoding models have the potential to uncover the connection between brain activity and behavior, we suggest readers refer to some encouraging DL papers (Du et al., 2019; Glaser et al., 2020; Kriegeskorte & Douglas, 2019; Naselaris et al., 2011; Wen et al., 2018) in this direction.

Eitel et al. (2019b) leveraged LRP (Bach et al., 2015) to uncover the rationale behind decisions made by 3D CNNs trained to diagnose multiple sclerosis (MS). The identified features revealed that CNN is able to identify relevant imaging biomarkers, such as individual lesions, lesion location, non-lesional white matter, or gray matter areas, which are considered as established MS biomarkers.

Böhle et al. (2019) utilized LRP (Bach et al., 2015) and GBP (Springenberg et al., 2014) (as a baseline) to elucidate the decisions of a CNN model. LRP was favored over gradient-based approaches as it breaks down the output in terms of contributions in the input space, whereas gradient-based approaches identify the most sensitive voxels associated with this outcome. Although both LRP and GBP successfully localized important regions, GBP exhibited comparatively lower contrast in importance scores between group-wise heatmaps when compared with LRP. For objective evaluation of the heatmaps, a scalable brain atlas (Bakker et al., 2015) was employed.

Zhao et al. (2022) proposed a hybrid DL architecture to combine sequential temporal dynamics (TCs) and functional network connectivity (FNCs). An attention module on top of C-RNN was used to extract temporal dynamic dependencies from TCs. LRP-β (Montavon et al., 2019) was used to identify the most group-discriminative FNC patterns and the attention module was used to identify discriminative independent components (ICs) during test time. Yan et al. (2017) proposed a fully connected network for FNC data to classify SZ patients from controls and achieved improved performance compared with other ML and CNN models. However, the authors only used LRP as an explanation layer during inference time and did not thoroughly investigate or validate the class-discriminative features generated by LRP.

Hofmann et al. (2022) proposed robust models using ensembles of CNNs and leveraged LRP (Bach et al., 2015) to identify significant features associated with brain age. These models effectively captured aging at different scale changes and identified related risk factors for divergent brain age. The relevance scores of three major brain components (gray matter, white matter, and cortical spinal fluids) showed a linear correlation with age (Fig. 3 *Panel A4*). While the prior study confirmed only preliminary aspects of the decision-making process using the LRP method, those highlighted areas in the heat maps were not critically analyzed for neurobiological relevance. In a follow-up study, Hofmann et al. (2025) claimed that the proposed brain age (BA) estimation model consisting of multi-level ensembles highly relied on known neuroimaging biomarkers associated with age-related brain changes. To demonstrate whether the model is relying on the well-known imaging biomarkers, the authors extracted LRP-based relevance maps (heat maps) for the brain age predictions and found strong correlations between the heat maps and known imaging markers, such as ventricular volume, temporal–parietal cortical thickness and volume, cerebellar gray matter volume, and frontal–occipital white matter tracts. However, some known age-related biomarkers (e.g., perivascular spaces) either were not used by the model or incorrectly attributed (e.g. cerebellum) for the BA estimation. Compared with most earlier studies where the focus of explainability was primarily on identifying relevant patterns in the data, Leonardsen et al. (2024) investigated the clinical utility of LRP-generated explanations for Alzheimer’s disease (AD) patients, aimed at individualized disease characterization. Beyond identifying relevance patterns, the study constructed individual morphological profiles from LRP heat maps and provided extensive validation showing how interpretable components of these explanations contribute to AD progression at both individual and population levels. This work represents one of the earliest demonstrations of the translational value of post hoc XAI approaches in neuroimaging. However, the framework still requires validation in clinical settings with larger and more diverse cohorts and could be extended to incorporate multimodal data. Importantly, the authors also cautioned that relevance maps often rely on contextual dependencies rather than strictly localized brain regions. So, clinical interpretation by experts remains crucial to ensure their reliable application in practice.

##### 4.1.2.2 DeepLIFT, Deconvnet, and Deep Taylor Decomposition

Gupta et al. (2019) proposed a feed-forward network for classifying NC versus AD, NC versus MCI, and MCI versus AD. They utilized FC features from anatomically and functionally diverse ROIs selected from resting-state fMRI. Explanations were generated using DeepLIFT (Shrikumar et al., 2017) and were further evaluated through recursive feature elimination and the retraining of the model using only the relevant subset of features. For each task, the retrained models achieved higher accuracy than the original performance, despite using fewer salient features.

Dyrba et al. (2020a) compared six post hoc methods for a 3D CNN model: Deconvnet (Zeiler & Fergus, 2014), gradient ⊙ input (Shrikumar et al., 2016), Deep Taylor Decomposition (Montavon et al., 2017), LRP (Bach et al., 2015), Grad-CAM (Selvaraju et al., 2017), and GBP (Springenberg et al., 2014). The study found that Deep Taylor Decomposition and LRP with α=1, β=0 , which are modified backpropagation methods, generated clinically useful explanations that were focused and aligned with the existing literature on the disorders. However, Grad-CAM and GBP explanations appeared more scattered and loosely connected to previous reports. Refer to Table 5 for the summary of the reviews on post hoc methods.

**Table 5.** Summary of the most widely used post hoc methods across various neuroimaging tasks.

XAI method	Uses in neuroimaging (total publications)	Major modalities	Major tasks	Highlights
Occlusion Sensitivity	Kwak et al. (2022a, 2022b); Polson et al. (2022a); Zheng et al. (2022); Lu et al. (2022a); Lee et al. (2022b); Bi et al. (2022); Hahn et al. (2022); Ning et al. (2022); Vyas et al. (2022); Wood et al. (2022a); Polson et al. (2022b); Bordin et al. (2022); Etminani et al. (2022); Gao et al. (2022); Lee et al. (2022a); Qu et al. (2021); See et al. (2021); Abrol et al. (2021); Zou et al. (2021); Murugan et al. (2021); Leming & Suckling (2021); Etminani et al. (2021); Dyrba et al. (2021); Wood et al. (2021); Turkan & Tek (2021); Bass et al. (2022); Kwak et al. (2021); Wang et al. (2021a); Bae et al. (2021); Thomas et al. (2020); Mostafa & Cheng (2020); Kwak et al. (2020); Oh et al. (2020); Leming & Suckling (2020); Bass et al. (2020); Abrol et al. (2020); West et al. (2019); Oh et al. (2019); Islam & Zhang (2019); Kam et al. (2019); Liu et al. (2019c); Eitel et al. (2019a); Esmaeilzadeh et al. (2018); Vakli et al. (2018); Liu et al. (2018); Chambon et al. (2018); Rieke et al. (2018); Yang et al. (2018); Gutiérrez-Becker & Wachinger (2018) (50)	PET/ tau-PET/ ${\text{MRI}}^{⋆}$/fMRI	$\text{AD}{⋆}^{c}\text{​}/{\text{BA}}^{e}\text{​}/$ ; $\begin{array}{l} {\text{Sex}}^{c}\text{​}/{\text{MCI}}^{c}\text{​}/\\ {\text{PD}}^{c}\text{​}/{\text{ASD}}^{c} \end{array}$	Studies mostly used CNN^*^; some studies employed graphical networks and transfer learning;
Gradients	Lin et al. (2022a); Aslan & Akin (2022); Zeineldin et al. (2022); Thomas et al. (2022); Cui & Weng (2022); Sokolova (2021); Fernández et al. (2021); Hu et al. (2021b); Wilms et al. (2021); Liu et al. (2021); Pianpanit et al. (2021); McClure et al. (2020); Lopatina et al. (2020); Sánchez Fernández et al. (2020); Oh et al. (2019); Islam & Zhang (2019); Rieke et al. (2018); Garg et al. (2017b) (18)	PET/EEG/ MRI^*^/fMRI/ SPECT	$\begin{array}{l} SZ^{c}/AD^{*c}/\\ PD^{c}/BT^{s}/\\ MSCS^{i} \end{array}$	Studies mostly used CNN^*^; some studies used graph attention and transfer learning;
Grad-CAM	Fujiwara & Ushiba (2022); Ushizima et al. (2022); Liu et al. (2022b); Zhang et al. (2022); Huang et al. (2022b); Prats-Climent et al. (2022); Inglese et al. (2022); Liu et al. (2022a); Aslan & Akin (2022); Zhang et al. (2023a); Li et al. (2022b); Ding et al. (2022); Tasci & Tasci (2022); Odusami et al. (2022); Ban et al. (2022); Yu et al. (2022); Ko et al. (2022); Lu et al. (2022a); Jimeno et al. (2022); Zihni et al. (2022); Delbarre et al. (2022); Dasanayaka et al. (2022); Shaban & Amara (2022); Altuve & Pérez (2022); Lu et al. (2022b); Gao et al. (2023); Yu et al. (2023); Huang et al. (2022a); Guo et al. (2022); Kumar & Michmizos (2022); Jonas et al. (2022); Ahmed et al. (2022); Wang et al. (2023a); Fu et al. (2022); Thomas et al. (2022); Zhou et al. (2022); Oh et al. (2022b); Chen et al. (2022d); Giudice et al. (2022); Kurmi et al. (2022); Williamson et al. (2022); Polson et al. (2022a); Termine et al. (2022); Maqsood et al. (2022); Li et al. (2022a); Zia et al. (2022); Qin et al. (2022); Zeineldin et al. (2022); Oktavian et al. (2022); Luckett et al. (2022); Watanabe et al. (2022); Polson et al. (2022b); Devika et al. (2022); Garcia & Kelly (2022); Lin et al. (2022a); Sokolova (2021); Brüningk et al. (2021); Wang et al. (2021d, 2021c); Ocasio & Duong (2021); Fernández et al. (2021); Ni et al. (2021); Azcona et al. (2021); Mishinov et al. (2021); Kuijs et al. (2021); Mathur et al. (2021); Jeong et al. (2021); Treacher et al. (2021); Raju et al. (2021); Patel et al. (2021); Leming et al. (2021); Messina et al. (2021); Zhang et al. (2021a); Mohebbian et al. (2021); Liu et al. (2021); Zihni et al. (2021); Song et al. (2021); Gao et al. (2021); Hepp et al. (2021); Ozsahin et al. (2021); Kim et al. (2021c); Li & Yang (2021); Yasaka et al. (2021); Hu et al. (2021d); Mei et al. (2021); Shaji et al. (2021); Pianpanit et al. (2021)	EEG/MRI^*^/ fMRI/SPECT/ tau-PET/ FDG-PET/ CT/MEG	$\begin{array}{l} BA^{e}/AD^{*c}/\\ PD^{c}/SZ^{c}/\\ ASD^{c}/BT^{s}/\\ Sex^{c}/MCI^{c}/\\ MSCS^{i}/MDD^{c} \end{array}$	Studies mostly used CNN^*^; some studies used attention mechanism and transfer learning; also used in graph convolution and recurrent nets; Grad-CAM often served as a baseline;
	Turkan & Tek (2021); Bass et al. (2022); Zhang et al. (2021b, 2021c); Fan et al. (2021); Pati et al. (2021); Tomaz Da Silva et al. (2021); Menon & Krishnamurthy (2021); Youn et al. (2021); Ullanat et al. (2021); Etminani et al. (2021); Ko et al. (2021); Wang et al. (2021b); Dandıl & Karaca (2021); Leming & Suckling (2021); Hu et al. (2021a); Wilms et al. (2021); Yee et al. (2021); Esmaeili et al. (2021); Liu et al. (2020); Qu et al. (2020); Kan et al. (2020); Leming & Suckling (2020); Li et al. (2020a); Pereira et al. (2020); Nguyen et al. (2020); Côté-Allard et al. (2020); Kim & Ye (2020); Ke et al. (2020); Akiyama et al. (2020); Nishi et al. (2020); Da Silva et al. (2020); Aslan & Akın (2020); Alegro et al. (2020); Vakli et al. (2020); Sánchez Fernández et al. (2020); Pelka et al. (2020); Bass et al. (2020); Dyrba et al. (2020a); Azcona et al. (2020); Yee et al. (2020a, 2020b); Natekar et al. (2020); Feng et al. (2020); Tang et al. (2020); Chen et al. (2020); Wu et al. (2020); Kan et al. (2021); Wang et al. (2019a); Iizuka et al. (2019a); Alegro et al. (2019); Iizuka et al. (2019b); Lala (2019); Gao et al. (2019); Chen et al. (2019b); Demyanchuk et al. (2019); Feng et al. (2019); Noguera et al. (2019); Wang et al. (2019b); Bermudez et al. (2019); Hilbert et al. (2019); Lee et al. (2019b); Petrov et al. (2018); Pereira et al. (2018a); Baumgartner et al. (2018); Golkov et al. (2018); Yang et al. (2018); Andreotti et al. (2018); Feng et al. (2018); Pominova et al. (2018); Garg et al. (2017a, 2017b) (total 160)	—	—	—
IG	Wang et al. (2023a); Yu et al. (2023); Rahman et al. (2022); Supekar et al. (2022b); Wadhera & Mahmud (2022); Lewis et al. (2023); Narazani et al. (2022); Thomas et al. (2022); Spitzer et al. (2022); Sarasua et al. (2022); Richie-Halford et al. (2022a); Kim et al. (2022c); Ferrante et al. (2022); Richie-Halford et al. (2022b); Lin et al. (2022b); Jiang & Miao (2022); Zeineldin et al. (2022); Prasad et al. (2022); Shen et al. (2022); Cui & Weng (2022); Supekar et al. (2022a); Choi et al. (2022); Oh et al. (2022b); Polson et al. (2022b); de Mello et al. (2021); de la Fuente et al. (2021); Manjunatha & Esfahani (2021); Bass et al. (2022); Xin et al. (2021); Lin et al. (2021a); Pan et al. (2021); Bass et al. (2020); Varatharajah et al. (2019); Liu et al. (2019a); Baumgartner et al. (2018) (35)	EEG/MRI^*^/ fMRI/PET	$\begin{array}{l} \text{AD}{⋆}^{c}\text{​}/{\text{SZ}}^{c}\text{​}/\\ {\text{ASD}}^{c}\text{​}/{\text{MCl}}^{c} \end{array}$	Studies mostly used CNN^*^; also used with recurrent network and attention; often served as a baseline for other post hoc or generative approaches;
Smoothgrad	Zeineldin et al. (2022); Thomas et al. (2022); Rahman et al. (2022); Wang et al. (2022); Wood et al. (2022b); Mayor-Torres et al. (2021); Sokolova (2021); McClure et al. (2020) (8)	MRI^*^/fMRI/ EEG	$\begin{array}{l} {\text{AD}}^{c}\text{​}/{\text{SZ}}^{c}\text{​}/\\ {\text{ASD}}^{c}\text{​}/{\text{BT}}^{s} \end{array}$	Use in neuroimaging is limited; few studies found it useful; often used for benchmarking post hoc methods;
GBP	Cui & Weng (2022); Wood et al. (2022b); Zeineldin et al. (2022); Polson et al. (2022a); Yu et al. (2023); Wood et al. (2022a); Thomas et al. (2022); Polson et al. (2022b); Tinauer et al. (2022); Cho & Wallraven (2022); Oh et al. (2022b); Yee et al. (2021); Pati et al. (2021); Abrol et al. (2021); Hu et al. (2021c); Bron et al. (2021); Liu et al. (2021); Sokolova (2021); Pianpanit et al. (2021); Cruciani et al. (2021b); Zhang et al. (2021d); Bass et al. (2022); Shi et al. (2020); Kan et al. (2020); Li et al. (2020b); Wang et al. (2020); Yee et al. (2020a); Folego et al. (2020); Yee et al. (2020b); Kan et al. (2021); Dyrba et al. (2020a); Bass et al. (2020); Islam & Zhang (2019); Eitel et al. (2019a); Böhle et al. (2019); Rieke et al. (2018); Golkov et al. (2018); Pereira et al. (2018a); Baumgartner et al. (2018) (39)	${\text{EEG/MRI}}^{⋆}$/ fMRI/PET/ FDG-PET/ SPECT	$\begin{array}{l} \text{AD}{⋆}^{c}\text{​}/{\text{BA}}^{e}\text{​}/\\ {\text{PD}}^{c}\text{​}/{\text{BT}}^{s}\text{​}/\\ {\text{MSCS}}^{i}\text{​}/ \end{array}$ AIS*^i^* /TSD	studies mostly used CNN; used for diverse set of tasks; often used with Grad-CAM; often used as baseline;
DeepLIFT	Prasad et al. (2022); Thomas et al. (2022); Cui & Weng (2022); Oh et al. (2022b); Gupta et al. (2022); Taylor et al. (2022); Gupta et al. (2021a); Pianpanit et al. (2021); Bennett et al. (2021); Hu et al. (2021b); Mane et al. (2021); Lopatina et al. (2020); Gupta et al. (2019) (13)	EEG/MRI/ fMRI/SPECT	$\begin{array}{l} AD{⋆}^{c}\text{​}/{\text{PD}}^{c}\text{​}/{\text{ASD}}^{c}\text{​}/\\ {\text{MSCS}}^{i}\text{​}/\text{FC }\\ \text{in AD}/\text{FC }\\ \text{in MCI} \end{array}$	Use in nuroimaging is limited; used in graph attention network; used in EEG-based BCI; often used as a baseline;
LRP	Tinauer et al. (2022); Hofmann et al. (2022); Pohl et al. (2022); Korda et al. (2022); Chen et al. (2022c); Torres et al. (2022); Sudar et al. (2022); Kim et al. (2022b); Cui & Weng (2022); Nazari et al. (2022); Ellis et al. (2022b); Lu (2022); Oh et al. (2022b); Ellis et al. (2022a); Klingenberg et al. (2023); Hofmann et al. (2022); Zhao et al. (2022); Taylor et al. (2022); Thomas et al. (2022); Wang et al. (2023a); Deatsch et al. (2022); Ellis et al. (2021b); Dyrba et al. (2021); Nazari et al. (2021); Bang et al. (2021a); Mayor-Torres et al. (2021); Ellis et al. (2021a); Pena et al. (2021); Raison et al. (2021); Bennett et al. (2021); Cruciani et al. (2021a, 2021b); Jeon et al. (2021); Eitel et al. (2021); Dong et al. (2021); Korda et al. (2021); Wang (2020); Lopatina et al. (2020); Dyrba et al. (2020a); Sagar & Dyrba (2020); Douglas & Farahani (2020); Masood (2020); Jo et al. (2020); Dyrba et al. (2020b); Kohoutová et al. (2020); Jo et al. (2020); Böhle et al. (2019); Eitel et al. (2019a, 2019b); Thomas et al. (2019); Dang & Chaudhury (2019); Xie et al. (2019); Pena et al. (2019); Islam & Zhang (2019); Gotsopoulos et al. (2018); Wang et al. (2018); Yan et al. (2017); Sturm et al. (2016) (58)	EEG/MRI^*^/ fMRI/SPECT/ PET/tau-PET	AD^*^^c^/SZ*^c^*/ PD*^c^*/ASD*^c^*/ MSCS*^i^*/ ER/BA*^e^*	Most frequently used method (after grad-cam); mostly employed in CNN; often used with transfer learning and attention module; also used in benchmarking;
LIME	Lombardi et al. (2022b); Tasci & Tasci (2022); Saboo et al. (2022); Magboo et al. (2022a); Gaur et al. (2022); Magboo et al. (2022b); Sánchez-Hernández et al. (2022); Jahan et al. (2023); Lampe et al. (2022); Giudice et al. (2022); Salih et al. (2021); Lombardi et al. (2021); Shad et al. (2021); Breitenbach et al. (2021); Sidulova et al. (2021); Magesh et al. (2020); Hu et al. (2020); Douglas & Farahani (2020); Ramos et al. (2019); Santana et al. (2019); Pereira et al. (2018b) (21)	EEG/MRI^*^/ fMRI/ DaTSCAN	BA^*^^e^/AD^*^^c^/ ASD*^c^*/PD*^c^*/ BL*^s^*/MCI*^c^*	Unlike SHAP, LIME is not stable; model-agnostic; used both in ML and DL models;
SHAP	Sánchez-Hernández et al. (2022); Lombardi et al. (2022a, 2022b); Paul et al. (2022); Eder et al. (2022); Islam et al. (2022); Hu et al. (2022); Dai et al. (2022b); Brink-Kjaer et al. (2022); Gaur et al. (2022); Allwright et al. (2023); Ran et al. (2022); Shaji et al. (2022); Xu et al. (2022); Kim et al. (2022a); Bloch & Friedrich (2022); Bang et al. (2022); Kannampallil et al. (2022); Pang et al. (2022); Ghosal et al. (2022); Gómez-Ramírez et al. (2022); Qiu et al. (2022); Ben-Zion et al. (2022); Xie et al. (2022); Scheda & Diciotti (2022); Ho et al. (2022); Treaba et al. (2021); Bang et al. (2021b); Bozek et al. (2021); Kim et al. (2021b); Huang et al. (2021); Breitenbach et al. (2021); Bloch & Friedrich (2021b); Danso et al. (2021); Lombardi et al. (2021); Bloch & Friedrich (2021a); Wickramaratne & Mahmud (2021); Turkan & Tek (2021); Salih et al. (2021); Pianpanit et al. (2021); El-Sappagh et al. (2021); Sommer et al. (2021); Ball et al. (2021); Pan et al. (2021); Chun et al. (2020); Li et al. (2020c); Sendi et al. (2020); Suh et al. (2020); Ahmedt-Aristizabal et al. (2020); Saueressig et al. (2020); Al Zoubi et al. (2020); Azevedo et al. (2019) (52)	${\text{EEG/MRI}}^{⋆}$ /fMRI/fNIRS/ SPECT	BA ${⋆}^{e}\text{​}/$ AD ${⋆}^{c}\text{​}/$ $\begin{array}{l} {\text{SZ}}^{c}\text{​}/{\text{PD}}^{c}\text{​}/\\ {\text{MCI}}^{c}\text{​}/\\ {\text{MDD}}^{c}\text{​}/{\text{BT}}^{c} \end{array}$	Third most frequently used method; model-agnostic; used both in ML and DL models; often used in graph networks; also used in ensemble models;

For this meta-analysis, we included studies that applied post hoc explainability methods in the neuroimaging domain. In several cases, a single study employed multiple methods.

*c*: Classification.

*e*: Estimation.

*s*: Segmentation.

*i*: Identification.

* : The most frequent in that group.

AIS: Acute Ischemic Stroke, TSD: Task State Decoding, FC: Functional Connectivity, BA: Brain Age, MSCS: Multiple Sclerosis, BL: Brain Lesion, BT: Brain Tumor, ER: Emotion Recognition.

### 4.2 Perturbation-based methods

#### 4.2.1 Occlusion sensitivity (OS)

Abrol et al. (2020) developed a modified deep ResNet to predict the progression from MCI to AD class and perform other eight combinations of diagnostic and prognostic tasks. The authors used *Occlusion Sensitivity* (Zeiler & Fergus, 2014) to identify the most predictive anatomical regions for the progression. Thirteen brain regions consistently emerged in the top 20 most relevant regions, including the middle temporal gyrus, cerebellum crus 1, precuneus, lingual gyrus, and calcarine. However, the OS method has limitations when considering connectivity among regions (Oh et al., 2019; Yang et al., 2018), because this method only relies on the effects of adjacent neighborhoods.

Plaque morphologies can serve as a guide to understanding AD progression and associated pathophysiology. Consequently, Tang et al. (2019) proposed a CNN model for classifying amyloid-beta (A β) plaques using whole slide images (WSIs). Guided Grad-CAM (Selvaraju et al., 2017) and OS (Zeiler & Fergus, 2014) verified whether the models focused on relevant neuropathological features. Guided Grad-CAM identified salient predictive regions, while OS revealed the interdependence of class-specific features. We summarize more papers that used post hoc approaches in Table 5.

In a graph-based study, Wang et al. (2021a) conducted a study using graphs to capture spatial features in connectome neighborhoods, as opposed to the traditional CNN method using Euclidean neighborhoods. They also performed occlusion (Zeiler & Fergus, 2014) experiments on single-ROI to identify important regions that may contribute to predictions. However, this study focused solely on visualizing spatial feature changes and did not explore changes in temporal features.

#### 4.2.2 Meaningful perturbation

For perturbation-based methods in the medical domain, determining the nature of the perturbation is challenging. Incorrect perturbation can drastically change the input distribution. Uzunova et al. (2019) proposed a method that generates meaningful perturbation of original images. This method uses variational autoencoders (VAE) (Kingma & Welling, 2013) to produce the closest healthy equivalent images. Since the VAE was trained only on healthy subjects, it is assumed to only know the distribution of healthy subjects in the latent space. Hence, during test time, reconstructing from pathological images will generate the nearest healthy equivalent. Figure 4 *Panel B* shows the explanations generated using Grad-CAM (Selvaraju et al., 2017), GBP (Springenberg et al., 2014), and some variants of perturbation techniques for a multi-label classifier designed for retinal OCT images. Compared with other baseline methods, the VAE-based perturbation outperformed constant and blur perturbations. In another study, Kan et al. (2021) applied meaningful perturbation (Fong & Vedaldi, 2017) along with Grad-CAM and GBP and found it useful for generating relatively stable maps. However, this approach can be computationally slower than the other two methods.

### 4.3 Distillation methods

#### 4.3.1 LIME

Magesh et al. (2020) used the VGG16 network pretrained on *ImageNet* to classify Parkinson’s patients from healthy controls, and explained individual predictions using LIME (Ribeiro et al., 2016).

Saboo et al. (2022) developed a DL model for cognition prediction over 5 years after the baseline using clinical and imaging features. LIME (Ribeiro et al., 2016) identified the predictive brain structures (medial temporal lobe, fornix, and corpus callosum) that explain the heterogeneity between cognitively vulnerable and resilient subjects. LIME computed the contributions of each imaging and clinical feature toward the predicted future cognitive score, aligning with some prior reports. However, these attributions were often unstable across runs. Additionally, the rationale for using LIME in both studies (Magesh et al., 2020; Saboo et al., 2022) was not justified and further validation is required.

Gaur et al. (2022) proposed an explanation-driven dual-input CNN-based solution for brain tumor status prediction using MRI scans. SHAP (Lundberg & Lee, 2017) and LIME (Ribeiro et al., 2016) were used to generate explanations, with SHAP capturing the consistency and accuracy in the explanations and LIME capturing the local behavior of the model around the test example. However, the validation of the generated explanations was not provided to support the findings. Explanations were generated in a post hoc manner, and not intended to improve the model’s performance.

Hossain et al. (2023) carried out a study where they utilized different DL architectures and employed the transfer learning approach to detect brain tumors using MRI data. Their approach involved developing an ensemble model by combining the three most successful transfer learning models. For further model validation, they used LIME (Ribeiro et al., 2016) as an explanation tool to show the tumor-affected regions as indicated by the model. Chel and Poh (2025) conducted a comparative evaluation of several well-established CNN-based models, including VGG16, ResNet50, and EfficientNet, focusing on both their accuracy and interpretability in the brain tumor classification task. The authors employed LIME and Grad-CAM to visualize class-discriminative regions. Although all models achieved very high accuracy, the generated visualizations varied substantially across architectures and XAI methods, sometimes highlighting tumor-irrelevant regions. Thus, this study underscores the architecture-specific influence on generated explanations and highlights the limitations of current XAI methods for clinical utility.

#### 4.3.2 SHAP

Ball et al. (2021) utilized three ML techniques to estimate brain age from cortical development. Kernel SHAP (Lundberg & Lee, 2017) was employed to identify explanatory features for prediction errors (brain age delta), revealing consistent explanations across models and previously reported brain development regional patterns. However, there was no observed generic spatial association among individual feature estimates for errors. Despite similar demographics and prediction errors, the importance of features varied significantly between subjects. Additionally, there was no noticeable correlation between brain age delta estimates and cognitive performance.

**Fig. 5.**
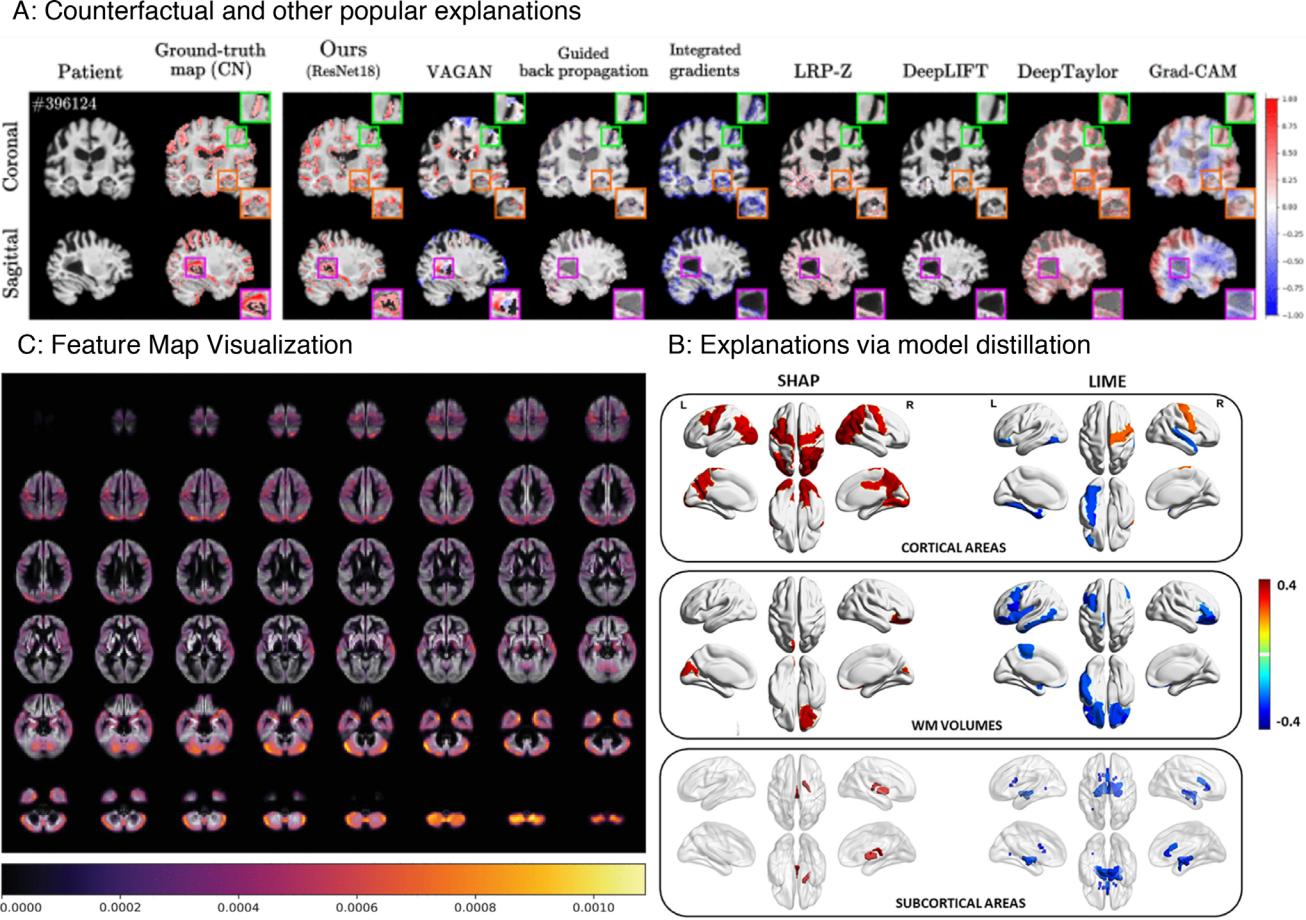
(A) Explanations provided by popular approaches and the counterfactual maps for a sample (subject ID: 396124). The ventricular, cortex, and hippocampus regions are enclosed in purple, green, and orange boxes, respectively. The counterfactual map captured the increased cortical thickness, reduced ventricular, and hippocampal hypertrophy associated with the diagnosis, outperforming visual attribution GAN (VAGAN) maps. While some gradient-based approaches exhibit limited counterfactual reasoning ability, the traditional methods also capture irrelevant regions (Image is adapted from Oh et al. (2022b)). Reprinted with permission from IEEE Transactions on Pattern Analysis and Machine Intelligence. (B) Brain regions that show significant correlations between the attribution scores of the morphological features and the chronological age. Based on SHAP scores, the average thickness, folding, and curvature index statistical attributes calculated based on the precentral gyrus and inferior and lateral occipital cortex were highly correlated with brain age. Additionally, the SHAP scores of the cortical thickness features of both hemispheres showed a significant correlation with age. For LIME, a strong correlation was observed between brain age and the features related to white matter volumes of the opercular and triangular parts of the inferior frontal gyrus and inferior temporal gyrus (Image is adapted from Lombardi et al. (2021)). (C) Area of Influence of the 14^th^ neuron. The highlighted area encompasses parts of the temporal and parietal lobes, as well as a shrinkage of the cerebellum, which is commonly associated with AD progression (Image is adapted from Martinez-Murcia et al. (2019)).

Lombardi et al. (2021) utilized cortical and sub-cortical regions via atlases to extract morphological features from T1-weighted brain scans and developed a brain age prediction model. Multiple training runs with different subsets of the training fold were performed to evaluate the predictive performance and intra-consistency of the explanations. LIME (Ribeiro et al., 2016) and SHAP (Lundberg & Lee, 2017) were employed to analyze the predictive features. Figure 5 *Panel B* demonstrates that SHAP yields greater intra-consistency of feature attribution scores, exhibiting robustness against variations in the training set compared with LIME. Inter-similarity analysis indicates that SHAP values can be more effectively grouped into different age ranges. Moreover, SHAP feature attribution scores exhibited strong correlations with brain age and aligned better with existing brain age literature. However, it is noteworthy that the model was trained on a smaller cohort of samples with a limited age range.

Bhattarai et al. (2024) applied Deep SHAP (Lundberg & Lee, 2017) on a cognition prediction model for MCI/AD mechanisms. The study reported that the method successfully revealed modality-specific critical brain regions and captured complex interactions among those regions. While the initial results show some promises, it has to be scaled to larger and diverse datasets. Additionally, the method still has limitations, such as, increased computational burden over large feature sets and variation of results across multiple runs.

De Bonis et al. (2024) compared the predictive performance and interpretability of two pipelines for brain age prediction: a morphometric feature-based pipeline and a CNN-based pipeline. The key novelty of this work lies in the use of a multisite neuroimaging dataset to enhance model generalizability and in the benchmarking across multiple architectures and training conditions. While the predictive performance of the two pipelines was comparable, SHAP applied to the feature-based pipeline provided consistent explanations, whereas Deep SHAP applied to the CNN-based pipeline showed noticeable variability depending on the background choice.

### 4.4 Counterfactual

Generating counterfactual explanations can be extremely helpful in situations where one would like to know the hypothetical disease patterns that might appear along the course of a longitudinal study. In a similar study, Liu et al. (2021) used conditional convolution to train two simulators in a unified model to inject or remove disease patterns into an MR image to increase and decrease the classifier’s confidence. Validation results revealed a high correlation between the disease patterns injected or removed in the MR image and the ground-truth patterns.

For any graph-based classification tasks, Abrate and Bonchi (2021) devised a method to generate counterfactual graphs for any black-box graph classifier. Using a data-driven or data-oblivious technique, this post hoc approach creates a counterfactual graph that closely resembles the original. An advantage of this method is that the resulting counterfactual graph can be easily understood by humans. Furthermore, the same strategy can be extended to produce LIME-like and global explanations for the “black box.”

Oh et al. (2022b) combined model training, model counterfactual explanation, and model reinforcement into the learn-explain-reinforcement (LEAR) framework. Counterfactual maps guided an attention-based module to refine features for efficient training. After training, to gain deeper insights into the disease, visual counterfactual explanations were generated to create hypothetical abnormalities in the normal input image. Interestingly, the NC to MCI and MCI to AD maps were combined to form the NC to AD map, demonstrating the disorder-specific consistency of the method. Figure 5, *Panel A*, shows the explanations using gradient-based and proposed generative approaches. When compared with the ground-truth control map, the counterfactual explanation detected ventricle enlargement and cortical atrophies, aligning with previous reports.

Recently, diffusion models have gained significant attention due to their ability to produce high-quality images compared with GANs (Dhariwal & Nichol, 2021). Motivated by the need for reliable medical data to scale DL model training and by the potential to provide insights into AD disorders through counterfactual image generation, Liu et al. (2024) employed classifier-free guidance and attention injection with diffusion models to generate counterfactual images consistent with anatomical knowledge, such as structural changes in the hippocampus and ventricles, as well as texture transformations between gray and white matter. Furthermore, the difference images served as explanations for model decisions, and the generated images enhanced the performance of the baseline classifier for AD diagnosis.

To achieve similar end goals, Yeganeh et al. (2025) proposed latent drift for diffusion models to address the distribution shift that arises when a model is pre-trained on natural images and fine-tuned on medical images. Their method can generate medically plausible brain images by correcting the noise distribution. The study demonstrated the efficacy of their approach on multiple neuroimaging AD datasets. While not originally designed to explain prediction models, it can be adapted to produce suitable counterfactual explanations for existing models.

The generative approach is particularly significant as it strongly connects to the concept of counterfactual explanation and aligns with the idea of modular transparency in DL model design. More examples of generative neuroimaging studies can be found under the modular transparency category. We summarize other reviewed papers in Table 6, which leverage the idea of counterfactuals, and image reconstruction using the generative power of DL models.

**Table 6.** Summary of some reviewed papers generating explanations via counterfactuals, image reconstruction, synthesizing image-to-image translation schemes, and leveraging the power of GANs.

Authors & year	Task	Modality	XAI proposal	Baselines XAI
Baumgartner et al. (2018)	Study of AD disease effects	sMRI	Translation-based^a^	CAM, GBP, IG
McClure et al. (2020)	fMRI task decoding	task fMRI	Adversarial training^*^	Gradients, Smoothgrad
Wilms et al. (2021)	Brain Age Regression Brain Sex Classification	sMRI	Attribution map and counterfactual^b^	Gradients, Smoothgrad, and Grad-CAM
Ko et al. (2022)	AD progression, diagnosis MMSE score prediction	sMRI, SNP	Attentive mask via GAN	Grad-CAM
Zia et al. (2022)	Modelling AD Progression BT Segmentation	sMRI	Translation-based^c^	Grad-CAM^d^
Bass et al. (2022)	MMSE score (AD) BA prediction	sMRI	Translation-based^e^	GBP, LRP, Occlusion, IG, CAM variants, etc.
Yu et al. (2023)	Modelling AD Progression	sMRI	Translation-based^f^	CAM, GBP, IG
Matsui et al. (2022)	Brain Activity Decoding	task fMRI^g^	Counterfactual^h^	No post hoc XAI used
Kumar et al. (2022)	MS^l^ Prediction	sMRI	Counterfactual^i^	No post hoc XAI used
Zia et al. (2023)	AD Progression BT Segmentation	sMRI	Counterfactual^j^	CAM, G-CAM, and generative approaches
Alfeo et al. (2023)	EM/SOC^m^ Task Decoding	task-fMRI	Counterfactuals^k^	SHAP
Liu et al. (2024)	AD vs. CN Classification Image Generation	MRI	Counterfactuals and difference maps	No post hoc XAI used
Yeganeh et al. (2025)	Synthetic Image Generation AD vs. CN Classification	MRI, PET, Clinical Data	Counterfactual	No post hoc XAI used

These generative schemes can be considered “joint training” in the taxonomy. Moreover, generative approaches also follow “modular transparency” because the modules in the architecture are designed to achieve specific goals. Based on the use cases, image-to-image translation can also be used as “counterfactual” explanations.

a WGAN: Wasserstein Generative Adversarial Networks.

b Based on invertible normalizing flow-based generative model.

c Visually-Attributed Abnormal-to-Normal Translation GAN.

d Grad-CAM was applied for three flows.

e Extended prior work (Bass et al., 2020) with a regression module.

f MP-GAN: multidirectional perception GAN.

g Brain activation maps.

h StarGAN is modified to generate counterfactuals.

i Image synthesis based on deep conditional generative model.

j Uses cycle-consistency principle in the latent space.

k Used a method, called Boundary Crossing Solo Ratio (BoCSoR).

l MS: Multiple Sclerosis.

m EM/SOC: Emotional/Social.

* Used to ensure robust training.

### 4.5 Feature map visualization

Many studies have employed various methods to examine the flow of information through DL models and understand how concepts emerge from inputs, ultimately leading to the desired outputs. For example, Natekar et al. (2020) employed three distinct DL models to automatically segment gliomas and their intra-tumoral structures. Apart from utilizing Grad-CAM to examine the alterations in spatial attention across network layers, the authors also used activation maximization techniques to determine the visual components captured by the model through each filter. The findings of their analysis demonstrated that the internal layers of the model effectively employed a top–down approach for tumor localization.

Biffi et al. (2020) introduced a ladder variational autoencoder (LVAE), a deep generative model, to learn a hierarchical structure of conditional latent variables for anatomical segmentations. The highest level latent space effectively distinguished clinical conditions in two classification tasks and facilitated visualization through sampling. The model also quantified anatomical shape changes related to these conditions.

Martinez-Murcia et al. (2019) used a deep CNN autoencoder to explore AD by extracting imaging characteristics from GM maps into low-dimensional manifolds. A regression analysis showed that the neurons in the manifold space displayed strong correlations with clinical and neuropsychological test outcomes and diagnoses. A linear decomposition model revealed the brain regions influenced by each manifold coordinate, providing additional insight into structural degeneration and cognitive decline in dementia. Figure 5 *Panel C* depicts the influence of the 14^th^ neuron (most correlated with AD progression).

Parmar et al. (2020) proposed a modified 3D CNN that directly works on the 4D resting-state fMRI data to diagnose different stages of AD (multiclass classification). The study showed the temporal features extracted from the first two convolutional layers as network activation maps. As the temporal features moved from lower to higher layers, discriminative regions of interest progressively revealed a definitive structure.

### 4.6 Intrinsic methods

While numerous neuroimaging studies have utilized post hoc explanation methods, the use of intrinsic interpretability, which involves generating explanations as part of the model training, is still limited. In this section, we discuss several studies that have explored the concept of intrinsic interpretability from different perspectives.

#### 4.6.1 Attention mechanism

As mentioned earlier, one approach to generating intrinsic explanations for a model’s inner workings or decision-making process is by leveraging attention mechanisms. While attention mechanisms have been widely utilized in DL-based neuroimaging studies (Chen et al., 2022d; Lewis et al., 2023; Mahmood et al., 2022; Rahman et al., 2022; Zhang et al., 2021b), the direct use of learnable attention maps as explanations, as proposed by Jetley et al. (2018), is relatively limited. For instance, Chen et al. (2022d) used an attention subnet with a residual module during model training for ASD classification. However, the authors utilized Grad-CAM to generate explanations and gain insights into the disorder. Hu et al. (2021b) used a graph network with multi-head attention for node representation and an attention pooling layer for graph representation to classify ASD from controls. They employed GNNExplainer (Ying et al., 2019), a model-agnostic approach, to generate explanations from the learned model, and compared them with saliency maps and DeepLIFT maps. They identified the top 10 connections based on generated explanations. However, they were not statistically significant between ASD and HC groups, suggesting the need for further experimentation. Similarly, other studies (Lewis et al., 2023; Rahman et al., 2022) have used an attention mechanism to address the issue of vanishing saliency in LSTM networks, improving gradient flow and overall discriminative performance. These studies have employed separate post hoc methods during inference to generate explanations. In many cases, heatmaps generated by Grad-CAM methods are also referred to as attention maps (Gao et al., 2023; Qin et al., 2022), which are technically different from the traditional use of attention modules for dynamically determining the interrelationships among features. In this section, we only consider neuroimaging papers that use trainable attention scores (maps) as explanations for the model’s decisions, similar to the broad idea proposed by Jetley et al. (2018). The papers discussed below serve as examples of intrinsic interpretability through attention mechanisms.

Lian et al. (2022) proposed an attention-guided unified framework using a CNN as task-specific guidance and a multi-branch hybrid network for diagnosis. The motivation was to extract multi-level information at interpersonal, individual, local, and global scales. The first CNN provided guidance through disease attention maps (DAMs) calculated using CAMs, aiding in generating global and individual features. In the second stage, DAMs were passed to different branches to capture patch-level and global-level information for classification. The AD classification task was built from scratch, while the MCI progression prediction model utilized the learned parameters of the AD model. The framework identified parts of the hippocampus, frontal lobe, fusiform gyrus, amygdala, and ventricle as associated with AD or MCI.

In a brain tumor segmentation task, Zhang et al. (2020a) proposed an attention-gated residual U-net, where a series of attention gates and residual modules were integrated within a U-net to learn the informative tumor regions while suppressing other irrelevant regions. While this attention mechanism proves to be useful in the brain tumor segmentation task, the performance gain is not very significant, potentially due to using 2D brain slices to fit into the 2D U-net model.

**Fig. 6.**
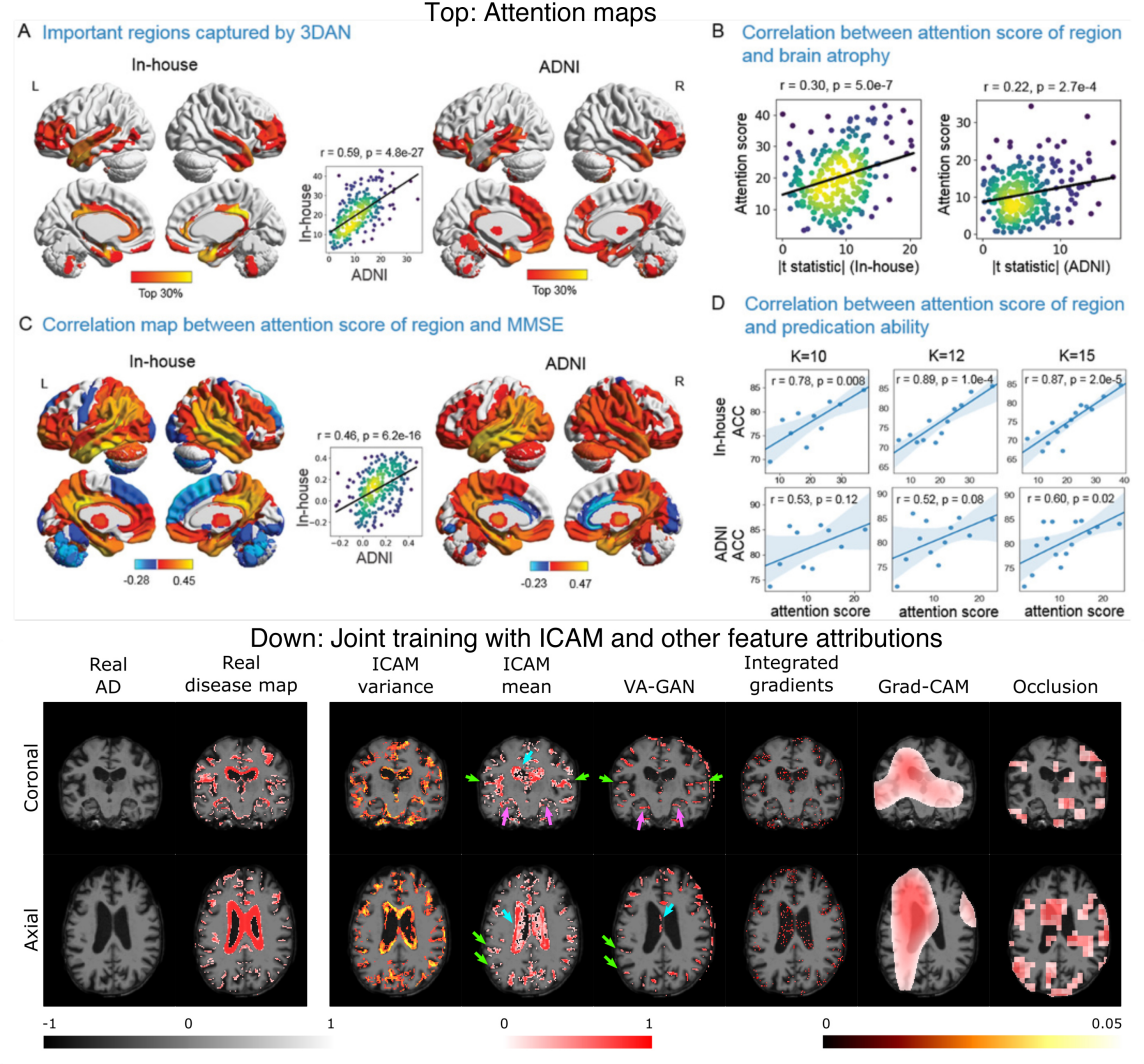
**Top:** (A) Attention maps identify key regions of Alzheimer’s disease (AD): the temporal lobe, hippocampus, parahippocampal gyrus, cingulate gyrus, thalamus, precuneus, insula, amygdala, fusiform gyrus, and medial frontal cortex. (B) Correlation analysis between T-map and attention scores of the regions. (C) Correlation analyses between attention scores and MMSE scores. (D) Correlation between mean attention score of top K regions and classification performance (image is adapted from Jin et al. (2020)). **Down:** Feature attribution maps using Interpretable Classification via Disentangled Representations and Feature Attribution Mapping (ICAM), visual attribution GAN (VA-GAN), and other post hoc methods for AD classification. ICAM effectively captures phenotypic variation in brain structure, detecting the ventricles (blue arrows), cortex (green arrows), and hippocampus (pink arrows) when compared with the ground-truth disease map (image is adapted from Bass et al. (2020)).

Jin et al. (2020) proposed a model that combines ResNet and a 3D attention network (3DAN) to integrate interpretation and classification in a unified framework. The 3DAN establishes a strong association between the model output and the clinical features of AD and MCI. The attention maps were found to be generalizable, reproducible, and neurobiologically plausible. Strong correlations were observed between the attention maps across datasets, the mean attention score and the T-map, the attention score for the regions and the MMSE scores, and the classification accuracy and the mean attention score of K groups of regions. Figure 6 *Top* panel displays various aspects of the analyses.

Ranjbarzadeh et al. (2021) adopted a CNN with distance-wise attention mechanism and an area-expected approach on 4 different MRI modalities for the brain tumor segmentation task. The authors showed the effectiveness of using the attention mechanism as part of the model training to gain more accurate contextual information and achieve improved segmentation accuracy.

Zhang et al. (2023b) proposed a graph convolutional network based on self-attention graph pooling, which combines both structural connections and functional interactions. The authors argue that these graph-based models offer advantages over traditional convolutional and recurrent models in brain imaging because they better align with the inherent nature of functional interactions among spatially distant brain regions. Additionally, the scores of the nodes after the pooling layers provide further guidance for interpreting the results. The self-attention, in association with graph pooling, helps identify the important regions. One caveat, however, is that the results derived from attention mechanisms may not always be biologically or clinically meaningful. For example, Adey et al. (2024) proposed a self-attention-based prototype method, called the self-explaining selective model (SESM), for interpreting DL decisions for the task of sleep stage classification using electroencephalogram (EEG) signals. The generated explanations, that is, prototypical components, were validated against known biomarkers and found to be only partially consistent with the literature: the model primarily extracted time-domain information and often included irrelevant content, limiting clinical utility. The area over the perturbation curve (AOPC) metric was used to objectively evaluate the quality of the top prototypical parts identified by the model. While the AOPC values were considerably higher, the top prototypical explanations produced by multiple attention heads still contained redundant information.

Xu et al. (2024) proposed a novel contrastive graph pooling (CGP) mechanism incorporating a contrastive dual-attention block to extract group-specific biomarkers from noisy fMRI data. The proposed approach employs region-of-interest (ROI)-wise and subject-wise attention, rather than assigning equal importance to all ROIs and subjects, to construct a contrastive graph. Guided by this contrastive graph, the CGP framework demonstrates improved performance over several state-of-the-art approaches across five different datasets and three neurological disorders, while producing more interpretable subgraphs that highlight disease-relevant brain regions. Although the approach enhances interpretability in graph-based neuroimaging models, several of its findings diverge from existing literature and require further validation across diverse cohorts using both quantitative and qualitative evaluation metrics. In another graph-based study, Han et al. (2025) proposed a spatial graph neural network (GNN) model to identify biomarkers associated with motor learning. In addition to achieving higher accuracy than other graph-based models, the authors introduced a specialized explainability pipeline, called the GNN Spatial, Spectral, and Temporal Explainer (GNN-SST-Explainer). The resulting attention maps highlighted meaningful brain regions such as the prefrontal, sensorimotor, and visual cortices. However, these patterns were not consistent across all models and datasets, and in some cases, the attention values were also attributed to non-neural regions despite accurate learning predictions. While the model’s predictive performance and identified biomarkers are promising, the study relies on a small, healthy cohort and lacks quantitative evaluation of the explainability results. The proposed method, therefore, requires validation on larger and clinical populations, multimodal datasets, and with stronger evaluation strategies. Table 7 summarizes more example studies leveraging attention mechanisms.

**Table 7.** Summary of some reviewed papers leveraging interpretability as part of model design: attention mechanisms and joint training are utilized.

Authors & year	Task	Modality	Intrinsic XAI used	Baselines XAI
Li et al. (2020a)	MI^a^ EEG signals decoding	EEG	Sensitivity via Attention	Grad-CAM
Nguyen et al. (2020)	7/Motor/WM^b^ Task state decoding	task fMRI	Temporal Activation via Attention	Grad-CAM
Shi et al. (2020)	Fetal brain age estimation Anomaly detection	T2-w MRI	Attention Activation Maps	GBP
Turkan & Tek (2021)	AD Classification	sMRI	Attention ^*^	Occlusion, Grad-CAM, SHAP, 3D-UCM^c^
Wang et al. (2021c)	Brain Tumor Segmentation	sMRI	Attention ^*^	Grad-CAM
Hu et al. (2021a)	Brain Age Prediction	T1-w MRI	Attention ^*^	Grad-CAM
Kuijs et al. (2021)	AD Classification	1.5T/3T MRI	interpretability aware training ^*^	Grad-CAM
Zhao et al. (2022)	SZ, ASD Classification	rs-fMRI	Attention	LRP
Chen et al. (2022d)	ASD Classification	sMRI	Attention ^*^	Grad-CAM
Qin et al. (2022)	AD Variants Classification	sMRI	Attention ^*^	Grad-CAM
Gao et al. (2023)	MDD Classification	T1-w sMRI	Attention via Grad-CAM	Grad-CAM
Qiao et al. (2023)	Study of dFC^d^ over age	rs-fMRI	Joint Training (SDN-EX^e^)	No post hoc XAI used
Mulyadi et al. (2023)	AD Progression Modelling	sMRI	Joint Prototype Learning	No post hoc XAI used
Adey et al. (2024)	Sleep Stage Classification	EEG	Multihead Attention	No post hoc XAI used
Xu et al. (2024)	PD, AD, ASD Classification	rsfMRI	Attention, CGP^g^	No post hoc XAI used
Han et al. (2025)	MLS^f^ Prediction	EEG	NA^h^, NEM^i^, STA^j^	No post hoc XAI used

It should be noted that the attention mechanisms employed in these studies are not necessarily intended to provide explanations but are always motivated to improve the predictive capacity and expressiveness of the model.

a MI: Motor Imagery.

b WM: Working Memory.

c UCM: Ultrametric Contour Map.

d dFC: Dynamic Functional Connectivity.

e SDN-EX: sparse deep network with explainability.

f MLS: Motor Learning State Prediction.

g CGP: Contrastive Graph Pooling.

h NA: Node Attention.

i NEM: Node and edge masking.

j STA: spectral and temporal ablation.

* not used to generate explanations.

#### 4.6.2 Joint training

The idea of joint training is strongly tied to the notion of optimizing the model both for performance and interpretability, without relying on any post hoc XAI methods. Zhu et al. (2022) employed a similar approach, combining interpretable feature learning and dynamic graph learning modules into the Graph Convolutional Network (GCN) module. The authors optimized both components jointly to enhance both diagnosis and interpretability. Feature summarization was performed using gray matter volumes from Regions of Interest (ROIs). The interpretability of the diagnosis was evaluated based on the learned weights of each region. The top regions displayed superior predictive performance compared with other feature selection methods. Furthermore, the proposed method consistently identified the following regions as relevant for AD: middle temporal gyrus right, hippocampal formation left, precuneus left, and uncus left.

In another study, Bass et al. (2020) introduced ICAM (Interpretable Classification via Disentangled Representations and Feature Attribution Mapping), a generative approach, to learn a shared class-relevant attribute latent space suitable for both classification and feature attribution. The visual demonstration is shown in Figure 6 *Down* panel. ICAM’s latent attribute space exhibits superior discriminative power, and the attribution maps show higher correlations with ground-truth disease maps compared with other approaches. Another study proposed a deep generative model for transparent visualization of the classification space (Biffi et al., 2020). We summarize reviews of other papers that used attention mechanisms and joint training in Table 7.

Traditionally, studies have used regression models for brain age prediction to predict the chronological age of a healthy population. Anatomical explanations provided by such studies, using post hoc methods, are inconclusive due to the substantial variation in these explanations based on the choice of model, training algorithms, input perturbations, and other parameters. In order to address the lack of interpretability at the model level and explainability at the instance level in the ∇-Age measure, Sihag et al. (2023) implemented a graph neural network (GNN) called the coVariance Neural Network (VNN) for brain age prediction, using cortical thickness features. The eigenvectors of the anatomical covariance matrix highlight the specific brain regions that contribute to the elevated brain age gap in Alzheimer’s disease (AD).

In a graph-based analysis, Li et al. (2021) proposed an interpretable graph neural network that incorporates ROI-selection pooling layers. This network learns which regions are salient for making predictions in an end-to-end learning paradigm. During model training, a tunable regularization parameter is used to encourage the model to learn individual-level and group-level salient patterns. The discovered patterns are co-aligned with the existing literature but lack rigorous quantitative evaluation.

In a recent study by Azevedo et al. (2022), the authors employed GNN and TCNs to learn both structural associations and temporal dynamics. They then examined the assignment matrices from the initial hierarchical spatial pooling layer to extract additional neurobiological insights. However, further investigation is needed to determine the relevance of these regions at the phenotypical level. We summarize some other papers using graph-based approaches in Table 8.

**Table 8.** Summary of some selected papers using explainable graph neural networks for neuroimaging data.

Authors & year	Task	Modality	XAI method
Leming & Suckling (2020)	Gender Classification	rs-fMRI and task-fMRI	Occlusion, Grad-CAM
Saueressig et al. (2020)	Brain Tumor Segmentation	3D sMRI	SHAP
Lanciano et al. (2020)	ASD^b^ Classification	fMRI	self-explanatory
Kim & Ye (2020)	Sex Classification	rs-fMRI	Grad-CAM
Azcona et al. (2020)	ADD^a^ classification	T1w-MRI	Grad-CAM
Qu et al. (2021)	WRAT^f^ score classification	multiple fMRI paradigms	Occlusion
Zhang et al. (2021d)	cognitive state decoding	task-fMRI	GBP
Kim et al. (2021a)	Gender Classification Task Decoding	rest/task fMRI	spatio-temporal attention
Wein et al. (2021)	investigate spatiotemporal relationship	DTI and rest fMRI	causal connectivity
Liu et al. (2022b)	AD, MCI, ASD Diagnoses	rs-fMRI	Grad-CAM
Zhou et al. (2022)	AD Classification	sMRI and PET paradigms	Grad-CAM
Wadhera & Mahmud (2022)	ASD vs. NC^c^ Classification	EEG	IG
Ghosal et al. (2022)	SZ Classification	fMRI paradigms and SNP^d^	BFS^e^, SHAP
Ye et al. (2023)	Brain State Decoding	rs/task-fMRI	BrainNetX^g^
Xu et al. (2024)	PD, AD, ASD Classification	rsfMRI	Attention, CGP^i^
Han et al. (2025)	MLS^h^ Prediction	EEG	NA^j^, NEM^k^, STA^l^

a ADD: Alzheimer’s disease dementia.

b ASD: autism spectrum disorder.

c NC: normal controls.

d SNP: single nucleotide polymorphism data.

e BFS: Bayesian feature selection.

f WRAT: Wide Range Achievement Test.

g an explanation tool to annotate task-relevant regions.

h MLS: motor learning state prediction.

i CGP: contrastive graph pooling.

j NA: node attention.

k NEM: node and edge masking.

l STA: spectral and temporal ablation.

#### 4.6.3 Modular transparency

**Fig. 7.**
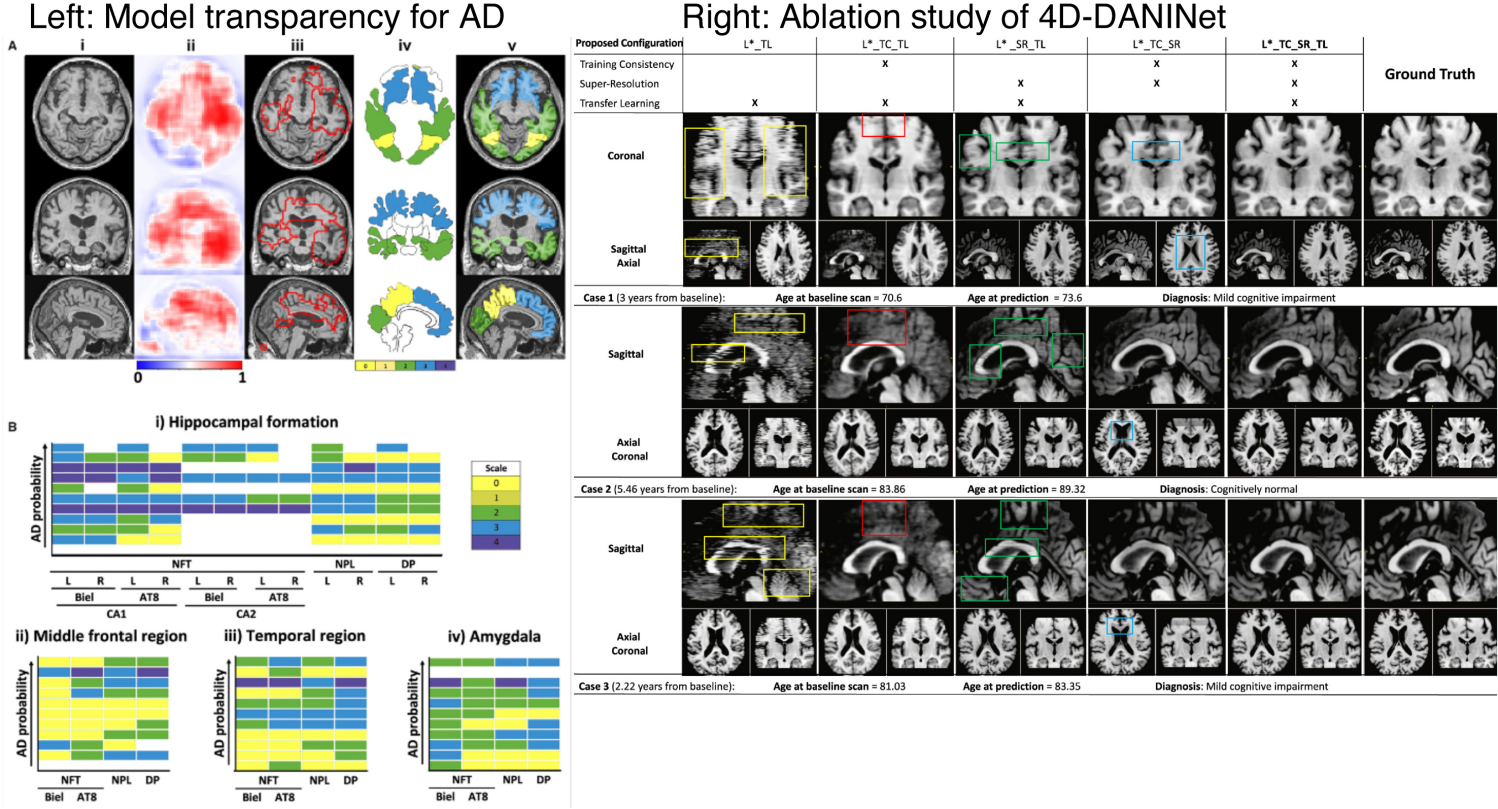
**Left:** (A) Overlap of the model’s predictions with the neuropathology in an AD subject, showing *(i)* MRI scans at different visual planes, *(ii)* predicted disease probability maps, *(iii)* thresholded probability maps overlapped with the MRI scans, *(iv)* segmented brain masks with color-coding indicating different pathology levels, and *(v)* an overlay of MRI scan, thresholded probability maps, and color-coded pathology levels. (B) Density of neurofibrillary tangles (NFT), diffuse senile (DP), and neuritic (NPL) or compacted senile plaques in each brain region, with average probabilities of the identified regions consistently correlated with a high grade of amyloid-β and tau in different brain regions. Biel = Bielschowsky stain; L = left; R = right (image is adapted from Qiu et al. (2020)). **Right:** Ablation study of the proposed full configuration ${\text{L}}^{\text{*}}$_TC_SR_TLI. The L_TL configuration lacks 3D consistency constraints. When training does not converge, it results in artifacts appearing in sagittal and coronal axes (yellow boxes). If super-resolution is not used, anatomical details are often not visible (red boxes). If SR only model is used without TC, unrealistic images may appear (green boxes). If transfer learning is omitted during test time, it may cause inaccurate morphology (configuration L_TC_SR, blue boxes) (image is adapted from Ravi et al. (2022)).

As defined earlier, modular transparency does not provide explanations for a model’s decisions. However, it provides an intuition of how the model components are interconnected to accomplish the task intuitively. To incorporate similar ideas, Qiu et al. (2020) utilized multimodal inputs such as MRI and clinical features for an interpretable framework. The framework improves AD diagnosis and identifies disease-specific neuroimaging signatures. MRI sub-volumes are passed to a CNN, and patient-specific probability maps of the brain are generated. High-risk voxels are passed to a fully connected network for classification. The high-probability brain regions were associated with the locations and frequencies of amyloid-β and tau pathologies. Furthermore, 11 neurologists conducted diagnoses based on the same inputs, and their performance was compared with the model’s performance. Figure 7 *Left* panel demonstrates how the disease probability maps correspond to the post-mortem findings of neuropathology examinations. Mahmood et al. (2022, 2023) proposed an intrinsically interpretable DL model by incorporating modular transparency into its design. The model exposes itself through a directed graph layer, allowing for the estimation of the new set of directed connectivity (DC) that it learns during training. This estimated DC has potential advantages over traditional functional connectivity due to its dynamic and task-dependent nature. Additionally, the study can be categorized as an example of a trainable attention mechanism, as it uses temporal attention to generate a connectivity matrix based on local estimates.

Modular Transparency via Generative Adversarial Networks (GANs) Neuroimaging studies that focus on DL-based image generation typically utilize multiple variants of the generative adversarial network (GAN). Refer to the review by Wang et al. (2023b) on generative studies in neuroimaging, which include image reconstruction, image-to-image translation, image synthesis, and counterfactual explanations.

As mentioned earlier, generative approaches are not always intended to explain the decisions of a predictive model. However, if generative models are carefully designed, they can provide useful counterfactual explanations and novel insights. For instance, Bowles et al. (2018) proposed a generative model that can generate realistic brain atrophy images based on the original MR image of a subject. This model was able to track the progression of AD and showed significant correlations with images obtained from a longitudinal study. Consequently, it effectively demonstrates the ability to introduce and remove AD patterns.

Ravi et al. (2022) proposed a 4D-Degenerative Adversarial NeuroImage Net (4D-DANINet) for generating realistic 3D brain images over time, reflecting disease stage and age. The main components of the DANINet are (1) The *conditional deep encoder* combines an encoder and a generator to embed each slice to a latent space and generate samples conditioned on diagnosis and age. (2) The *discriminator networks* consist of two discriminators, one for distinguishing between real and simulated brain images and another adversarially trained with the encoder for ensuring smooth temporal progression. The generator generates realistic synthetic images to fool the discriminator. (3) *biological constraints* capture smooth intensity changes. (4) *profile weight functions* dynamically determine appropriate weights for the losses. Figure 7 *Right* panel visualizes the roles of different components in the proposed framework.

Jung et al. (2023) proposed a conditional Generative Adversarial Network (cGAN) supplemented with a 3D discriminator to generate realistic 3D images depicting the stages of AD progression. This novel approach addresses the issue of spatial artifacts commonly present in generated images caused by previous methods utilizing either 2D brain slices or downsampled 3D images. Additionally, they introduced an identity loss mechanism to preserve the fundamental characteristics of the patients.

Pombo et al. (2023) developed a new 3D generative model for generating counterfactual data by injecting biologically plausible and label-guided volumetric brain images. The main objective is to ensure model fidelity, which is often affected by data imbalance and distributional instability. The synthesized image is created by morphing another image in a counterfactual manner while preserving its key identity features. Unlike the previous work by Ravi et al. (2022), this study focuses on unpaired image-to-image translation to generate synthetic images for under-represented subgroups. We summarize additional studies that are aligned with the modular transparency design principle based on GANs in Table 6.

## 5 Discussion

**Fig. 8.**
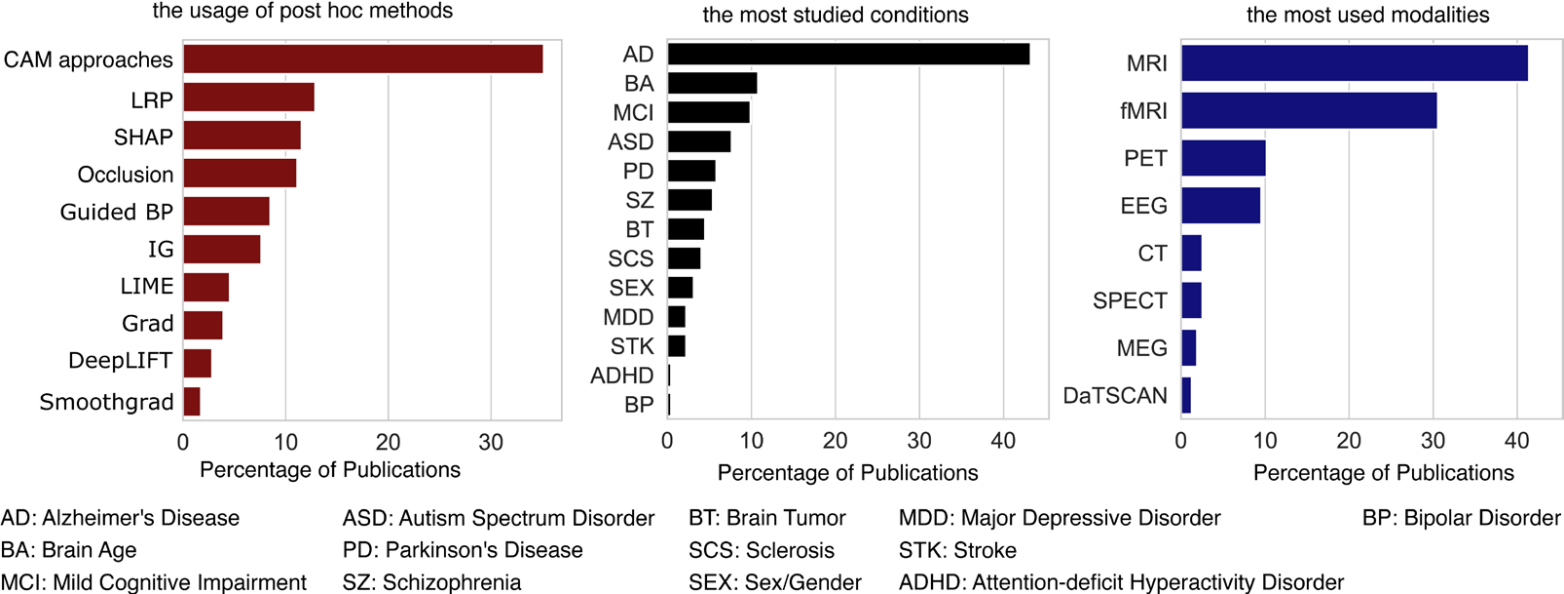
This trend is an approximation and only based on the reviewed papers. Left: The use of post hoc methods over the years. Middle: The area of studies covered in this collection of papers. Right: The focus of the imaging modalities centers around MRI (T1, T2, Flair, Diffision) and fMRI.

We reviewed a total of 122 papers from the XAI domain to introduce XAI methods, metrics, and available toolkits that practitioners can readily use. Then, we discussed 409 neuroimaging papers that primarily utilize DL technology and interpretability approaches. The reviewed neuroimaging papers were categorized based on XAI methods. We propose an updated taxonomy that builds upon the taxonomy proposed by Ras et al. (2022). Specifically, we added “counterfactual explanations,” “example-based explanations,” and “concept-based explanations” as important branches of interpretability, each with their own objectives and techniques. We observed that the majority of neuroimaging studies using XAI employ supervised models, such as classification and regression models, which may be attributed to the availability of XAI resources in supervised settings (Crabbé & van der Schaar, 2022b). Notably, the focused study areas and modalities vary significantly. The MRI modality receives the most attention, possibly due to the outstanding performance of DL models in the computer vision domain and the abundance of resources available. This is further supported by the extensive use of CNNs in brain imaging. Among the reviewed papers, AD is the most widely studied area. However, it is worth noting that most studies mainly employ post hoc methods. Many studies have also employed the idea of intrinsic interpretability via attention mechanisms, joint training, and generative models. However, models with the capacity to simultaneously generate predictions and explanations are still limited. To explore the most focused imaging modalities, study areas, and the usage of post hoc methods, we also conducted an exploratory analysis of the papers collected in Table 5. For more detailed information, refer to Figure 8.

We note that the usage frequency or popularity of XAI methods, as shown in Figure 8, does not necessarily provide guidance for future neuroimaging practitioners regarding the methods’ potential value. Often, these methods were selected in arbitrary or heuristic manner rather than based on their intrinsic value. For example, CAM-based approaches have been heavily used in recent years despite their significant limitations, while IG has seen relatively low usage despite its strong theoretical foundations, as discussed in Section 5.3. Moreover, the de facto use of post hoc methods is functionally limited and may only point to a subset of features (Baumgartner et al., 2018; Zia et al., 2022). For example, LRP and GBP can identify homogeneous brain regions such as the hippocampus but are unable to identify heterogeneous regions such as cortical folds (Böhle et al., 2019; Eitel et al., 2019a). Nonetheless, one of the main reasons studies have used post hoc extensively is because they are easy to use, available as open source (Van der Velden et al., 2022), and usually do not impose any specialized requirements. For a detailed discussion on the relative strengths and weaknesses of post hoc methods, refer to Section 5.3.

### 5.1 Feature extraction-based versus DL approaches for neuroimaging analysis

While both feature extraction-based approaches and DL approaches have been widely used to model observational data, deeper insights into their diverse implications are often unclear. In this section, we discuss the pros and cons of these two approaches from diverse perspectives so that practitioners can make an informed choice of the models that best fit their study goals.

As pointed out earlier, DL models are capable of automatically learning complex, hierarchical, and potentially domain-adaptive features with minimal human intervention (Qiu et al., 2016), making the machines independent of costly domain knowledge (Najafabadi et al., 2015). On the contrary, feature extraction-based approaches, commonly used with classical ML models, require domain expertise and a trial-and-error process to hand-engineer useful features, incurring a considerable amount of human effort (Alzubaidi et al., 2021). ML models may also compromise performance when tasks involve complex high-dimensional data. Also, features learned by DL models are arguably richer in information than hand-engineered features due to the diverse inductive biases incorporated into the models.

While DL is considered as a subset of ML, it provides more flexibility than many traditional ML algorithms in terms of architecture choice, input and output (i.e., regardless of annotation) structure, and capacity to learn multidimensional (e.g., spatial and temporal) features. In particular, we can build DL models for both structured (e.g., tabular data) and unstructured data (e.g., images, text, audio, video) and for supervised and unsupervised learning scenarios, whereas traditional ML models mostly learn on structured data. DL models usually require massive amounts of data to learn meaningful relationships within the data and usually offer superior performance, whereas traditional ML algorithms are not developed to deal with big data (Qiu et al., 2016). However, the challenge is that DL approaches, if trained on small amounts of data, may incur inaccurate results and high variance due to overfitting. On the contrary, for high-dimensional datasets, the performance of traditional ML algorithms deteriorates without reliable feature extraction. The computational load for DL models is very high compared with traditional ML models because DL approaches require developing an intended model for the target task based on iterative experimentation with multiple design parameters and resource constraints. Compared with traditional ML, DL offers more effective and flexible mechanisms for transferring knowledge across domains or tasks, although it still faces several challenges. While many traditional ML models are highly scalable (e.g., linear regression, logistic regression), models such as k-NN and SVM with nonlinear kernels face scalability challenges due to their computational and memory demands, especially when applied to large or high-dimensional datasets. Therefore, many traditional ML approaches often require decomposing the problem into smaller problems and ensembling the solutions together. However, DL is highly scalable and larger problems can be solved end-to-end. Feature extraction-based approaches are usually more interpretable than DL models though this is not always the case. Refer to the first recommendation from Section 5.4 for more insights. As an example case study, Barnard et al. (2025) introduce StateViewer, a powerful ML framework that leverages FDG-PET scans to support clinical decision making. StateViewer employs a neighbor-matching algorithm (k-nearest neighbors) to detect various neurodegenerative conditions and generates interpretable relevance maps to justify its predictions. In a radiologic reader study, the authors demonstrated that the tool can enhance diagnostic accuracy in clinical settings. However, the study’s generalizability is limited due to the geographically restricted participant cohort. While classical approaches are simpler and more inherently interpretable, DL models are more powerful but require robust XAI methods to achieve comparable clinical interpretability. We also note that the explainability of DL models is expected to improve in the coming years as the XAI field is growing fast. Overall, the choice between DL approaches and feature extraction-based predictive methods depends on the specific task at hand, data availability, performance requirements, computational and resource constraints, the desired level of interpretability, the feasibility of incorporating human effort, and many other factors.

### 5.2 Critical analysis and research gaps

In this section, we provide a set of critical observations and associated research gaps based on our review of general XAI literature and relevant neuroimaging studies. In Table 9, we also provide a set of critical comments and opinions from some seminal XAI papers that we deem useful for the neuroimaging community for their future XAI practices. Our observations and associated research gaps are as follows:

**Table 9.** Common opinions, commentaries, suggestions on XAI methods from some seminal XAI papers.

Reference	Key opinions and takeaways
Doshi-Velez & Kim (2017)	suggested performing evaluations using one of the proposed approaches as deemed consistent with claims and encouraged to use common taxonomy.
Adebayo et al. (2018)	demonstrated that Guided Grad-CAM, GBP are insensitive to model parameters and the data generation process and hence may not provide useful explanations.
Lipton (2018)	argued that linear models are not always more interpretable; claims about interpretability must be justified. Accuracy and transparency should be judiciously balanced.
Rudin (2019)	argued that explainable ML methods (mainly post hoc) are not faithful, that is, the explanations do not correspond to what the model computes.
Rudin & Radin (2019)	argued that the accuracy–interpretability tradeoff is a fallacy. We can design interpretable models both in ML and DL without compromising accuracy.
Kindermans et al. (2019)	showed that many saliency methods (Bach et al., 2015; Montavon et al., 2017; Shrikumar et al., 2016; Sundararajan et al., 2017) fail to satisfy input invariance property and thus do not mirror the sensitivity of the model to input transformation.
Ghorbani et al. (2019)	showed that saliency methods (Shrikumar et al., 2017; Simonyan et al., 2013; Sundararajan et al., 2017) can generate various interpretations for visually similar adversarial inputs despite having the same predicted label.
Páez (2019)	encouraged that our focus should be on pragmatic conditions instead of factivity conditions taking cues from background knowledge, and interests of end-users.
Loyola-Gonzalez (2019)	showed that both black- and white-box models have advantages. However, experts need to understand the data, the problem to solve, and the way output to be presented. Statistical evaluation of expert opinions may increase a model’s effectiveness.
Tomsett et al. (2020)	showed that saliency metrics used to assess saliency maps are statistically unreliable and inconsistent. So, the comparative rankings of methods are not reliable.
Adebayo et al. (2020)	showed that some post hoc methods can identify spatial spurious signals, but not mislabeled examples. Modified BP methods cannot detect model contamination. Attributions of out-of-distribution inputs may be visually misleading.
Lakkaraju & Bastani (2020)	demonstrated that the user trust in black-box models can be manipulated via misleading but high-fidelity explanations.
Slack et al. (2020)	demonstrated that explanations relying on input perturbations, such as LIME and SHAP, are prone to adversarial attacks and cannot uncover underlying biases.
Loi et al. (2021)	proposed a paradigm for designing models that incorporates domain expertise, user understanding, and interests to establish justifiable and attainable goals.
Ghassemi et al. (2021)	argued that current XAI approaches may be useful globally, but they fall short in enabling individual-level trust, transparency, and bias mitigation. Extensive model validation is recommended.
Krishna et al. (2022)	formalized the disagreement of different post hoc methods and argued that we need more principled metrics for evaluation.
Dai et al. (2022a)	showed with an evaluation framework that post hoc methods (Lundberg & Lee, 2017; Sundararajan et al., 2017) exhibit disparities for over 50% of dataset–model combinations.
Adebayo et al. (2022)	demonstrated that post hoc explanations cannot reveal unknown spurious correlations. Feature attribution methods may erroneously indicate the model’s reliance on the spurious signal.
Hatherley et al. (2022)	supported interpretability over accuracy for medical AI systems, asserting that sacrificing interpretability for accuracy might reduce AI advantages and harm patients.
Hedström et al. (2023a)	proposed a principled approach to reliably evaluate the evaluation metrics to deal with the disagreement problem of post hoc methods.
Herm et al. (2023)	showed that the tradeoff between performance and explainability is an oversimplification. Data complexity, user biases, and explanation types also come into play.

Some of these papers have a greater influence on the ongoing progress of XAI.

*(a) Evidence shows that XAI practices in neuroimaging lack clear objectives for interpretability:* In the literature, there are many different interpretations of what it means for a model to be interpretable. However, studies often claim, without proper justification, that their models are interpretable (Lipton, 2018). Lipton (2018) argues that authors should explain the specific interpretability objectives they aimed for and provide evidence that their models achieved those objectives. However, the current trend of interpretability practices in neuroimaging does not consider the needs of end users or clinical requirements. However, practitioners can choose from various evaluation options proposed by Doshi-Velez and Kim, depending on the available resources and the goals of the task.

*(b) There is a lack of a reliable validation approach:* Currently, there is a clear lack of reliable methods to quantitatively evaluate and theoretically analyze the results provided by XAI approaches. Often, neuroimaging studies mainly rely on intuition, hypotheses, and previous findings to support their explanations (Abrol et al., 2020; Ball et al., 2021; Biffi et al., 2020; Chen et al., 2022d; Dyrba et al., 2020a; Eitel et al., 2019b; Essemlali et al., 2020; Kan et al., 2021; Leming et al., 2020; Li et al., 2021; Lian et al., 2022; Lin et al., 2022a; Magesh et al., 2020; Martinez-Murcia et al., 2019; Oh et al., 2019; Tang et al., 2019; Wang et al., 2021a; Yan et al., 2017; Yang et al., 2018; Zhang et al., 2021b; Zhao et al., 2022). However, this subjective validation approach is prone to bias and can lead to incorrect and not useful conclusions. While some metrics (see Section 3.2) and toolkits (see Section 3.3) exist that can evaluate the explanations quantitatively, we still need well-principled evaluation methods (Hedström et al., 2023a) that can reliably evaluate the explanations before the models are used in clinical settings or the insights are considered as scientific advancements. Rahman et al. (2022) used a quantitative framework, similar to Hooker et al. (2019), to demonstrate the model-identified salient regions are highly predictive, but the metric relies on modified input data. While quantitative evaluation of explanations, if theoretically justified, may indicate reliability of the models, we still need to validate models from different standpoints. For example, Qiu et al. (2020) validated their model using data from independent cohorts, neuropathological findings, and expert-level evaluation. Recently, Deshpande et al. (2024) suggested a consensus across multiple XAI methods to rule out false positives. However, we argue for an extra layer of caution and further expert evaluation before drawing conclusions. For more information on reliable quantitative metrics, we refer to the reports by Tomsett et al. (2020) in Table 9.

*(c) Choice of interpretability method follows no principled approach:* The choice of post hoc XAI methods is generally made in an arbitrary or heuristic manner rather than following a principled framework. Also, the explanations produced by these post hoc methods are very generic and vary significantly based on the selected XAI method, model choice, performance, parameter initializations, and other parameters. This variation leads to a well-known problem in XAI literature called the “disagreement problem” (Krishna et al., 2022), wherein different explanation methods may yield conflicting explanations for the same model prediction (Chel & Poh, 2025). Contrasting perspectives were also noted across studies employing post hoc methods. Wang et al. (2025) reported that IG more effectively captured AD-related brain alterations than GBP, Guided Grad-CAM, and LRP, whereas other investigations (Leonardsen et al., 2024; Hofmann et al., 2025) demonstrated superior performance of LRP over other gradient-based approaches. Although Grad-CAM has been widely used in neuroimaging, De Bonis et al. (2024) found that when applied to CNN-based models, it generated visually coarse maps that require further investigation to assess their potential clinical utility. Moreover, the observed near-zero correlations between SHAP and Grad-CAM demonstrate that these two methods capture different aspects of the underlying model’s decision-making process. These inconsistencies highlight that, without rigorous validation, relying on such explanations for clinical decision making or treating the generated findings as novel scientific insights may be misleading (Lipton, 2018).

*(d) Popular XAI methods in neuroimaging have unresolved issues:* Despite their widespread use within the neuroimaging community (see Fig. 8), post hoc XAI methods are not reliable. Instead of providing a unified understanding of how a model functions, current post hoc XAI methods focus on different aspects of the model’s behavior, often producing divergent feature attributions (Han et al., 2022; Krishna et al., 2022). Numerous studies have highlighted pitfalls in post hoc interpretability methods (Chan, 2021; John-Mathews, 2021; Lakkaraju & Bastani, 2020; Laugel et al., 2019a, 2019b, 2019c; Leslie, 2019; Loi et al., 2021; Loyola-Gonzalez, 2019; Rudin & Radin, 2019; Rudin, 2019; Slack et al., 2020), raising concerns about the unreliable behavior of many methods, as reported in several studies (Adebayo et al., 2018, 2022; Ghorbani et al., 2019; Kindermans et al., 2019). In neuroimaging, anatomical explanations generated using post hoc methods are often considered inconclusive for various reasons, including model choice, training algorithm, input perturbations, and more (Sihag et al., 2023). Often, explanations may not reveal statistically significant group differences, as observed in an ASD classification task (Hu et al., 2021b). For more information, refer to Section 5.3 for a detailed discussion on the utility of post hoc XAI methods.

*(e) Susceptibility to adversarial attacks:* Interpretations based on feature importance maps, such as DeepLIFT, IG, and influence functions, are vulnerable to adversarial attacks (Ghorbani et al., 2019). Similar vulnerabilities have been reported for LIME and SHAP (see Table 9). In neuroimaging, this vulnerability has practical implications. First, MRI data are often noisy due to equipment variation or human error, which can inadvertently act as adversarial perturbations and produce misleading explanations. Second, neuroimaging studies often pool data from multiple sites and rely on model sharing or collaborative training (e.g., federated learning). While these practices improve generalizability and facilitate reproducibility, they also incur increased vulnerabilities: adversaries can inject perturbed data, manipulate shared model updates, or exploit site-specific heterogeneity, distorting explanations and impacting model accuracy (Cheng & Ji, 2020). Third, adversarial perturbations may reveal sensitive information about participants when models are shared or deployed (Gupta et al., 2021b), especially if adversaries have access to individual scans, which may have legal consequences. Finally, misleading explanations in critical applications can misguide clinical interpretation or scientific conclusions. Therefore, neuroimaging practitioners must be aware that explanations are not inherently reliable and should be validated carefully before drawing biological or clinical insights.

*(f) Human-centered XAI design:* In a systematic review, Chen et al. (2022a) found that current XAI practices in medical imaging do not include end-user involvement in the design. Only a few studies have conducted empirical user evaluations to validate their claims (Chen et al., 2022a). Similarly, human-centered evaluations of the generated explanations in neuroimaging studies are rare. We found only one study by Qiu et al. (2020) that conducted both neuropathological and neurologist-level evaluations. In their review, Viswan et al. (2024) also did not find any AD study where experts from the medical domain validated the explanations of the models’ decisions. Recently, there have been calls for a comprehensive human-centered approach that promotes engagement from multiple stakeholders and disciplines in designing trustworthy AI systems (Dey et al., 2022; Reyes et al., 2020; Schoenherr et al., 2023). One of the goals of XAI is to enhance our understanding of the application domain. However, since XAI is still an actively growing and unreliable field, there is a trade-off between domain requirements and technological commitment. Several studies have proposed guidelines on how to involve humans such as data scientists, clinical researchers, and clinicians in the design of responsible AI systems (Chen et al., 2022a; Dey et al., 2022; Ehsan & Riedl, 2020; Leslie, 2019). To foster ML models in healthcare, Imrie et al. (2023) argued that different stakeholders have their respective requirements for interacting with the XAI system. The authors also pointed out how stakeholders, such as model developers, medical researchers, regulators, clinicians, and patients, may benefit from specific types of post hoc explanations and thus can achieve their objectives. The authors’ perspective also provides an interpretability toolbox to facilitate easy adoption of their suggestions. This human–XAI interaction allows human understanding to evolve alongside technological advancements. For example, Cai et al. (2019) developed a collaborative medical image retrieval system that includes a refinement tool for pathologists to interact with the black box based on their specific needs. This enables users to have a better understanding of the system’s behavior and facilitates future improvements. Schoonderwoerd et al. (2021) conducted a case study on a clinical decision support system for child health, illustrating the incorporation of humans into the design of XAI systems, and providing general solutions for human-centered XAI design. We also refer to the guideline recently proposed by the FUTURE-AI consortium, which brings together 117 experts from 50 countries (Lekadir et al., 2025). This guideline outlines 30 widely accepted recommendations for trustworthy healthcare AI, focusing on key principles such as fairness, universality, traceability, usability, robustness, and explainability. It further emphasizes the importance of multi-stakeholder collaboration, continuous risk assessment and mitigation, and systematic bias detection to ensure the trustworthiness of AI systems.

*(g) The use of intrinsic interpretability is still evolving:* While the use of intrinsic methods in neuroimaging is still evolving (see Table 7), these methods can be strong candidates for future neuroimaging practitioners because they provide explanations with higher fidelity. By considering both diagnosis and interpretability during model training, intrinsic approaches provide more faithful explanations than post hoc methods (Bass et al., 2020; Zhu et al., 2022), mainly because they are algorithmically transparent and their explanations are tightly coupled with model predictions (Bowles et al., 2018; Mahmood et al., 2022; Qiu et al., 2020; Ravi et al., 2022). We also note that post hoc methods, although less faithful, can still offer useful insights for exploratory analysis and model debugging (Leonardsen et al., 2024).

Bass et al. (2020) demonstrated that jointly training a model can simultaneously offer discriminative power and highly correlated disease maps compared with post hoc approaches. The use of attention may allow us to extract multi-level information (Lian et al., 2022), improve accuracy, and gain more contextual information (Ranjbarzadeh et al., 2021). Jin et al. (2020) also reported generalizable, reproducible, and neurobiologically meaningful attention maps for AD classification. For optimal performance, however, the choice of model is expected to be consistent with the inputs (Zhang et al., 2020a). For example, Zhang et al. (2023b) argued for self-attention-based graph models over convolutional and recurrent models because graphs can capture both structural connections and functional interactions among distant brain regions. Many other neuroimaging studies (Azevedo et al., 2022; Li et al., 2021) have also developed intrinsically interpretable graph-based models. Nonetheless, explanations derived from intrinsic interpretability methods may still not be reliable for clinical value and neurobiological insights and are still subject to rigorous validation. While intrinsic methods are time consuming, demand expert involvement to solve more complex problems, and may need additional computing power (Ras et al., 2022), we need to devise novel ways to incorporate interpretability into model development. For example, Böhle et al. (2022) demonstrated that applying B-cos transform instead of a linear transform, and thus constraining weight–input alignment during model training, inherently makes a DL model directly explainable.

*(h) The focus is usually on interpretation rather than model debugging:* There is an assumption that highly accurate models lead to better interpretability. However, predictive accuracy does not guarantee interpretability or correct reliance on hidden features (Qiu et al., 2020). DL models can rely on various hidden factors in the data to model the input–output relationship, regardless of their intelligibility (Hinton, 2018). Therefore, a highly accurate model may lack interpretations that are understandable for humans or might be irrelevant to the problem the model was conceived for (Lapuschkin et al., 2019). In particular, it is possible that AI models are consistently distracted by implausible image regions, yet still perform well on a test set (Ghassemi et al., 2021).

We argue that interpretability can be a tool for model debugging to interrogate models to identify potential failure modes and the biases they may exhibit, thus increasing model performance and reliability. It is also useful to inspect whether the model is learning some aspects that are coherent with the domain for scientific consistency (Roscher et al., 2020). To the best of our knowledge, the goal of model debugging via interpretability, similar to the tests conducted in natural imaging (Adebayo et al., 2020), is overlooked in the current neuroimaging literature, as is also the case in oncology (Amorim et al., 2021). However, model debugging may help diagnose model misbehavior and provide new insights into brain function. Importantly, given the limitations of current XAI approaches as described above in generating local (individual-level) explanations, which are only superficially understandable, justifying individual decisions based on these explanations may be unreliable and more work is needed to use them reliably for individual-level decisions in clinical practices. This aligns with Ghassemi et al. (2021)’s viewpoints, highlighting the limitations of existing explainability approaches. The authors argued that while existing approaches may provide a global (common) understanding of the model’s behavior and can be useful in the model development process, these methods, in their current forms, are not able to offer reliable justification for individual-level decisions. This is mainly because the existing post hoc interpretability mostly points to where a model is looking, not what it is doing with these parts (Rudin, 2019).

*(i) Studies have not considered using the example-based explanation approach:* The concept of using influence functions (Koh & Liang, 2017), an example-based explanation method, could be valuable for the neuroimaging community. Patients from previous records may have neurologically similar traits to the patient requiring treatment. By grouping these patients into coherent clusters, significant information could be revealed to scientists and clinicians, aiding in a better understanding of disorders and the development of personalized treatment plans. For example, instead of using group-level brain maps computed from individual explanations (Thomas et al., 2019), more nunanced group-level maps can be generated based on representative training samples (i.e., examples as explanation), aiding patient-specific clinical decisions.

*(j) Neuroimaging studies have not considered using concept-based explanations approach:* Concept-based explanations can be helpful for the model developers, regulators, and clinicians (Imrie et al., 2023). These explanations may help stakehoders know whether the model used or understood established medical concepts. For example, Rahman et al. (2022) reported that the model was able to capture cerebellum interaction across multiple domains and sensorimotor changes, generally considered as a consistent pattern for schizophrenia. Qiu et al. (2020) also identified patient-specific probability maps of the brain for AD diagnosis and disease-specific neuroimaging signatures. Indeed, they confirmed AD probability risk by showing a high grade of amyloid-β and tau accumulation in the hippocampal formation, the middle frontal region, the amygdala, and the temporal region (Qiu et al., 2020). Neuroimaging community may devise ways, similar to the usage of known concepts in diabetic retinopathy (Kim et al., 2018), to reliably verify whether the model learned these established biomarkers.

*(k) The fairness of explanations is yet to be investigated in neuroimaging:* Many studies have pointed out fairness issues in existing XAI approaches, raising concerns that models may be biased toward certain subgroups. For example, Balagopalan et al. (2022) investigated the fairness issue of two post hoc local explanation methods, LIME and SHAP. They found that LIME-generated explanations have larger fidelity gaps between subgroups (Balagopalan et al., 2022), meaning that the quality of explanations varies depending on class membership, making them unfair to subgroups. While many DL-based neuroimaging studies have used SHAP (Ball et al., 2021; Gaur et al., 2022; Lombardi et al., 2021) and LIME (Gaur et al., 2022; Hossain et al., 2023; Lombardi et al., 2021; Magesh et al., 2020; Saboo et al., 2022), to the best of our knowledge, the neuroimaging community has not yet focused on the fidelity gaps of explanations between subgroups. Since fidelity can be a serious concern for model-agnostic approaches (Balagopalan et al., 2022), we encourage practitioners to use reliable fidelity metrics (Balagopalan et al., 2022; Yeh et al., 2019) and related strategies (Pradhan et al., 2022) to identify fairness concerns among subgroups such as male and female subjects, patients and controls, and among categories of patients, as these issues have direct implications for downstream decision making.

### 5.3 Advantages and disadvantages of post hoc XAI methods

In this section, we discuss the relative advantages and disadvantages of post hoc methods so that practitioners can make an informed decision when selecting a post hoc method that aligns with their end goals. However, it is important to recognize that post hoc explanations have inherent limitations. They cannot reliably indicate when a model’s prediction is incorrect and often provide only sufficient evidence supporting a prediction rather than offering a complete account of the model’s decision process—an aspect essential for deriving personalized and actionable insights (Leonardsen et al., 2024). Furthermore, it is common practice to generate explanations only for the predicted (correct) class, which can be misleading (Rudin, 2019).

Attribution-based explanations (based on gradient or perturbation-based approaches) are preferred over counterfactual, example-based, or concept-based explanations because they are more easily understandable to humans (Yeh et al., 2019). However, compared with perturbation-based methods, gradient or modified gradient-based attribution methods are faster, easier to implement, and readily applicable (Sundararajan et al., 2017) to DL models. In other words, model-specific methods (backpropagation-based or intrinsic methods) are much faster than model-agnostic approaches (based on perturbation and/or proxy interpretable models) (Chen et al., 2022b). For example, gradients, gradients ⊙ input, %  -LRP, DeepLIFT are computationally very efficient. However, *integrated gradients* is slower because it computes integration numerically, requiring multiple forward passes with small variations in input and computing gradients. Nonetheless, IG is usually faster than other perturbation-based approaches and was applied to many neuroimaging studies (Rahman et al., 2022; Wang et al., 2023a; Zeineldin et al., 2022). DeepLIFT can be a computationally efficient alternative to IG for feed-forward networks, but fails to produce meaningful explanations for recurrent networks (Ancona et al., 2017). In neuroimaging, this method was used only in a few studies based on our review (Gupta et al., 2019; Hu et al., 2021b). Many attribution methods, such as integrated gradients and DTD, require a suitable baseline to determine the quality of explanations (Kindermans et al., 2019). However, the criteria of selecting an appropriate baseline are still not well defined (Ancona et al., 2017).

While Grad-CAM or its “guided” version has been extensively used in neuroimaging studies (Dyrba et al., 2020a; Esmaeili et al., 2021; Hu et al., 2021d; Kan et al., 2021; Lin et al., 2022a; Natekar et al., 2020; Tang et al., 2019; Wang et al., 2023a; Yang et al., 2018; Zeineldin et al., 2022; Zhang et al., 2021b), this method produces coarse explanations and may generate misaligned explanations in the input space (Xia et al., 2021). In neuroimaging, Uzunova et al. (2019) reported that Grad-CAM identified the infected regions but suffered from poor resolution. For segmentation and localization tasks, many studies found Grad-CAM to be discriminative (Esmaeili et al., 2021; Zeineldin et al., 2022). However, Grad-CAM cannot help track smooth changes in brain regions associated with underlying disorders. Interestingly, while we did not find any application of the FullGrad (Srinivas & Fleuret, 2019) method in neuroimaging, this method addresses some limitations of traditional gradient-based approaches by considering both local importance in the input space and global importance (i.e., the importance of neurons) in a neural network.

Explanations generated using some post hoc methods do not change when model parameters are gradually randomized (Adebayo et al., 2018), causing these methods to fail in capturing class-discriminative signals in the data. For example, guided methods, such as Guided Grad-CAM (Selvaraju et al., 2017) and GBP (Springenberg et al., 2014), are insensitive to model parameters and behave like edge detectors (Adebayo et al., 2018). Nie et al. also reported that GBP and DeconvNet essentially perform partial image recovery and cannot produce class-sensitive explanations (Nie et al., 2018). Sixt et al. (2020) further showed that all modified backpropagation-based approaches, except DeepLIFT, such as DTD, LRP, GBP, and DeconvNet, are independent of the parameters of the later layers. Shrikumar et al. (2017) reported that GBP and DeconvNet discard negative gradients during backpropagation, thus losing their ability to maintain class-sensitive information. Moreover, methods such as DeconvNet, GBP, and LRP cannot produce theoretically correct explanations, as demonstrated by Kindermans et al. (2017). Gradient and Grad-CAM, however, are sensitive to the model parameters, and computationally efficient, but gradient-based methods rely on noisy gradients (Ancona et al., 2019; Smilkov et al., 2017). Many neuroimaging studies often use vanilla gradients as a baseline in their benchmarks (Essemlali et al., 2020; Hu et al., 2021b; Lin et al., 2022a; Oh et al., 2019; Rieke et al., 2018; Zeineldin et al., 2022).

Arras et al. (2022) evaluated several post hoc methods based on a visual question answering task and found results that are often contradictory from earlier studies (Adebayo et al., 2018; Hooker et al., 2019; Sixt et al., 2020). In their evaluation, LRP, IG, GBP, and Guided Grad-CAM (due to GBP) were the top-performing methods, while Grad-CAM and DeconvNet performed the worst. The other gradient-based approaches, such as gradient, gradient ⊙ input, and SmoothGrad, maintained a moderate level of performance. While our reviewed neuroimaging studies obtained similar results, they were not sufficiently conclusive. For example, IG heatmaps were found to be more consistent than guided Grad-CAM and LRP heatmaps for AD classification (Wang et al., 2023a). Many studies used GBP (Böhle et al., 2019; Dyrba et al., 2020a; Hu et al., 2021d; Kan et al., 2021; Rieke et al., 2018; Zeineldin et al., 2022) in neuroimaging but reported poor performance, similar to findings in natural imaging and contrary to the results reported by Arras et al. (2022). For example, Böhle et al. (2019) reported that GBP produces low-contrast group-wise maps, whereas Uzunova et al. (2019) found this method to be noisy and not class discriminative. Additionally, Dyrba et al. (2020a) found that Grad-CAM and GBP generated scattered maps that were loosely coupled with the literature. Hence, there is still debate about GBP’s performance in the literature.

Despite some issues mentioned earlier, LRP has several advantages. It breaks down individual feature contributions and does not rely solely on the model’s sensitivity as gradients do. Moreover, LRP provides a more intuitive interpretation by preserving attribution conservation across all layers, whereas methods such as IG, DeepLIFT, and SHAP conserve total attribution only at the input–output level, not within intermediate layers (Leonardsen et al., 2024). In addition, LRP can be computationally efficient compared with gradient and occlusion-based methods. LRP does not suffer from noisy gradients and accounts for global feature interactions, unlike occlusion (Hofmann et al., 2022). However, selecting appropriate propagation rules determines the quality of explanations. While neuroimaging community used different LRP rules in various studies (Böhle et al., 2019; Dyrba et al., 2020a; Eitel et al., 2019b; Hofmann et al., 2022; Thomas et al., 2019; Wang et al., 2023a; Zhao et al., 2022) with less obvious justification, Dyrba et al. (2020a) showed that DTD and LRP (α=1, β=0 rule) were able to produce clinically valuable results for AD detection. Arras et al. (2022) also reported the best performance for LRP with the α=1, β=0 rule for the hidden layers and the box-rule for the input layer. Although several neuroimaging studies used the %-rule (Eitel et al., 2019b; Thomas et al., 2019), composite rule (Hofmann et al., 2022), and LRP-β rule (Binder et al., 2016)—a different formulation of the LRP-αβ rule (Wang et al., 2023a; Zhao et al., 2022)—Böhle et al. (2019) argued that the β-rule with lower values of β, which focuses more on positive contributions, might generate heatmaps suitable for AD detection tasks, as AD affects brain regions in a localized manner. For more on rule selection, we refer to Montavon et al. (2019)’s work. We note that LRP has other limitations. For example, a strong link between LRP (z-rule or %-rule) and “gradient ⊙ input” was found when max-pooling, linear layers, and ReLU were used (Ancona et al., 2017; Shrikumar et al., 2017). In particular, %-LRP is equivalent to Gradient ⊙ Input, if the network has only ReLU nonlinearities (Ancona et al., 2017). Sixt et al. (2020) also reported that ${\text{LRP}}_{\alpha \beta }$ , which looks for both positive and negative contributions, fails to be class sensitive, leading to unclear conclusions about its superior performance over other methods as reported by Arras et al. (2022) and Dyrba et al. (2020a).

Smoothgrad lowers both sensitivity and infidelity (Yeh et al., 2019), and we found some neuroimaging studies that used this method (Levakov et al., 2020; Rahman et al., 2022; Zeineldin et al., 2022). Smoothgrad was able to find discriminative regions and can be robust to noise (Zeineldin et al., 2022). However, this method cannot provide information about the direction of contribution (Levakov et al., 2020) as opposed to gradients. Rahman et al. (2022) used Smoothgrad with base IG explanation for multiple brain disorders and reported more stable explanations.

While perturbation-based methods are computationally intensive, they have some obvious advantages: (1) interpretation of results is straightforward as these approaches calculate the marginal effect of a feature or a set of features and (2) these approaches are model-agnostic and can be applied to almost any model. Although many neuroimaging studies (Abrol et al., 2020; Oh et al., 2019; Rieke et al., 2018; Tang et al., 2019; Wang et al., 2021a; Yang et al., 2018) used the *Occlusion Sensitivity* method to find class-discriminative features, this method produces unstable maps (Ancona et al., 2019) and misses the semantic organization of brain regions (Oh et al., 2019; Yang et al., 2018) as distributed relevance is not possible (Rieke et al., 2018; Yang et al., 2018) because of its adjacent neighborhoods and arbitrary grid sizes (Yang et al., 2018). However, *Meaningful Perturbation* is able to generate stable maps in neuroimaging and has been used in a few studies (Kan et al., 2021; Uzunova et al., 2019). Another concern of perturbation-based approaches is that they modify the input distributions, which eventually changes the model’s outcome (Arras et al., 2022). Additionally, the choice of underlying perturbation technique significantly affects the explanations.

Distillation methods, such as LIME and Kernel SHAP, also have similar disadvantages as observed in perturbation-based methods because these methods require perturbing inputs and evaluating models at those perturbations before fitting with an interpretable model. Moreover, LIME (Ribeiro et al., 2016) generates coarse explanation maps (Fong & Vedaldi, 2017) and is unstable with hyperparameter and perturbation changes. The use of LIME for differentiable models also raises an additional concern, as argued by Lipton (2018). SHAP has significant limitations: it assumes feature independence, an unlikely assumption that can result in unrealistic permuted instances. Additionally, SHAP is suboptimal (Kwon & Zou, 2022) as it assigns equal weights to all marginal contributions for a feature, potentially leading to attribution errors when different marginal contributions have varying levels of signal and noise. While multiple neuroimaging studies used SHAP (Ball et al., 2021; Gaur et al., 2022; Lombardi et al., 2021) and LIME (Gaur et al., 2022; Hossain et al., 2023; Lombardi et al., 2021; Magesh et al., 2020; Saboo et al., 2022), SHAP was often used to ensure consistency and accuracy in explanations (Lombardi et al., 2021), whereas LIME was used to understand the local behavior around a test sample (Gaur et al., 2022). We note that LIME and SHAP do not guarantee *sensitivity* and *implementation invariance*—two desirable characteristics of post hoc methods that we discuss later. Thus, distillation methods may be unfaithful to the original model (Rudin, 2019). However, SHAP can be more faithful to the model than LIME (Yeh et al., 2019). Refer to Table 9 for more opinions and takeaways about XAI practices.

*Desirable Characteristics (Axioms) of Post Hoc Methods:* To evaluate the reliability of post hoc methods, many studies define certain desirable behaviors (axioms) that these methods are expected to satisfy. Some of these axioms include:

- *Sensitivity(a)* (Sundararajan et al., 2017) implies that if a feature can alter a baseline prediction, it should receive a non-zero attribution. Gradients, DeConvNets, and Guided Backpropagation violate *Sensitivity(a)*, whereas DeepLIFT, LRP, and IG satisfy this property.
- *sensitivity-N* (Ancona et al., 2019), also known as *completeness* (Sundararajan et al., 2017) or *summation to delta* (Shrikumar et al., 2017), requires that the sum of the attributions of the features plus the baseline output score equals the target output score. IG and DeepLIFT (in a feed-forward network) satisfy this property.
- *Sensitivity(b)* (Sundararajan et al., 2017) requires that if the output of a network does not depend on a feature, then that feature should receive zero attribution. IG satisfies this property.
- *Linearity* (Sundararajan et al., 2017) requires that if a model function is a linear composition of two other model functions, then the attribution of the composite model should be the same linear composition of the base models’ attributions. IG satisfies this property.
- *Symmetry-preserving* (Sundararajan et al., 2017) requires that if swapping the values of two features does not change the output function, then the two features should receive the same attributions, provided their values in the input and their baseline values are also the same. IG satisfies this property.
- *Implementation invariance* (Sundararajan et al., 2017) requires that identical explanations be produced by all functionally equivalent models, regardless of their implementations. LRP and DeepLIFT violate this property, whereas Gradient and IG satisfy this characteristic.
- *Input invariance* (Kindermans et al., 2019) requires that explanations reflect the sensitivity of the model in response to input transformations. Gradient and signal-based methods (e.g., GBP) satisfy this property, while SmoothGrad satisfies it conditionally (depending on the underlying base method). Gradient ⊙ Input does not satisfy *input invariance*, but IG and DTD satisfy it conditionally (based on reference points).
- *Explanation continuity* (Montavon et al., 2018) requires that explanations for two nearly identical data points with identical model responses be also identical. Gradient-based methods and some modified backpropagation-based methods (e.g., %-LRP, DeepLIFT (Rescale)) are likely to violate this property. However, some variants of LRP (e.g., αβ rule, deep Taylor LRP) (Yeh et al., 2019) and DeepLIFT (RevealCancel rule) (Shrikumar et al., 2017) address this issue.

Although these axioms are desirable, we did not find any neuroimaging study that focused on justifying the use of post hoc methods based on these characteristics. Finding post hoc approaches that satisfy these axioms or developing directly interpretable models for neuroimaging is challenging due to high-dimensional data, data scarcity, data privacy issues, variations in data acquisition methodologies, and the use of multi-modalities. However, we acknowledge their importance in building more reliable approaches. We also suggest developing explainability methods that can be rigorously validated through strong collaboration among researchers and by leveraging multi-site data.

### 5.4 Additional recommendations for DL practices

In this section, we offer additional recommendations for exploring XAI avenues in neuroimaging. These suggestions stem from the fact that DL models are fragile and the current explanation methods are inadequate. Additionally, there is a lack of guiding principles in XAI, a need for rigorous model validation, and the requirement for suitable metrics. To gain fresh perspectives from well-considered future XAI practices, we propose the following overarching recommendations, in addition to the research gaps identified in Section 5.2, for the neuroimaging community.

1. Beware of the transparency notion for model interpretability: The notion of interpretability is often ignored when choosing between linear and deep models. A pervasive opinion is that linear models are inherently interpretable. However, linear models usually provide algorithmic transparency for high-dimensional or heavily engineered features. In those scenarios, only the learning algorithm is interpretable for linear models. For high-dimensional features, linear models lose simulatability, that is, humans are unable to make it computationally tractable. For highly engineered features, linear models may achieve comparable performance as deep models but compromise decomposability (Lipton et al., 2016), that is, the input features may no longer remain intuitively meaningful. Conversely, DL models preserve decomposability at the cost of algorithmic transparency. Also, DL models can learn rich representation from the data, and post hoc explainability approaches may reveal important insights.
2. Leverage the power of model ensembles: Neural networks, due to their distinct computational processes during training, tend to learn different facets of the disorder. Consequently, even when models use “true” evidence, explanations and their relative significance can vary. While studies (Hofmann et al., 2022; Hossain et al., 2023; Leming et al., 2020; Levakov et al., 2020) have applied model ensembles in design, some of them focused on the uncertainty behavior of the models to improve their robustness (Hofmann et al., 2022; Levakov et al., 2020). By effectively consolidating explanations from diverse models and initializations, valuable insights regarding the disorders can be revealed (Levakov et al., 2020).
3. Beware of shortcut learning and develop robust out-of-distribution (o.o.d) tests: DL models are prone to shortcut learning as they tend to seek the easiest solution and may rely on unintended spurious correlations (Adebayo et al., 2022; Geirhos et al., 2020; Lapuschkin et al., 2019). Understanding the influence of model architecture, training data, loss function, optimization parameters, and initialization parameters can reveal the nature of shortcuts. Expert domain knowledge can also aid in identifying these undesirable behaviors. Whenever possible, we should perform extended validation on mulit-site, multimodal models, and multiple datasets as suggested in several studies (Qiu et al., 2020; Viswan et al., 2024) because the model may not generalize effectively to real-world datasets due to shifts in distribution (Geirhos et al., 2020). Therefore, it is important to develop suitable out-of-distribution tests (Geirhos et al., 2020). For instance, Leming et al. (2020) demonstrated that data from multiple sites may increase performance of DL models but may compromise interpretability. Another possibility is to incorporate additional structure and appropriate inductive biases so that trained models have sufficient shared components with the new environments, as suggested by Goyal and Bengio (2022). These additional inductive biases about distributions and their change patterns can be incorporated into the training via new architectures and learning strategies. Along these lines, there is still a need to devise useful inductive biases and optimization algorithms for model training to gain out-of-distribution generalizability in neuroimaging.
4. Efficiently deal with data imbalance, data scarcity, and missing data: Neuroimaging studies often face challenges such as data imbalance, data scarcity, and distributional instability, which may compromise the accuracy of a model. Transfer learning, data augmentation techniques (Afzal et al., 2019), under/over sampling data, using class-weighted loss function, “stratified cross-validation,” evaluating on balanced hold-out sets, significance testing of the performance estimates, and using task-relevant performance metrics may help evaluate the generalizable performance of the models (Thölke et al., 2023).
  Data Augmentation: While transfer learning approaches may largely mitigate the data scarcity issue (see Table 4 and the last recommendation), techniques such as self-supervised data augmentation (Smucny et al., 2022), image-to-image translation, brain MRI reconstruction, and generating samples via domain-supported counterfactual explanations (Abrate & Bonchi, 2021; Liu et al., 2021; Oh et al., 2022b; Pombo et al., 2023) may help augment the dataset and can be very effective in improving the generalizability of the models.
  Complete Missing or Incomplete Data: Missing or incomplete data are very common in longitudinal studies, as some subjects might not continue participation (Martí-Juan et al., 2020), or in multimodal studies, where some modality might be missing (Zhang et al., 2020b). Studies used diverse techniques based on the missing patterns, including removing incomplete data if the sample size is not too small (Zhou et al., 2013), using suitable imputation techniques such as predicting one modality from another (Li et al., 2014; Pan et al., 2018), developing methods robust to missing data (Ghazi et al., 2019), and using simulated data (Bowles et al., 2018; Jung et al., 2023; Pombo et al., 2023; Ravi et al., 2022) to resolve the issue. An additional advantage of simulated data is that it allows us to artificially create scenarios to verify the robustness of the models toward missing data or data imbalance (Martí-Juan et al., 2020).
  Use suitable metrics: The choice of performance metrics depends on the specific problem and the performance estimates that suit the problem. For classification tasks, metrics such as “area under the curve,” “balanced accuracy” might be suitable. In contrast, for detection problems (e.g., brain tumor), precision and recall may be a good choice. We note that while all these strategies help mitigate data scarcity issues, DL models still need well-curated and labeled data (Górriz et al., 2023).
5. Beware of traditional sanity checks—model randomization tests are not reliable: Binder et al. (2023) argue that the top–down randomization, a.k.a “cascaded randomization,” test is methodically similar to faithfulness test in the way that the model randomization test obscures the parts of the model and the model faithfulness test obscures the parts of the input while computing their respective scores, and hence they are comparable. However, the authors demonstrated that their rankings of explanation methods vary widely. Another issue of model randomization test is while randomization leaves parts of the model unchanged, the cascaded randomization severely influences the model’s outcome. While model randomization tests might serve as a quick sanity check, the rankings based on this approach is not reliable (Arras et al., 2022). Arras et al. (2022) pointed out that the evaluation setup used in randomization test or any perturbation-based evaluation is prone to errors as they either rely on modified data distribution or on modified model weights.
6. Apply suitable explainability tool for multi-site, multimodal data: Generalized DeepSHAP (Chen et al., 2022b) might be useful for debugging models trained on multi-site datasets or sites using different models for the same problem to generate reliable explanations from a series of models (Chen et al., 2022b) trained in distributed settings or trained separately at their respective sites. In this case, model-agnostic approaches will require access to all the models, which is impractical. As this method propagates local feature attributions to group attributions, it can also be effective to generate more representative explanations from multimodal neuroimaging. These proposals could be beneficial for the neuroimaging community, as data scarcity, data privacy, and leveraging the potential of multimodal fusion are determining factors in the successful application of DL models.
7. Keep in mind the absence of a reliable guiding principle: Although various explanation methods have been used in studies, there is limited theoretical evidence or guiding principle to determine which method to choose. Some studies (Han et al., 2022; Krishna et al., 2022) have shown that different explanation methods yield different explanations by describing different neighborhoods, thus rendering them unreliable (Chan, 2021; Hatherley et al., 2022; John-Mathews, 2021; Lakkaraju & Bastani, 2020; Laugel et al., 2019a, 2019b, 2019c; Leslie, 2019; Loi et al., 2021; Loyola-Gonzalez, 2019; Páez, 2019; Rudin & Radin, 2019; Rudin, 2019; Slack et al., 2020). Han et al. (2022) also demonstrated that the popular post hoc methods usually provide local function approximation of a model. The authors also provided valuable guidance on selecting interpretable methods based on the characteristics of the data. The neuroimaging community can refer to the authors’ suggestions for selecting XAI methods for various data modalities. Additionally, if these suggestions are not considered appropriate for neuroimaging, researchers may be motivated to create new guiding principles tailored specifically for neuroimaging data.
8. Improve data preprocessing and find modality/data-specific metrics: Given the complex and high-dimensional nature of neuroimaging data, effective data preprocessing strategies can help better identify informative biomarkers that drive a model’s decisions and significantly reduce computational costs (Górriz et al., 2023). However, we caution that post hoc methods, mostly developed for natural images, have been found unreliable in multiple studies (Arun et al., 2021; Kindermans et al., 2019; Rudin, 2019) for localizing abnormalities in medical images. The complex clinical decision-making process based on multimodal data could easily be overlooked by the model (Jin et al., 2022). Therefore, we need evaluation metrics consistent with the domain and data to guide the design and selection of new algorithms.
9. Use structure–function fusion model for model diagnosis and new insights: Earlier studies often focused either on the anatomical or functional aspects of dynamics. However, a unified framework that incorporates both modalities and utilizes existing anatomical and functional knowledge for rigorous validation of the explanations may offer valuable insights for designing clinically reliable AI. Moreover, multimodal systems often outperform unimodal systems in terms of accuracy and allow us to provide multiple viewpoints for a better understanding of the brain (Górriz et al., 2023). While some studies used multimodal fusion in neuroimaging (Hu et al., 2021d; Qiu et al., 2020), there is still scope to leverage XAI in multimodal studies for an additional level of validation and for gaining new insights.
  The structure–function fusion, for example, combining sMRI and fMRI modalities, approach is generally inspired by studying how changes in brain structure are related to dynamic brain function. Earlier studies (Batista-García-Ramó & Fernández-Verdecia, 2018; Honey et al., 2007, 2009; Koch et al., 2002) have shown that, if estimated over larger time windows, functional connectivity (FC) correlates with structural connectivity (SC). That is, structurally connected cortical regions exhibit stronger and more consistent resting-state functional connectivity (rs-FC).
  Another study by Diez et al. (2015) confirmed that rs-FC and SC networks have a modular configuration and they found an excellent match between structural and functional modules. A study by Nakagawa et al. (2013) demonstrated that SC decline in aged people lowers the complexity of the blood-oxygen-level-dependent (BOLD) signals. The authors argued that cognitive performance decline in elderly people can be explained by the resulting higher overall activation to compensate for lower communication efficiency. Another study by Plis et al. (2018) leveraged the idea of machine translation and proposed a DL model with an attention mechanism (Bahdanau et al., 2014) to capture the links and their strengths between the well-established features obtained from the dynamic FC and SC associated with healthy controls and schizophrenia patients.
  Given the availability of a large number of multimodal imaging datasets for the same subjects, we can leverage this encouraging and conclusive structure–function correspondence knowledge from prior studies to gain additional benefits of explainability from the data-driven approaches of the multimodal fusion model. The first potential benefit is to allow the model to learn a richer representation (Plis et al., 2018) of the data from multiple modalities containing various dynamic and static information, hence presumably making the model more capable of providing deeper insights. These important relationships cannot be recovered from a single-modal study (Calhoun & Sui, 2016). Second, the structure–function fusion model can offer the advantage of an additional layer of validation of explanations, making the model more trustworthy in clinical deployment and scientific advancement. Third, we can reduce misdirection in our investigation and conclusions (Plis et al., 2011) and potentially find the complex links between structure and function, similar to what was pointed out by Calhoun and Sui (2016). For example, we believe highly attributed regions in the structural input should reasonably (as known in the literature) conform to the highly attributed functional features as a way of extended validation of the model’s learned behavior. This conformity can easily be verified based on the structural and functional connectivity computed using the generated explanation masks (Rahman et al., 2022). We are aware that multimodal fusion models may not rely on both modalities in many training scenarios and may require continual learning for better interpretation of results. Nevertheless, to fully realize the potential benefits of multimodal approaches through XAI, we suggest a baseline requirement: trained models should reasonably rely on both modalities to provide meaningful insights, enabling us to derive conclusions reliably from the explanations.
10. Harness the power of counterfactuals for causality and biological mechanisms: *Counterfactual* explanations (Dandl et al., 2020; Wachter et al., 2017) provide insights into the hypothetical realities that could potentially alter the decisions made by a model. In the field of neuroimaging, we believe that *counterfactual* explanations can greatly contribute to our understanding of the underlying biological mechanisms associated with the initial manifestation of specific disorders. By examining these explanations within various hypothetical scenarios, we may also be able to identify causal relationships underlying these disorders. Counterfactuals may also help clinicians proactively manage patients’ disorder trajectories at a causal level. While gradient-based approaches have limited counterfactual reasoning ability (Oh et al., 2022b), generative models can be an excellent tool for producing realistic counterfactual explanations, as observed in multiple studies (Bowles et al., 2018; Jung et al., 2023; Pombo et al., 2023; Ravi et al., 2022).
11. Pay attention to the confirmation bias inherent in humans: Humans, by nature, tend to create narratives due to confirmation bias. Thus, the crucial question is whether the model learned to predict for the right reasons. This is critical because ML models solely focus on correlations and lack knowledge of truths or underlying causes. They might mistakenly designate proxy or less significant variables as highly important, even if they are only loosely correlated with the actual cause. Additionally, ethical issues need to be addressed as human biases can compromise the transparency of interpretable systems (Herman, 2017).
12. Analyze interpretations from various task scenarios: Over the years, studies have mainly focused on interpreting supervised models such as classification and regression models. However, interpreting the hidden knowledge acquired in unsupervised settings, such as clustering models, remains relatively unexplored (Crabbé & van der Schaar, 2022b). In the field of neuroimaging, there have been only a few studies that conducted projection transformation from the latent space to observe the area of influence (Biffi et al., 2020; Martinez-Murcia et al., 2019).
13. Investigate the effectiveness of transfer learning in neuroimaging: Neuroimaging studies have utilized transfer learning using different approaches: unsupervised (Oh et al., 2019), self-supervised (Kim et al., 2023; Rahman et al., 2022), natural imaging to brain imaging (Ban et al., 2022; Chen et al., 2020; Demyanchuk et al., 2019; Etminani et al., 2021; Gao et al., 2022; Hossain et al., 2023; Magesh et al., 2020; Ni et al., 2021; Oktavian et al., 2022), healthy controls to target task (Lu, 2022), and one task to another (Gao et al., 2022; Nguyen et al., 2020; Thomas et al., 2019) (refer to Table 4). However, the specific knowledge being transferred and the factors contributing to improved performance remain unclear (Raghu et al., 2019). Raghu et al. (2019) pointed out that natural images and medical images are different and that the source and target models solve different problems. This ambiguity also applies to transferring knowledge from one disorder to another. Additionally, transfer learning may introduce negative transfer to the downstream model, potentially leading to unfair decisions or explanations if the source domain and the target domain are not similar (Weiss et al., 2016). We need to identify the shared properties between the source and target distributions, that is, determining which parts remain stable and which parts may differ in both environments. We found only one study (Oliveira et al., 2024) that quantitatively benchmarked explanation performance of many XAI methods on two pretrained VGG-16 models, one trained on natural images and the other on MRI slices. Their findings reveal that the performance of popular post hoc XAI methods varies widely. This variation is strongly tied to the pre-training task and the parts of the network being pretrained. This study used synthetically created class-discriminative lesions (e.g., white matter hyperintensities) to serve as the ground-truth explanation for the validation. While the results are not yet conclusive (deconvolution and GBP performed poorly), the effectiveness of transfer learning on both predictive and explanation performance warrants further investigation.

## 6 Conclusion

In this article, we reviewed 122 papers from the general XAI domain and comprehensively introduced the field to a broader neuroimaging community, especially to help newcomers. We discussed the interpretability problem, taxonomy, XAI methods, evaluation metrics, commentaries on the existing approaches, and available toolkits with their properties that neuroimaging practitioners can readily use for their study goals. In the neuroimaging domain, we reviewed 409 papers that utilized DL technology and adapted model interpretability to interpret their findings. To gain more insights about the existing practices, we conducted a meta-analysis of the reviewed neuroimaging papers using post hoc methods and identified the predominant approaches, neuroimaging modalities, and applications. Finally, we identified the critical gaps and offered valuable recommendations to effectively face the ongoing challenges.

While our meta-analysis revealed that Grad-CAM and its variants (Chattopadhay et al., 2018; Selvaraju et al., 2017; Zhou et al., 2016), SHAP (Lundberg & Lee, 2017), IG (Sundararajan et al., 2017), LRP (Bach et al., 2015), Occlusion Sensitivity (Zeiler & Fergus, 2014), and GBP (Springenberg et al., 2014) have been predominantly used in neuroimaging, this trend does not necessarily indicate their reliability. Moreover, studies have reported contradictory conclusions about post hoc methods, as discussed in Arras et al. (2022)’s work and other investigations (Adebayo et al., 2018; Hooker et al., 2019; Sixt et al., 2020), especially for GBP. So, we suggest a more systematic evaluation of post hoc approaches in neuroimaging using extended validation datasets and reliable explanation evaluation metrics, rather than fully relying on earlier conclusions (Adebayo et al., 2018; Hooker et al., 2019; Sixt et al., 2020). While some studies (Deshpande et al., 2024; Munroe et al., 2024) recommend using multiple methods to find common features, we argue that this approach might lead to more confounding conclusions because the methods examine different aspects of the model and have their own strengths and weaknesses. Instead, benchmarking popular and theoretically promising methods separately on a large pool of datasets may help independently assess their reliability. Subsequently, consensus merging based on complementary insights can be useful (Thibeau-Sutre et al., 2023). For proprietary models, separate interpretable models (LIME and Kernel SHAP) or other model-agnostic approaches (e.g., Occlusion, meaningful perturbations) may be the only options. However, we caution that these interpretable models may be completely unfaithful to the original model, and the interpretations may not reflect what the models actually learned.

While “example-based” explanations are yet to be investigated in neuroimaging, we believe that exploring this direction will allow us to stratify patients based on their common traits and symptoms. Moreover, “concept-based” explanations, which are also unexplored in neuroimaging, can be a great tool to test whether DL models rely on known concepts or new associations for their decisions. The latter case (i.e., models learning new associations) may make the model a suitable candidate for thorough examination for potential new insights.

Though developing intrinsic methods may be time consuming and require a higher level of expertise in neuroimaging and DL, we strongly encourage these approaches to generate explanations that are inherently faithful to model predictions. These methods are promising because they emphasize design transparency and alignment with the model’s behavior. Counterfactual explanation, a form of intrinsic methods, may further assist in understanding the underlying mechanisms of brain changes associated with disorders (Abrate & Bonchi, 2021; Liu et al., 2021; Oh et al., 2022b). However, the development of intrinsic approaches remains challenging, often depending on hyperparameter choices in the architecture design and on the increased difficulty of interpreting the resulting explanations (Adey et al., 2024). Moreover, there is currently no domain-specific mechanism to regulate which concepts the model learns from the data.

While many studies we reviewed performed quantitative validation (see Table 3) of the generated explanations, the metrics are mostly chosen based on subjective intuitions. Moreover, the existing XAI tools (see Table 2) and evaluation metrics (see Section 3.2.2) are mostly developed in the computer vision domain and focused on natural images. Therefore, there is an urgent need to improve upon these tools and metrics so that a comprehensive and reliable evaluation of XAI approaches in neuroimaging becomes possible.

## Data Availability

The paper is the data: no new data or code was generated for the research described in the article.
